# Mild photothermal-responsive hydrogel with H_2_Se delivery for anti-infection and microenvironment remodeling to bone regeneration in diabetic osteomyelitis

**DOI:** 10.1016/j.bioactmat.2026.04.041

**Published:** 2026-05-02

**Authors:** Junwei Su, Xinyue Liang, Junwei Yang, Xianzhen Dong, Yu Chen, Xinyi Tan, Zhiqiang Li, Yuchen Song, Hao Zhang, Honglian Dai, Aixi Yu

**Affiliations:** a Department of Orthopedics, Zhongnan Hospital of Wuhan University, Wuhan, 430071, PR China; b State Key Laboratory of Advanced Technology for Materials Synthesis and Processing, Wuhan University of Technology, Wuhan, 430070, PR China

**Keywords:** Infected bone defects, H_2_Se gas therapy, Antibacterial, Immunoregulation, Osteogenesis

## Abstract

Diabetic osteomyelitis, exacerbated by a hyperglycemic microenvironment, leads to increased bacterial infections and bone tissue destruction, raising the risk of amputation. Current antibiotic therapies are limited in effectiveness due to rising antibiotic resistance and biofilm barrier. In response, we developed a microgel-based hydrogel system that delivers H_2_Se gas combined with mild photothermal therapy (MPTT) to achieve integrated treatment for infection control, anti-inflammation, and osteogenesis. This system utilizes Fe_3_O_4_ nanoparticles as photothermal-responsive carriers to encapsulate the H_2_Se donor TDN1042, which is constructed into lipoic acid-modified gelatin (Gel-LA) microgels using microfluidic technology. Upon in situ injection, photocrosslink forms the TF@GL hydrogel that enables triple modulation under 808 nm NIR. First, MPTT promotes H_2_Se release, disrupting bacterial metabolic homeostasis and lysing biofilms. Second, H_2_Se scavenges excess reactive oxygen species (ROS) to alleviate cell death, simultaneously inhibiting inflammation pathways (NF-κB and NLR). Lastly, the TF@GL hydrogel promotes osteogenic differentiation by activating the TGF-β/BMP osteogenic pathway, and the porous structure enhances cell migration and nutrient diffusion, accelerating bone repair. This design provides a comprehensive therapeutic strategy for diabetic osteomyelitis through the spatiotemporal synergy of gas delivery and controlled photothermal effects, offering effective antibacterial activity, oxidative stress alleviation, and bone regeneration induction.

## Highlights

• A microgel-based hydrogel integrating H_2_Se delivery with mild photothermal therapy for diabetic osteomyelitis treatment.
• NIR-triggered H_2_Se release inhibits bacterial metabolism and disrupts biofilm to achieve rapid bacterial eradication.
• Delivery of H_2_Se gas suppresses the NF-κB and NLR pathways to improve the immune microenvironment.
• Hydrogel activates the TGF-β/BMP signaling pathway to promote osteogenic differentiation of stem cells.

## Graphical abstract

This study addresses the challenges of infection and inflammation in diabetic osteomyelitis by developing a mild photothermal-responsive TF@GL hydrogel with H_2_Se gas delivery. This hydrogel enables the controlled release of H_2_Se to combat infection, reduce oxidative stress, alleviate inflammation, and promote osteogenesis in diabetic cranial osteomyelitis, ultimately enhancing bone regeneration.

**Figure:**
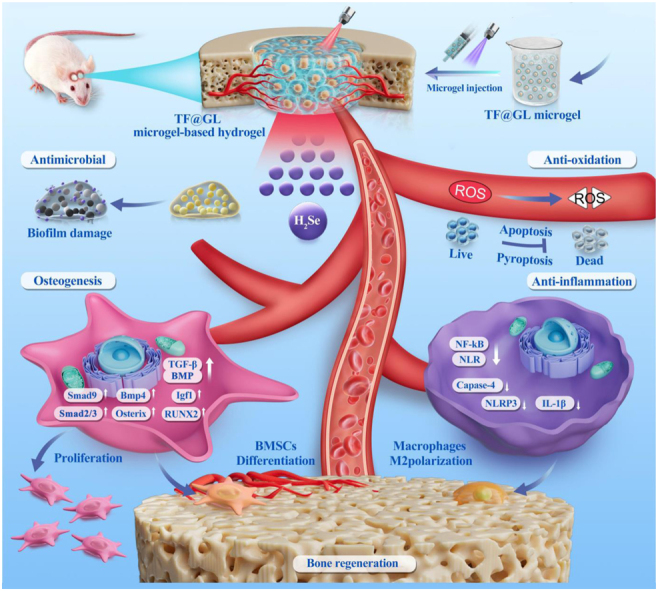

## 1 Introduction

Currently, there are approximately 20 million people worldwide suffering from diabetic ulcers, with an annual increase of 1 to 2 million patients developing diabetic osteomyelitis, posing a considerable economic burden and amputation risk [[1], [2], [3]]. The pathogenesis of diabetic osteomyelitis is influenced by both hyperglycemia and microbial infection. Oxidative stress induced by hyperglycemic state not only damages cellular activity but also weakens the immune system, creating a risk of impaired tissue repair [4,5]. Particularly concerning is the fact that hyperglycemia creates a breeding ground for bacterial proliferation, making diabetic patients more susceptible to severe infections [6,7]. Once bacteria penetrate through the cortex and muscle into the bone, it leads to bone destruction and tissue necrosis, resulting in osteomyelitis [8,9]. Therefore, it is crucial to explore a multifunctional strategy to address complex infections in clinical setting.

The prognosis of diabetic osteomyelitis hinges on addressing infections and correcting microenvironment disturbances [10]. To manage the infectious microenvironment, killing bacteria is of utmost importance [11]. The uncontrolled proliferation of bacterial biofilms creates a barrier that isolates the infection from the outside, complicating further treatment [12]. Infections are often located in the distal limbs, where diabetic-induced vascular damage and extracellular matrix (ECM) degradation significantly reduce the regenerative and repair capacity of bone tissue, leading to bone loss and destruction [13]. Thus, it is essential to focus not only on thorough short-term antibacterial measures but also on efficient bone repair following infection control.

For microbial infections that are difficult to manage in diabetic lesions, antibiotic therapy is the primary antibacterial approach in current clinical practice [14]. However, the excessive and indiscriminate use of conventional antibiotics continues to fuel the emergence and evolution of resistant bacteria, a problem that is exacerbated by diabetes [15]. Gas therapy is an emerging therapeutic paradigm that is being widely studied due to its negligible side effects [16,17]. Selenium hydride (H_2_Se) is a bioactive gas molecule that may represent the fourth gasotransmitter after nitric oxide (NO), carbon monoxide (CO), and hydrogen sulfide (H_2_S), and has garnered attention in biomedical research recently [18]. The production of H_2_Se in the body primarily relies on the metabolism of selenium (Se). H_2_Se can interfere with bacterial energy metabolism by upregulating the activity of key sulfur metabolic enzymes (e.g. sulfur oxidoreductase), thereby exerting its antibacterial effects [19]. This non-antibiotic treatment represents an urgently needed antibacterial tool, especially in the face of complex infections and the emergence of multidrug-resistant bacteria. Additionally, diabetes induces excessive ROS production, which impaires immune cell function, particularly suppressing neutrophil recruitment and phagocytic activity. Owing to its reducibility, H_2_Se can efficiently scavenge excess ROS, thereby protecting cells from oxidative damage and aiding the restoration of cellular function [20]. Moreover, ROS also impair healing by directly damaging repair-relevant cells (e.g., osteoblasts), leading to failed regeneration. While H_2_Se promotes wound healing and Se-associated proteins (e.g., sodium selenite) enhance bone density in non-diabetic models, these effects support its evaluation in diabetic osteomyelitis [18,21]. Given its antioxidant and potential osteogenic properties, H_2_Se may break the vicious cycle of chronic tissue repair failure. Therefore, exogenous H_2_Se delivery offers a promising strategy to restore microenvironmental homeostasis and accelerate the treatment of diabetic osteomyelitis.

Fe_3_O_4_ nanoparticles (NPs), which integrate low toxicity, photothermal and drug delivery capabilities, are ideal candidate materials for developing gasotransmitters [22,23]. As near-infrared (NIR) light-mediated photothermal agents, Fe_3_O_4_ NPs exhibit high photothermal conversion efficiency, enabling them to generate localized heat when subjected to specific wavelengths of light, making them suitable for photothermal therapy (PTT) [24,25]. However, when applying PTT, it is essential to find a balance between the effective antibacterial temperature and the temperature tolerated by cells. Typically, a temperature range of 50 °C to 60 °C can efficiently scavenge bacteria, but this temperature exceeds the upper tolerance limit for cells [26,27]. Therefore, precise temperature control is required to balance efficacy and safety. It is worth noting that while the antibacterial efficacy of mild PPT (MPTT, around 45 °C) is limited, short-term use can have a positive influence on osteogenic expression, promoting the proliferation and differentiation of osteoblasts, as well as supporting immune modulation and angiogenesis [28,29]. Given drug loading capacity and NIR responsiveness, Fe_3_O_4_ NPs have been selected as delivery mediums for mild photothermal-sensitive drug release.

Traditional clinical approaches to bone defect repair have limitations, including secondary injury to autologous bone donor sites and immune rejection of allogeneic bone [30]. Therefore, tissue engineering strategies are widely explored to leverage the intrinsic regenerative potential of biomaterials for bone reconstruction, actively guiding and modulating cellular behavior to achieve functional bone regeneration [31,32]. While traditional bulk hydrogels are utilized for repairing diabetic tissue defects and irregular infection sites due to their high water-content, good biocompatibility, and adjustable structure, they lack interconnected porous architectures, which severely restricts the diffusion distances of drugs and nutrients as well as cellular functionality [33,34]. To overcome the limitations of traditional bulk hydrogels in cell permeability and drug delivery, microgels are emerging as a new building unit for hydrogels. Microgels, which are cross-linked polymer particles ranging from approximately 1 to 1000 μm in size, effectively address these challenges with their excellent injectability, high mechanical strength, and responsiveness to oxidative stress (e.g., ROS) [35]. They can form porous scaffolds in vivo, significantly promoting cell migration and nutrient diffusion while enabling the controlled release of drugs and growth factors. Additionally, microgels can effectively evade clearance by the vascular and lymphatic systems, providing a robust protective barrier for the drugs and ultimately achieving sustained and controllable drug delivery effects [36]. Building on this, incorporating photothermal nanoparticles into the microgel-based hydrogel not only preserves its porous architecture but also enables dynamic, light-triggered modulation of drug release, further enhancing therapeutic precision and efficacy [37].

**Scheme 1:**
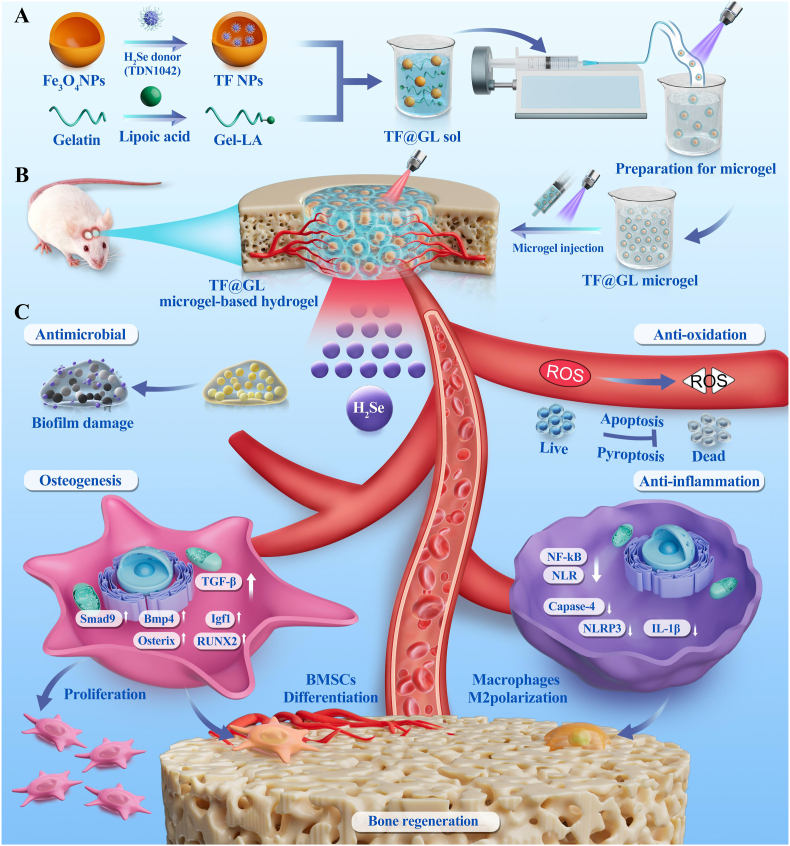
Mild photothermal-responsive hydrogel with H_2_Se delivery for anti-infection and immuno-bone regeneration in diabetic osteomyelitis. (A) Preparation of the TF@GL microgel. (B) Establishment of diabetic cranial osteomyelitis, and in situ formation of TF@GL microgel-based hydrogel for treatment. (C) The mechanism of TF@GL in antibacterial, anti-oxidation, anti-inflammation and osteogenesis to immuno-bone regeneration by the release of H_2_Se.

Here, we developed a microgel-based hydrogel for infection and anti-inflammation while promoting bone regeneration in diabetic osteomyelitis defects (Scheme 1A). To achieve H_2_Se gas delivery microgels, the H_2_Se donor TDN1042 was synthesized and encapsulated within Fe_3_O_4_ to obtain TDN1042-Fe_3_O_4_ (denoted as TF) NPs. Through an amide reaction, lipoic acid was copolymerized with gelatin molecules, resulting in gelatin-lipoic acid (denoted as Gel-LA). This endows it with photopolymerization properties for gelation. Subsequently, TF NPs were mixed with Gel-LA to prepare TDN1042-Fe_3_O_4_@Gel-LA (denoted as TF@GL) microgels using microfluidic technology. As shown in Scheme 1B, the in situ injection of TF@GL microgels at the lesion site, followed by further photopolymerization reaction, results in the formation of a microgel-based TF@GL hydrogel. Based on the photothermal-triggered properties of TF NPs, the TF@GL hydrogel enables controlled delivery of H_2_Se gas (Scheme 1C). The combination of H_2_Se with mild photothermal effects effectively combats MRSA infections and disrupts biofilms. Meanwhile, the sustained release of H_2_Se efficiently eliminates ROS in the diabetic microenvironment, suppressing ROS-mediated cell death. Furthermore, this hydrogel alleviates local inflammation by suppressing the NF-κB and NLRP3 pathways, which creates a conducive setting for subsequent activation of the TGF-β/BMP pathway and promotion of osteogenic differentiation. In conclusion, this photosensitive H_2_Se gas delivery system offers a novel strategy for the treatment of diabetic osteomyelitis.

## 2 Materials and methods

### 2.1 Materials

Gelatin and carbonyldiimidazole (CDI) was obtained from the Sigma-Aldrich (Merck KGaA, Darmstadt, Germany), FeCl_3_·6H_2_O, urea, sodium citrate, sodium polyacrylate, gelatin, Lipoic acid, Woolins' reagent, anhydrous dichloromethane, morpholine, Dimethylformamide (DMF), and dimethyl sulfoxide (DMSO) were purchased from Shanghai Macklin Biochemical Co., Ltd. (Shanghai, China), Sodium selenite (Na_2_SeO_3_) and selenium (Se) powder were obtained from Shanghai Aladdin Bio-Chem Technology Co., Ltd. (Shanghai, China). MC3T3-E1, HUVECs and RAW264.7 cells were obtained from the Chinese Academy of Science (Shanghai, China). The BMSCs were extracted from the bone marrow cavity of Sprague Dawley rats (4 weeks). Male Sprague Dawley rats were purchased from Hubei Bainter Biotechnology Co. (Wuhan, China). All of the animal experiments performed in this research were approved by the Ethics committee of Zhongnan Hospital of Wuhan University, Wuhan, China (ZN2024153). Minimum essential medium α (MEMα), Dulbecco's modified Eagle's medium (DMEM), specified endothelial cell medium (ECM), penicillin and streptomycin, fetal bovine serum (FBS), PBS, CCK8 kit, Calcein-AM/PI staining kit were obtained from Thermo Fisher Scientific (Waltham, MA, USA). Growth Factor Reduced Matrigel was obtained from Corning Life Sciences (Corning, NY, USA). JC-1, DCFH-DA, DAF-FM DA, FITC-phalloidin and DAPI were purchased from Solarbio (Beijing Solarbio Science and Technology Co.,Ltd, Beijing, China). TNF-α, IL-1β, IL-4, IL-6, IL-10, type I collagen (Col-1), osteoprotegerin (OPN) and osteocalcin (OCN) ELISA kits were sourced from Elabscience Biotechnology Co.Ltd. (Wuhan, China). Antibodies, True-Nuclear Transcription Factor Buffer Set, and Cyto-Fast Fix/Perm Buffer Set used for flow cytometry were bought from BioLegend (San Diego, CA, USA), if not specially indicated. All other standard reagents and experimental consumables were purchased from commercial suppliers (e.g., Ponsure Biological (Wuhan, China)) and used without further purification.

### 2.2 Synthesis of Fe_3_O_4_ nanoparticles (NPs)

First, 5.41 g FeCl_3_·6H_2_O, 3.61 g urea, 11.75 g sodium citrate, and 3.00 g sodium polyacrylate were added to a beaker containing 400 mL water. The mixture was stirred at 50 °C for 2 h until completely dissolved, resulting in a yellow-green transparent solution. The transparent solution was then poured into a polytetrafluoroethylene vessel, which was sealed tightly and placed in an oven at 200 °C for 12 h. After allowing the reaction vessel to cool to room temperature, the product was poured out. Any remaining product could be rinsed with ethanol, and the nanoparticles were collected using a magnet. After alternating washes with ethanol and deionized water three times, the Fe_3_O_4_ NPs were obtained by vacuum drying. The crystallinity of Fe_3_O_4_ NPs was analyzed by XRD (Empyrean, Beijing, China).

### 2.3 Synthesis of TDN1042 (H_2_Se donor)

The synthesis of the H_2_Se donor was performed according to previous work [38]. Briefly, 0.54 g Woolins reagent was weighed into a dry one-neck flask, and a stir bar was added before flushing the flask with argon. Then, 10 mL anhydrous dichloromethane and 0.44 mL morpholine were added, and the mixture was stirred at room temperature for 5 h. Upon completion of the reaction, the black precipitate was filtered out using a fritted funnel, and the resulting golden filtrate was concentrated under reduced pressure to 10% of its initial volume. The mixture was left at 0 °C overnight to promote crystallization. The crystal precipitate was collected by suction filtration, and 3 mL dichloromethane was added to wash the precipitate. Lastly, after drying under reduced pressure, a white microcrystalline solid TDN1042 was obtained. It was confirmed using ^1^H and ^31^P NMR spectroscopy, while ultraviolet absorbance spectroscopy verified the decomposition of the H_2_Se donor and the release of H_2_Se.

### 2.4 Preparation of TF NPs

20 mg Fe_3_O_4_ NPs were sonicated in 2 mL DMF for 10 min to achieve a uniform dispersion. Next, 10 mg TDN1042 were added, and the mixture was sonicated for 30 min, followed by stirring at room temperature for 24 h. Subsequently, DMF was removed via rotary evaporation, and the mixture was washed three times with deionized water to remove excess TDN1042. After vacuum drying, the TDN1042-Fe_3_O_4_ (TF) NPs loaded with H_2_Se donors were obtained.

### 2.5 Preparation of Gel-LA

To synthesize gelatin-lipoic acid (denoted as Gel-LA), Lipoic acid (0.40 g), CDI (0.35 g), and DMSO (20 mL) were added to a single-neck flask and stirred for 2 h. Meanwhile, gelatin (5.00 g) was dissolved in 50 mL water by heating it to 40 °C. The CDI-activated Lipoic acid solution was then added to the gelatin solution. The mixture was allowed to react at 35 °C for 24 h. After the reaction, the resulting solution was transferred into a dialysis bag and dialyzed against water for 3 days. Following dialysis, the Gel-LA product was freeze-dried and stored at −20 °C for use.

### 2.6 Preparation of TF@GL microgel

A 10% (w/v) aqueous solution of Gel-LA was prepared, and TF NPs dispersion was thoroughly mixed. This mixture was then loaded into a syringe and injected at a rate of 1 mL h^−1^ into a silicone tube filled with flowing ice-cold paraffin, all while being exposed to NIR irradiation to prevent premature solidification. Next, the TDN1042-Fe_3_O_4_@Gel-LA (TF@GL) microgels were solidified by exposure to 365 nm UV light (10 mW cm^−2^) for 10 s at the tube outlet. The resulting microgels were subsequently washed with n-hexane and water, followed by centrifugation to eliminate paraffin residues. The fluorescence images of the microgels dyed with rhodamine B were observed by an inverted fluorescence microscope.

### 2.7 Preparation of TF@GL hydrogel

The TF@GL microgel-based hydrogel (denoted as TF@GL hydrogel) was obtained by exposing the TF@GL microgels to 365 nm UV light (10 mW cm^−2^) for 60 s.

### 2.8 Characterization

The change in potential of TF NPs were determined by zeta potential (Zetasizer Nano ZS90, Netherlands), and the morphologies, particle sizes, and elementary compositions were performed by SEM (JSM-IT300, Japan).

The surface morphologies of GL and TF@GL hydrogels were characterized by SEM. The phase transition property of GL and TF@GL hydrogels were evaluated using a rheometer (MCR92, Anton Paar, Austria). The hydrogel precursor solution was placed in the sample stage and exposed to UV light to form a hydrogel. Subsequently, real-time changes in the storage modulus (G′) and loss modulus (G″) were recorded. A frequency scan (0.01-100 rad s^−1^; 1% strain) and a strain scan (0.01-10000% strain; 10 rad s^−1^) were then conducted to assess the rheological property. The compressive properties of GL and TF@GL hydrogel (size: 10 mm × 10 mm × 5 mm), were assessed using an electronic universal testing machine (Instron 5967, USA) at a compression rate of 1 mm min^−1^. The TF@GL hydrogel of identical dimensions were immersed in 10 times their volume of PBS, PBS+50 μм H_2_O_2_, and PBS+200 μм H_2_O_2_ solutions with Collagenasetype I (0.05 U mL^−1^), respectively. These samples were placed in a constant temperature shaker (37 °C, 100 rpm). Samples were collected, freeze-dried, and weighed every two days up to day 18 to monitor their mass loss over time.

1 mL DPPH ethanol solution (0.1 mм) was combined with equal volumes of Gel-MA hydrogel (without microgel), Gel-MA microgel-based hydrogel, Gel-LA hydrogel (without microgel), Gel-LA microgel-based hydrogel. After incubating the mixtures in the dark for 60 min, the absorbance of the supernatant was measured at 517 nm using spectrophotometer. The DPPH scavenging rate was calculated using the following formula: DPPH scavenging rate (%) = (initial absorbance - final absorbance/initial absorbance) × 100.

### 2.9 Extracellular release of TDN1042 from TF NPs

Using the UV absorption of H_2_Se donor TDN1042 at a wavelength of 280 nm in aqueous solution, the NIR-controlledrelease of TDN1042 was detected. The TF NPs were sonicated and dispersed in PBS buffer, and the UV absorption of the supernatants from the TF and TF + NIR group was measured at different time points (0.5, 1, 2, 6, 12, and 24 h).

### 2.10 Extracellular release of H_2_Se from TF NPs

The quantitative analysis of H_2_Se release was conducted using an H_2_Se gas detector (SGA-600, SINGOAN, China), which actively inhaled the gas (50 mm^3^) for real-time evaluation. The negative control was Se, while the positive control was created by mixing Na_2_SeO_3_ with glutathione (GSH) to produce H_2_Se. The concentration of released H_2_Se from TF NPs was measured by the detector at 37 °C and NIR (808 nm, 1.2 W cm^−2^, 5 min), with different pH (pH 5, 6, 7). In addition, the concentration of released H_2_Se from TF NPs was measured by the detector at 37 °C and pH 5, with NIR (808 nm, 1.2 W cm^−2^; 0, 1, 5, 10 min). The total amount of H_2_Se released within 30 min was measured.

### 2.11 Cell imaging of thermal-induced H_2_Se release from TF@GL hydrogel

To verify the release of H_2_Se from the TF NPs through thermal synergy, the H_2_Se probe (NIR-H_2_Se) was synthesized using previously established methods [39], and it was utilized to characterize the release of H_2_Se within cells. RAW 264.7 and MC3T3-E1 cells were cultured to 80% confluency in 24-well plates according to previously described methods. The different hydrogels (size: 10 mm × 10 mm × 2 mm) and H_2_Se probe (5 μм) were gently transformed into the plate with fetal bovine serum (FBS)-free DMEM medium for 60 min (37 °C, in the dark). Subsequently, medium containing the probe was removed, and cells were incubated with NIR (808 nm, 1.2 W cm^−2^, 5 min) for different durations (0, 1, 3, and 5 min) in FBS-free DMEM medium for 30 min. After washing with PBS, cells were imaged using an inverted fluorescence microscope. The fluorescence of H_2_Se was detected by an inverted fluorescence microscope (ICX41, SOPTOP, China).

### 2.12 Antibacterial activity assay

*E. coli* and MRSA were amplified using LB broth. Different hydrogels (GL, F@GL, TF@GL, F@GL + NIR, and TF@GL + NIR (NIR, 808 nm, 1.2 W cm^−2^, 5 min)), 10 μL bacterial solution (1 × 10^8^ CFU mL^−1^) and 1 mL PBS were added to a 24-well plate for 6 h at 37 °C. For the Control group, an equivalent volume of PBS was administered. Next, the hydrogels were removed, and the suspended bacteria were thoroughly mixed. Subsequently, the bacterial resuspension was diluted to 10^4^, and 10 μL of this dilution was evenly spread onto an LB agar plate. After incubating the LB agar plates at 37 °C for 24 h, the number of colony-forming units (CFU) was counted. The antibacterial activity was expressed using the following formula: Antibacterial rate (%) = ((Control CFU - Specific ager plate CFU)/Control CFU) × 100.

### 2.13 Lytic kinetics of bacteria

Bacterial growth was monitored by measuring optical density at 600 nm (OD600) in a microplate reader (FC, ThermoFisher, USA). Bacterial solution (*E. coli* or MRSA) were grown in 30 mL LB liquid medium at 37 °C, starting with an OD600 of 0.01 and shaken continuously. Once the culture reached an OD600 of 0.1, different hydrogels (GL, F@GL, TF@GL, F@GL + NIR, and TF@GL + NIR) were added to bacterial solution. Growth was monitored by OD600 every 2 h until the stationary phase.

### 2.14 Live/dead bacteria staining

The bacterial solution (*E. coli* or MRSA) was diluted to a concentration of 1 × 10^8^ CFU mL^−1^. Next, 1 mL the bacterial suspension was transferred to a sterile centrifuge tube. Different hydrogels (GL, F@GL, TF@GL, F@GL + NIR, and TF@GL + NIR) were added to the tube, while an equivalent volume of PBS was added to the Control group. The centrifuge tubes were incubated in a bacterial shaker at 37 °C for 10 h. Subsequently, 100 μL the bacterial suspension was pipetted and mixed with SYTO9 (12 μм) and PI (60 μм) for 30 min at room temperature. Finally, 5 μL the mixture was placed between a microscope slide and coverslip, and observed using an inverted fluorescence microscope.

### 2.15 Biofilm clearance assessment

Coverslips were placed in 6-well plates, where 200 μL bacterial suspension (*E. coli* or MRSA, 1 × 10^8^ CFU mL^−1^) and 2 mL fresh LB medium were added, followed by incubation for 2 days to allow biofilm formation. GL, F@GL, TF@GL, F@GL + NIR, and TF@GL + NIR hydrogels (size: 20 mm × 20 mm × 2 mm) were gently added to treat the biofilms for 24 h, while the control group received an equivalent volume of PBS. Next, the coverslips were washed with PBS and stained with crystal violet solution (0.1%) at 37 °C for 30 min. Dried coverslips were photographed after rinsing with PBS. Subsequently, the biofilm was dissolved in 95% ethanol and the optical density (OD) at 560 nm was measured using a microplate reader (Multiskan FC, ThermoFisher, USA). The percentage of biofilm clearance was calculated using the formula: Biofilm clearance (%) = ((OD Control group - OD Experimental group)/OD Control group) × 100.

### 2.16 Microscopic observation of biofilms

Bacterial biofilms were prepared and treated as described above. The biofilms were labeled with SYTO9 (green fluorescence). The coverslips containing the biofilms were removed and placed on a slide for sealing. The surface and lateral thickness of the biofilms were then observed using laser confocal microscope.

### 2.17 Detection of intracellular ROS scavenging capability

Intracellular ROS levels were assessed using the DCFH-DA (CA1410) staining. MC3T3-E1 cells were cultured with GM, GL, TF@GL, and TF@GL + NIR hydrogels in FBS-free MEMα medium for 12 h under H_2_O_2_ (100 μм or 400 μм) conditions. After H_2_O_2_ removal, the cells were incubated with DCFH-DA (10 μм) in FBS-free MEMα medium for 30 min at 37 °C in the dark. Following washing with PBS, intracellular ROS levels were visualized using an inverted fluorescence microscope, and fluorescence intensity was quantified using ImageJ software.

### 2.18 Flow cytometry

For intracellular staining, cells were blocked with a solution of PBS containing 5% BSA. (a) MC3T3-E1 cells were incubated with DCFH-DA (1 h, 4 °C in the dark). (b) MC3T3-E1 cells were treated with Annexin V and PI (1 h, 4 °C in the dark). (c) BMSCs were incubated with JC-1 (1 h, 4 °C in the dark). (d) RAW264.7 macrophages were stained with PerCP/Cyanine anti-mouse CD86 antibody (105005), APC anti-mouse CD206 (MMR) antibody, FITC anti-mouse/human CD11b antibody (101205), and PE anti-mouse F4/80 antibody (123109) (1 h, 4 °C in the dark). (e) Macrophages were harvested and digested from bone defect region and surrounding tissues to obtain a single-cell suspension, followed by the previously described macrophage staining procedures. The cells were then washed with PBS and analyzed via flow cytometry (BD FACSCelesta, USA). Results were processed with FlowJo software.

### 2.19 Live/dead cell staining

After MC3T3-E1 cells reached 80% confluence, cells were seeded onto the surface of GM, GL, TF@GL, and TF@GL + NIR hydrogels in FBS-free MEMα medium for 12 h, in the presence of H_2_O_2_ (400 μм). After H_2_O_2_ removal, the live/dead cell labelling kit (C2015M) containing PI and Calcein-AM was used to stain MC3T3-E1 cells for 60 min at 37 °C in the dark. Following PBS washing, fluorescence images were obtained using an inverted fluorescence microscope. To assess cell proliferation, BMSCs were cultured with different hydrogels in the absence of H_2_O_2_, followed by staining as described previously.

### 2.20 Mitochondrial transmembrane potential measurement

Mitochondrial membrane potential was evaluated by monitoring JC-1 fluorescence aggregates (CA1310-100). BMSCs were cultured with GM, GL, TF@GL, and TF@GL + NIR hydrogels in FBS-free MEMα medium for 12 h in the presence of H_2_O_2_ (400 μм). After H_2_O_2_ removal, cells were incubated with JC-1 (2.5 μg mL^−1^) in FBS-free MEMα medium for 30 min at 37 °C in the dark. Subsequent to PBS washing, JC-1 aggregates and monomers were visualized with an inverted fluorescence microscope.

### 2.21 Western blotting analysis

After processing the cells as described above, BMSCs were lysed for 30 min at 0 °C in RIPA buffer (Yami, PC101, China) supplemented with phosphatase inhibitors. Total protein concentrations were determined using the BCA protein assay (Beyotime, P0012). For the Western blot, 40 μg of protein was separated by SDS-PAGE and transferred to PVDF membranes. The membranes were blocked with 5% non-fat milk, followed by overnight incubation at 4 °C with primary antibodies (Bax, Bcl-2, Caspase-3, NLRP3, N-GSDMD, and Caspase-1). After washing, the membranes were incubated with secondary antibodies for 30 min at room temperature in the dark. Protein bands were detected using a chemiluminescence system with enhanced chemiluminescence reagents, with GAPDH as the loading control. Finally, band intensity was quantified using ImageJ software (National Institutes of Health, USA).

### 2.22 Quantitative real-time polymerase chain reaction (qRT-PCR)

Total RNA was extracted and reverse transcribed into complementary DNA using the PrimeScript RT Master Mix. RT-PCR analysis was performed with a Bio-Rad RT-PCR system to assess the expression levels of various genes, including NLRP3, N-GSDMD, Caspase-1, BAX, BCL2, Caspase-3, Smad9, Bmp4, Igfl, Osterix, RUNX2 (from BMSCs), IκKα, IκKβ, NF-κB1, NLRP3, IL-1β, Caspase-4 (from RAW 264.7 cells), CD31, VEGF (from HUVECs), and Igfl, Bmp4, Osterix, RUNX2, CD31, VEGF (from rats). Glyceraldehyde-3-phosphate dehydrogenase (GAPDH) served as the internal control. Primer sequences for the assessed genes are provided in Tables S2, S3 and S4.

### 2.23 Inflammation and polarization of macrophages

RAW 264.7 macrophages were seeded at a density of 1 × 10^6^ cells per well in 6-well plates. Once the cells reached 90% confluency, they were stimulated with LPS (100 ng mL^−1^). After 24 h, the LPS was gently washed off with PBS three times. Samples of GM, GL, TF@GL, and TF@GL + NIR hydrogels (size: 20 mm × 20 mm × 2 mm) were then introduced into the wells. The LPS group, designated as the control, received an equivalent volume of PBS. RAW 264.7 cells were co-cultured with the different samples for 48 h.

### 2.24 Immunofluorescence staining

After processing the cells as described above, RAW 264.7 cells were fixed with 4% paraformaldehyde for 20 min followed by incubation with primary antibodies against iNOS and CD206 (diluted 1:100) at 4 °C overnight. Afterward, the cells were treated for 30 min with either Alexa Fluor 488-conjugated anti-mouse or Alexa Fluor 594-conjugated anti-mouse secondary antibodies. DAPI was then applied for 60 s at room temperature in the dark to stain the nuclei. Immunofluorescence was visualized using a laser scanning confocal microscope, and fluorescence intensities for iNOS and CD206 were quantified using ImageJ software.

### 2.25 Transcriptome sequencing and data analysis

Following the processing of cells as described, macrophages were lysed with Trizol reagent (Beyotime Biotechnology), and the resulting lysates were stored at −80 °C until sequencing. RNA sequencing was conducted using an Illumina HiSeq X10 (Illumina, USA). Gene expression values were transformed using log_10_[TPM (Transcripts Per Million) + 1]. GO and KEGG pathway enrichment analyses were performed using the free online platform provided by Majorbio (www.majorbio.com). The same procedure was applied to BMSCs without requiring any additional instructions.

### 2.26 Cell proliferation assay

BMSCs (initially at 80% confluency) were cultured with GM, GL, TF@GL, and TF@GL + NIR hydrogels for 24, 48 or 72 h according to previously described methods. Subsequently, CCK-8 solution was added to react 1 h and 100 μL mixture was pipetted into a 96-well plate. A microplate reader was used to detect the OD at 450 nm. The cell proliferation was calculated in accordance with the following formula: Cell proliferation (%) = (mean of absorbance value of treatment group/mean of absorbance value of control) × 100. Additionally, after co-cultured with hydrogels for 2 days, BMSCs (initially at 20% confluency) were fixed with paraformaldehyde (4%) and then stained with FITC-Phalloidin (CA1620) and DAPI (C0065) to visualize the cytoskeletons.

### 2.27 ALP and alizarin red staining

To evaluate ALP activity and mineralization in BMSCs cultured on GM, GL, TF@GL, and TF@GL + NIR hydrogels, we performed ALP staining on day 7 and Alizarin Red staining on day 21. After the specified culture periods, the cells were fixed with 4% paraformaldehyde for 30 min. For ALP staining, a BCIP/NBT ALP Color Development Kit (C3206) was applied for 30 min at room temperature, after which the cells were washed with PBS and observed under a microscope. Similarly, for Alizarin Red staining, an Alizarin Red S Staining Kit (C0148S) was used for 30 min at room temperature, followed by washing with PBS and microscopic observation.

### 2.28 Migration assay

HUVECs were seeded at a density of 1 × 10^6^cells per well in 6-well plates. A straight scratch was created in the monolayer using a 100-μL sterile pipette tip once the HUVECs reached 90% confluency. The scraped area was gently washed with PBS. Conditioned media collected from the BMSCs culture system (B-Control, B-GM, B-GL, B-TF@GL, B-TF@GL + NIR) were added to the wells in a 1:1 ratio with HUVECs culture media. Migrated HUVECs were assessed at 0, 12, and 24 h post-scratch, labeled with Calcein-AM (green fluorescence), and imaged using an inverted fluorescence microscope. The healing ratio of the scratch area was quantified with ImageJ software. For the Transwell migration assay, hydrogels were placed in the lower chambers, and HUVECs were added to the upper chambers. After 24 h, the migrated cells were stained with crystal violet for 15 min and observed under a microscope.

### 2.29 Matrigel tube formation assay

Matrigel matrix (200 μL per well) was added to coat 24-well plates prior to seeding HUVECs (5 × 10^4^ cells well^−1^). Conditioned media collected from the BMSCs culture system (B-Control, B-GM, B-GL, B-TF@GL, B-TF@GL + NIR) were added to the wells in a 1:1 ratio with HUVECs culture media. After 6 h, HUVECs were labeled with Calcein-AM (green fluorescence) and observed for tube formation using an inverted fluorescence microscope. Tube formation parameters were analyzed using ImageJ software.

### 2.30 Establishment of cranial osteomyelitis of type 2 diabetes rat model

The Institutional Animal Care and Utilization Committee (IACUC) of the Animal Experiment Center of Wuhan University (Approval number: WP20230642) approved all animal experiments, which were conducted in accordance with the National Research Council's Guidelines for the Care and Use of Experimental Animals. SD rats were continuously maintained on a high-fat and high-sugar diet for 4 weeks, followed by an intraperitoneal injection of streptozotocin (STZ, 25 mg kg^−1^) dissolved in citrate buffer (pH 4.5). Blood glucose levels were recorded. Two weeks post-STZ injection, rats with blood glucose levels exceeding 16.8 mmol L^−1^ were classified as diabetic.

Following successful anesthesia, the crania were shaved and disinfected before making a midline incision. A sagittal cut of approximately 1.5 cm was made on the scalp for exposure, and careful blunt dissection was performed to reveal the cranium. Two critical size defects, each with a diameter of 5.0 mm, were created symmetrically on both sides of the cranium using a motorized trephine (Nouvag AG, Goldach, Switzerland), ensuring that brain tissue was not damaged during the procedure. Each defect was filled with a gelatin sponge incorporating a bacterial solution (MRSA, 10^7^ CFU mL^−1^) for establishment osteomyelitis model, and the incisions were subsequently closed using 6-0 medical sutures. 2 weeks after the surgery for the osteomyelitis model, debridement was performed on the infected defects. Subsequently, different microgels were used to fill the defects, and cross-linking occurred to form hydrogels after 60 s UV. The incisions were then sutured with 6-0 surgical sutures.

### 2.31 Antibacterial effect in vivo

On week 1 after the hydrogel treatment, the rats were euthanized. Granulation tissue from the defect site was harvested to assess the antibacterial effect using the spread plate method. The cranial specimens were fixed in 4% formaldehyde, followed by immersion in EDTA for decalcification, and subsequently stained with Giemsa.

### 2.32 Micro-CT

On week 4 and week 8 after the hydrogel treatment, the rats were euthanized. The cranial specimens obtained were fixed and scanned using a micro-CT system (SkyScan 1276, Bruker). NRecon, CTVol, CTAn, and CTVox software were used for reconstructing 3D models and statistical analyzing indicators of bone formation.

### 2.33 Pathological staining

The cranial specimens were performed as previously. The bone defect region were subjected to histopathological staining using H&E, Masson's reagent, as well as immunohistochemistry (Igf1, Bmp4, Osterix, RUNX2) on week 4 after the microgel treatment and immunofluorescence (ROS, NF-κB, TNF-α, IL-10, CD31, VEGF) on week 4 after the hydrogel treatment. The fluorescence intensities were quantified by ImageJ software.

### 2.34 Statistical analysis

Each experiment was evaluated using the mean values ± standard deviation (SD) from at least three independent biological replicates (n ≥ 3). The researchers performing the interventions and analyzing the outcomes were blinded to the group assignments to minimize bias. Data analysis was performed using GraphPad Prism version 10 and Origin Software (Version 2019). One-way analysis of variance (ANOVA) was used to identify significant differences unless otherwise stated. The *p* value < 0.05 was considered to indicate statistically significant results. **∗***P* < 0.05, **∗∗***P* < 0.01, **∗∗∗***P* < 0.001, **∗∗∗∗***P* < 0.0001, ns: not significant.

## 3 Results and discussion

### 3.1 Synthesis and characterization of TF nanoparticles

**Fig. 1:**
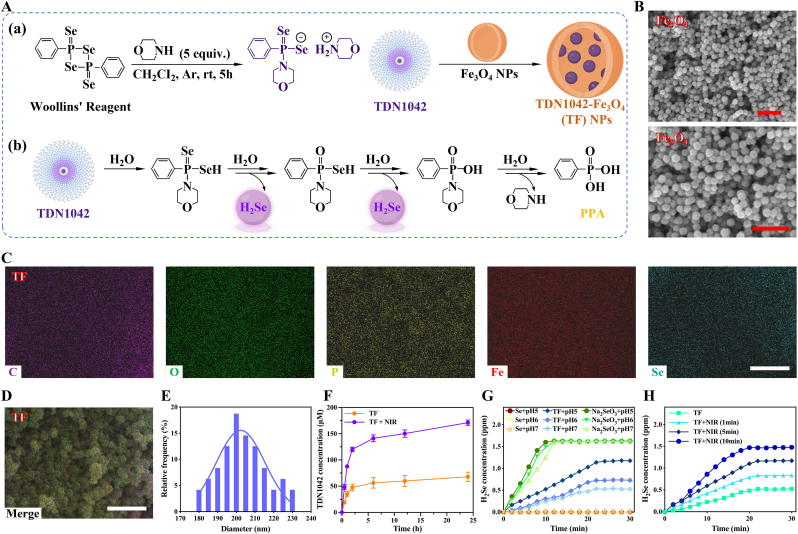
Characterization of TF NPs. (A) Schematic diagram of (a) the synthetic TF NPs and (b) hydrolysis mechanism of TDN1042 resulting in H_2_Se release. (B) SEM images of Fe_3_O_4_ NPs. Scale bars: 1.0 μm. (C) Split images and (D) merged image of elemental mapping of TF NPs. Scale bars: 1.0 μm. (E) Diameter statistical histogram and normal distribution curve of TF NPs. (F) Release curve of TDN1042 from TF NPs with or without NIR. (G) Quantitative analysis of H_2_Se concentration at different pH, with NIR (808 nm, 1.2 W cm^−2^, 5 min). Na_2_SeO_3_ group mixed with GSH to generate H_2_Se. (H) Quantitative analysis of H_2_Se concentration at pH 5.0, with different durations of NIR (808 nm, 1.2 W cm^−2^).

As illustrated in Fig. 1A, the preparation process of TDN1042-Fe_3_O_4_ nanoparticles (denoted as TF NPs) and the release of H_2_Se from H_2_Se donor TDN1042. Initially, the Fe_3_O_4_ NPs were synthesized, and the cubic crystal structure was verified using X-ray diffraction (XRD) pattern (Fig. S1), while the morphology was obtained from scanning electron microscopy (SEM) images (Fig. 1B). Next, Woolins and morpholine were reacted to form H_2_Se donor TDN1042. which was confirmed by the ^1^H and ^31^P NMR spectra (Fig. S2). Subsequently, TDN1042 was encapsulated in Fe_3_O_4_ NPs to obtain TF NPs. The elemental mapping and energy dispersive spectrum (EDS) confirmed the uniform distribution of C, O, P, Fe and Se in TF NPs, indicating the successful encapsulation of H_2_Se donor (Fig. 1C and D, S3). Besides, the zeta potential showed an increase in negative charge of the TF NPs after encapsulation of TDN1042, which contributed to improved dispersion of the nanoparticles (Fig. S4). The average diameter of the TF NPs was measured at 202.30 ± 12.97 nm (Fig. 1E).

Furthermore, the release of H_2_Se donor and H_2_Se from TF NPs were investigated. To assess the effects of pH and temperature on TDN1042 hydrolysis, the rate of hydrolysis (10 mм) was measured with or without NIR exposure at pH 5.0, 6.0, and 7.0. As shown in Fig. S5, the hydrolysis of TDN1042 was accelerated under mild photothermal conditions. Additionally, the hydrolysis rate of TDN1042 increased at more acidic pH levels. It indicated that the H_2_Se donor accelerated the production of H_2_Se under NIR and acidic conditions. Besides, the thermal controlled release capability of H_2_Se donor loaded onto TF NPs was investigated. As shown in Fig. 1F, TDN1042 was released slowly in the absence of NIR. However, with the application of NIR, the donor was able to rapidly release into the solution. This demonstrated that the application of NIR accelerated the release of H_2_Se donor from TF NPs and expedited H_2_Se release. In addition, the release of H_2_Se was quantitatively assessed. Na_2_SeO_3_ could be reduced to H_2_Se by glutathione, while Se powder released almost no H_2_Se in water; these served as positive and negative controls, respectively. At pH 7, the release of H_2_Se from the TF group increased over time, reaching a plateau (0.53 ppm) within 22 min, whereas the H_2_Se in the Na_2_SeO_3_ group reached a plateau (1.62 ppm) after 12 min, exhibiting the fastest release rate (Fig. 1G). At pH 5, the TF group released 1.18 ppm H_2_Se, indicating that an acidic environment promoted its release. This pH-responsive characteristic was applicable to the acidic microenvironments in diabetes and bacterial infections for the release and application of H_2_Se. Moreover, the longer the NIR exposure, the greater the release of H_2_Se, further indicating that the release of H_2_Se from TF NPs could be partially controlled by NIR (Fig. 1H). To evaluate the dose-response of H_2_Se, BMSCs were exposed to a concentration gradient, and a therapeutic window of 1.0–3.0 μg mL^−1^ was identified (Fig. S6).

### 3.2 Preparation and characterization of TF@GL hydrogel

**Fig. 2:**
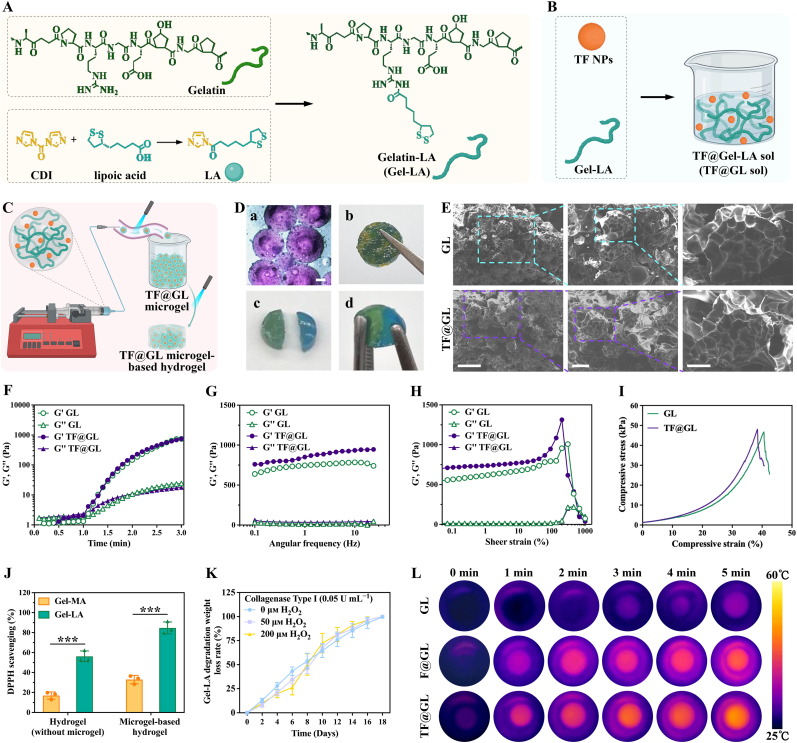
Characterization of TF@GL hydrogel. (A) Schematic diagram of the synthetic lipoic acid grafted gelatin, (B) TF@GL sol, and (C) TF@GL microgel hydrogel. (D) Photographs of (a) microgel stained with rhodamine B (Scale bars: 100 μm), (b) two colors of Gel-LA microgels forming complete hydrogels after UV irradiation, (c) healing of two Gel-LA hydrogels after UV irradiation. (E) SEM images showing the microstructures of GL and TF@GL microgel hydrogels. Scale bars: 500 μm, 200 μm, and 100 μm. (F) Relationship between sol-gel transition and photocrosslinking time, (G) Frequency scanning rheology (0.1-10 Hz, 1% strain), (H) Rotational strain scanning rheology (0.01-10000% strain, 1 Hz), (I) Stress-strain curves of GL and TF@GL microgel-based hydrogel. G': storage modulus; G": loss modulus. (J) DPPH scavenging by hydrogel and microgel-based hydrogel of Gel-MA and Gel-LA (n = 3). (K) Degradation of Gel-LA microgel-based hydrogel in PBS containing Collagenase type I and H_2_O_2_ (0, 50 μM, 200 μM) (n = 3). (L) The thermal images of hydrogels under NIR irradiation (808 nm, 1.2 W cm^−2^). Data are presented as mean ± SD; **∗∗∗***P* < 0.001.

As showed in Fig. 2A, Gelatin was modified with lipoic acid to prepare Gelatin-LA (denoted as Gel-LA), wherein the lipoic acid has a disulfide bond, so that Gel-LA can undergo disulfide bond breakage and recombination under ultraviolet light, leading to gelation. The elemental analysis results of Gelatin and Gel-LA showed that approximately 1.1 molecules of lipoic acid were grafted onto each repeating unit of Gelatin (Table S1). TF NPs was then added to Gel-LA to create TF@Gel-LA (denoted as TF@GL) solution (Fig. 2B). Next, TF@GL microgels were fabricated by TF@GL solution using microfluidic technology. Subsequently, the TF@GL microgels were added to the mold and exposed to UV light to further crosslink, resulting in a complete TF@GL microgel-based hydrogel (denoted as TF@GL hydrogel) (Fig. 2C). The TF@GL hydrogel was composed of lyophilized Gel-LA and vacuum-dried TF NPs, both of which exhibited good stability at low temperatures and could be stored long-term at −20 °C. More importantly, the precursor solutions of the microgels were mixed immediately before use, thereby avoiding the stability issues associated with pre-mixed formulations. Therefore, the system could be formulated as a lyophilized two-component that was reconstituted and mixed just prior to injection, complying with clinical aseptic handling requirements. The microfluidic fabrication process enabled uniform microgel production by precisely controlling parameters such as injection flow rate, oil phase flow rate, and UV crosslinking duration, meeting the batch-to-batch consistency standards required for medical devices and demonstrating feasibility for clinical-scale manufacturing.

Through optical microscopy, microgels stained with rhodamine B appeared uniformly spherical with a diameter of approximately 300 to 400 μm (Fig. 2D (a)). Moreover, TF@GL microgels dyed in two different colors were combined in a mold, resulting in a cohesive hydrogel after 60 s of UV exposure (Fig. 2D (b)). In addition, two stained TF@GL hydrogel were brought together, bonding effectively after 60 s of UV irradiation (Fig. 2D (c, d)). These images of microgel mixing and hydrogel self-healing demonstrated that TF@GL microgels and hydrogels still retained some cross-linking ability after initial gelation. Cyclic strain tests further demonstrated that both GL and TF@GL hydrogels exhibited self-healing capability (Fig. S7). To observe the morphology after microgel crosslinking, SEM was used to examine the freeze-dried microgel-based hydrogels. Fig. 2E illustrated that the GL and TF@GL microgel-based hydrogels were primarily composed of microgel modules, with pore sizes between the modules ranging from 200 to 400 μm. This porous structure provided suitable spaces that promoted the adhesion and migration of bone regeneration-related and supporting cells while facilitating the efficient diffusion of nutrients and oxygen into the central region of the conduit, which was crucial for nutrient supply to the cells [[40], [41], [42]]. According to the rheological properties, both GL and TF@GL hydrogels exhibited solid-like elastic behavior (storage modulus G' > loss modulus G″) after light cross-linking for 10 s, indicating the addition of TF NPs did not significantly affect the light cross-linking rate of GL (Fig. 2F). The frequency sweep results indicated that the TF@GL hydrogel maintained its state at 1% strain and shear frequencies ranging from 0.1 Hz to 10 Hz. The strain sweep results showed that the TF@GL hydrogel retained strength at a shear rate of 1 Hz and across a strain range from 0.01% to nearly 100%. This demonstrated that the TF@GL hydrogel possesses excellent elastic properties and a stable network structure, resulting in favorable mechanical characteristics. In addition, the TF@GL hydrogel maintained its state at 1% strain and shear frequencies ranging from 0.1 Hz to 10 Hz (Fig. 2G). At a shear rate of 1 Hz, it retained strength across a strain range from 0.01% to nearly 100% (Fig. 2H). The frequency sweep and strain sweep results demonstrated that the TF@GL hydrogel possessed excellent elastic properties and a stable network structure, resulting in favorable mechanical characteristics. Compression tests showed that the microgels made from lipoic acid-modified gelatin exhibit good compressive strength (Fig. 2I). Furthermore, the incorporation of TF NPs into the microgels did not significantly affect compressive performance of microgel-based hydrogel (Fig. S8A). However, the Young's modulus of TF@GL was slightly higher than GL, presumably due to the TF NPs, acting as fillers, absorbing external stress and enhancing the deformation resistance of the system (Fig. S8B).

Excessive free radicals in a diabetic microenvironment could lead to oxidative stress, damaging cell survival and tissue repair. Neutralizing free radicals could reduce damage to surrounding tissues and help accelerate the repair and healing of bone defect. The DPPH scavenging efficiency was used to assess the antioxidant capacity of the hydrogels. The Gel-LA hydrogel (without microgel) scavenged 56.17 ± 3.10% of DPPH within 1 h, while the microgel-based hydrogel scavenged rate of 84.71 ± 3.46% (Fig. 2J). This was attributed to the larger specific surface area of the microgels, which allowed for easier capture of DPPH molecules in solution. Gel-MA, due to its porous structure, also exhibited some DPPH scavenging ability, but its antioxidant capacity was significantly lower than the Gel-LA containing disulfide bonds. To reconstruct tissue within bone defect, the degradation of the hydrogel needed to be considered to avoid hindering the osteoblasts growth. In PBS with Collagenase type I (0.05 U mL^−1^), the Gel-LA hydrogel exhibited a stable degradation profile, with its mass loss of approximately 93.37 ± 5.82% by day 16 and nearly complete degradation by day 18, thereby preventing long-term physical obstruction to tissue regeneration (Fig. 2K). Even in the 50 μм and 200 μм H_2_O_2_, its degradation kinetics showed no significant changes, indicating that the hydrogel did not undergo premature collapse under oxidative conditions.

To visually analyze their photothermal capabilities, infrared thermal images and real-time temperature changes were recorded during a 5-min NIR exposure (808 nm, 1.2 W cm^−2^) (Fig. 2L). The real-time temperature change curves were consistent with the real-time infrared thermal images, showing that the GL group exhibited a temperature increase (ΔT) of 11.29 °C (Fig. S9). The F@GL and TF@GL hydrogels exhibited equivalent temperature increases, reaching 42.48 °C and 42.12 °C, respectively, which are acceptable short-term temperature ranges for cells. To further evaluate the H_2_Se release from the TF@GL hydrogel, quantitative assessments were performed under varying pH conditions and NIR irradiation durations. At pH 5, H_2_Se release from the TF@GL hydrogel increased over time and reached a plateau (0.94 ppm) within 44 min (Fig. S10A). As the pH increased, the amount of H_2_Se released gradually decreased. Additionally, longer NIR irradiation times led to greater H_2_Se release and faster attainment of the plateau, indicating that H_2_Se release from the TF@GL hydrogel was controllable (Fig. S10B). To determine whether photothermal effects in the cellular environment could trigger the accelerated release of H_2_Se gas, the accumulation of H_2_Se was evaluated using the H_2_Se fluorescent probe (NIR-H_2_Se). In MC3T3-E1 and RAW 264.7 cells, under no thermal effects (0 min), the fluorescence intensity was low, suggesting minimal release of H_2_Se in the normal cellular environment (Fig. S11). As the thermal treatment time increased, the red fluorescence intensity gradually rose, confirming the thermal synergistic controlled release of H_2_Se. This could be employed for gentle photothermal and accelerated delivery of H_2_Se in sites of infection and inflammation in vivo.

### 3.3 Anti-bacterial ability of photothermal TF@GL hydrogel

**Fig. 3:**
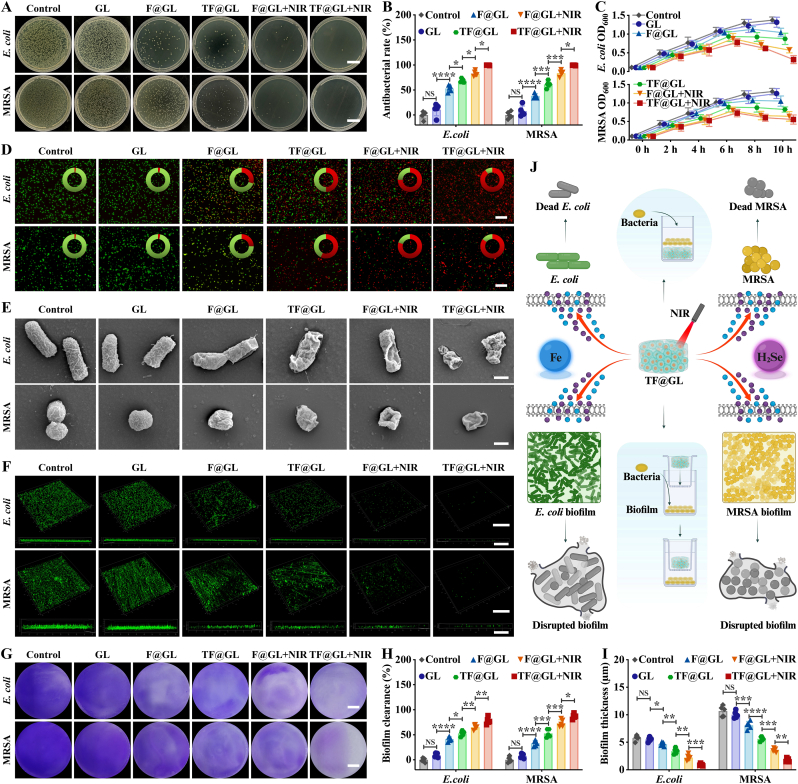
Antibacterial effect and biofilm eradication of TF@GL microgel hydrogel in vitro. (A) Spread-plate assays of *E. coli* and MRSA bacterial colonies treated with different hydrogels, with or without NIR. Scale bars: 2 cm. (B) Quantitation of bacterial colonies based on spread-plate assays (n = 5). (C) Lytic kinetics (OD_600_ value) of *E. coli* and MRSA treated with each group for 10 h (n = 3). (D) Live (SYTO9, green) and dead (PI, red) staining of *E. coli* and MRSA. Scale bars: 100 μm. (E) SEM images of *E. coli* and MRSA. Scale bars: 500 nm. (F) CLSM 3D images of the biofilm removal effect. The existence of biofilm was examined with SYTO9 dye (green). Scale bars: 50 μm, 50 μm. (G) Crystal violet staining of bacterial biofilms. Scale bars: 5 mm. (H) Quantitation of biofilm clearance based on Crystal violet staining (n = 5). (I) Quantitation of biofilm thickness based on CLSM 3D images (n = 5). (J) Schematic diagram of TF@GL hydrogel enhancing antibacterial activity. Data are presented as mean ± SD; **∗***P* < 0.05, **∗∗***P* < 0.01, **∗∗∗***P* < 0.001, **∗∗∗∗***P* < 0.0001.

Bacterial infections caused by orthopedic surgeries or osteomyelitis remain a challenging clinical problem. Therefore, two representative drug-resistant bacteria, *E. coli* and MRSA, were used to evaluate in vitro antibacterial efficacy of photothermal TF@GL hydrogel. As shown in the bacterial coating plates, the antibacterial activities of the F@GL and TF@GL hydrogels against *E. coli* were 53.32 ± 2.89% and 68.53 ± 1.67%, while against MRSA were 38.32 ± 1.97% and 61.14 ± 2.95%, respectively (Fig. 3A and B). The antibacterial rates of the F@GL and TF@GL groups increased slightly under NIR irradiation for 5 min, rising to over 82% and over 99%, indicating the antibacterial effects of PPT. Next, bacterial optical density (OD) measurements served to crudely and dynamically assess bacterial concentrations (Fig. 3C). The control group started around 0.1 and exceeded 1.0 within 6 h, then increased slowly and stabilized, consistent with the typical logarithmic and stationary phases of bacterial growth. The TF@GL + NIR group inhibited the reproduction of bacteria and caused a clear inflection point in growth curve at 6 h. This indicates that the accumulation of Fe^2+^ ions and H_2_Se killed the bacteria over time. Subsequently, the survival of bacteria co-cultured with the hydrogels for 10 h was assessed using SYTO9/PI staining (Fig. 3D). Compared to the control group, sparse red bacteria were observed in the presence of Fe^2+^ ions (F@GL), indicating a slight antibacterial effect. The release of H_2_Se gas (TF@GL) resulted in fewer live bacteria and more dead bacteria of *E. coli* and MRSA. With the addition of phototherm (TF@GL + NIR), the proportion of dead bacteria became overwhelmingly dominant. Furthermore, the SEM images revealed that *E. coli* and MRSA treated with the TF@GL + NIR group exhibited abnormal morphologies, including cell wall rupture, cell contraction, and membrane shrinkage (Fig. 3E). Meanwhile, bacteria treated with the F@GL and TF@GL groups displayed varying degrees of wrinkling and deformation. In stark contrast, untreated bacteria (control) exhibited normal morphology with intact cell membranes.

In osteomyelitis, the biofilm acts as a foundation for the bacteria, and the expanding bacterial population hinders tissue reconstruction. Damaging the established biofilm is crucial for controlling severe infections. Confocal laser scanning microscopy (CLSM) provided corroborative evidence from a microscopic perspective in the ortho-lateral position. As depicted in Fig. 3F, the bacterial biofilm in the control group was multilayered and dense, resembling a layer of armor. However, the TF@GL + NIR hydrogel exhibited the weakest green fluorescence signal (live bacteria) from and a significant reduction in biofilm thickness (Fig. 3I). From a macroscopic perspective, crystal violet staining demonstrated that the TF@GL + NIR hydrogel significantly disrupted biofilms, resulting in higher clearance than in the other five groups (Fig. 3G and H). These results were attributed to TF NPs from the TF@GL hydrogel providing a targeted localized photothermal effect. Additionally, Fe^2+^ ions could damage the bacterial membranes, leading to leakage of internal substances [43]. H_2_Se upregulated the enzymatic activity of the key sulfur metabolic enzyme SOX (sulfur oxidoreductase) (Fig. S12A), thereby interfering bacterial energy metabolism by hindering ATP synthesis (Fig. S12B), ultimately disrupting biofilm metabolic homeostasis [44]. Collectively, photothermal effects, Fe^2+^ ions and H_2_Se gas serve as three key components, providing the hydrogel system with potent broad-spectrum antibacterial activity (Fig. 3J).

### 3.4 TF@GL hydrogel induced intracellular ROS-scavenging and inhibited cell death under oxidative stress

**Fig. 4:**
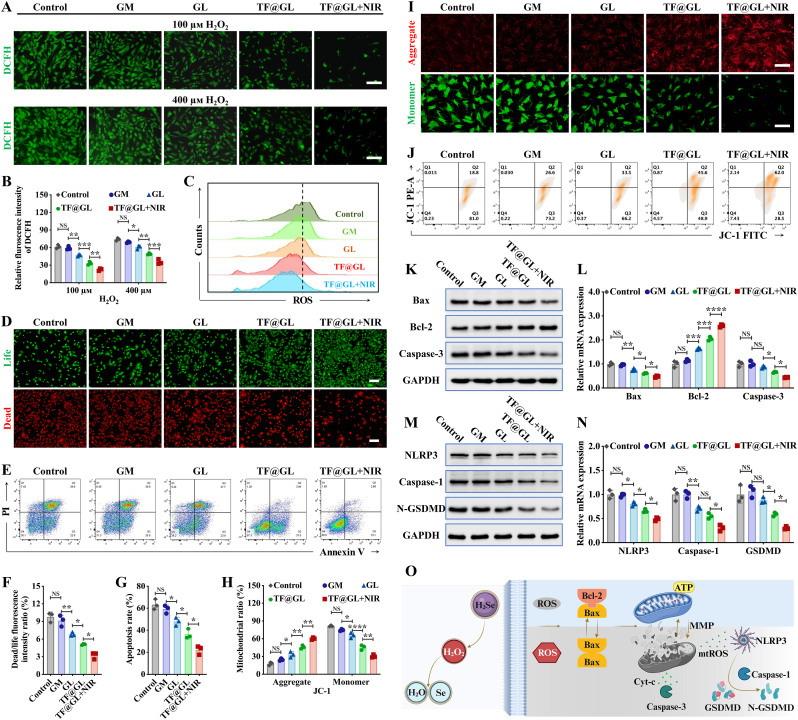
TF@GL hydrogel exerted antioxidant effect and alleviated cell death. (A) ROS staining (DCFH-DA probe, green) of MC3T3-E1 cells cultured with different hydrogels and 100 or 400 μм H_2_O_2_ after 12 h. Scale bars: 50 μm. (B) Quantitation of ROS scavenging capability of hydrogels (n = 3). (C) Representative flow cytometry plots showing ROS of MC3T3-E1 cells cultured with different hydrogels and 400 μм H_2_O_2_ after 12 h. (D) Live/dead staining of MC3T3-E1 cells adhered to the surface of different hydrogels and 100 μм H_2_O_2_ for 24 h. Scale bars: 100 μm. (E) Representative flow cytometry plots showing apoptosis of MC3T3-E1 cells cultured with different hydrogels and 400 μм H_2_O_2_ after 12 h. (F) Quantitation of the live/dead cell ratio (n = 3). (G) Quantitation of apoptotic cells including early apoptotic cells (AnnexinV^+^PI^−^) and later apoptotic cells (AnnexinV^+^PI^+^) (n = 3). (H) Quantitation of mitochondrial aggregate (JC-1 FITC^+^PE-A^+^) and monomer (JC-1 FITC^+^PE-A^−^) cells (n = 3). (I) JC-1 aggregate (red) and JC-1 monomer (green) staining of BMSCs cultured with different hydrogels and 400 μм H_2_O_2_ for 12 h. Scale bars: 50 μm. (J) Representative flow cytometry plots showing JC-1 stained BMSCs cultured with different hydrogels and 400 μм H_2_O_2_ for 12 h. (K) Western blot images of protein expression, and (L) relative mRNA expression of apoptosis genes of Bax, Bcl-2 and Caspase-3 in BMSCs treated with hydrogels and 400 μм H_2_O_2_ after 12 h (n = 3). (M) Western blot images of protein expression, and (N) relative mRNA expression of pyroptosis genes NLRP3, Caspase-1 and N-GSDMD in BMSCs (n = 3). (O) Schematic diagram of cellular ROS scavenging and maintenance of mitochondrial integrity induced by hydrogel. Data are presented as mean ± SD; **∗***P* < 0.05, **∗∗***P* < 0.01, **∗∗∗***P* < 0.001, **∗∗∗∗***P* < 0.0001.

It was demonstrated that ROS interfere with osteogenic differentiation and induce osteoblast death in diabetic microenvironment, which is a major obstacle to bone repair [5,45]. Correcting oxidative stress is essential for the bone repair process. The ROS scavenging ability of TF@GL hydrogel in Mouse calvaria-derived preosteoblast (MC3T3-E1) cells was assessed using the DCFH-DA fluorescence probe. As shown in Fig. 4A, the 400 μм H_2_O_2_ treatment induced stronger green fluorescence intensity than the 100 μм H_2_O_2_ treatment, indicating an increase in ROS production. Compared to the GM group (GelMA), the GL group showed weaker fluorescence and a significant reduction in intracellular ROS expression, primarily due to the Gel-LA containing disulfide bonds. The TF@GL + NIR group exhibited the lowest ROS levels (Fig. 4B), which may be related to the protective effect of H_2_Se gas. This trend was further confirmed by ROS flow cytometry analysis. As illustrated in Fig. 4C and S13A, treatment with TF@GL hydrogel led to a clear leftward shift in the ROS signal peak, indicating a decrease in intracellular ROS levels. The TF@GL + NIR group was clearly positioned on the far left, indicating that the release of H_2_Se was enhanced under the mild photothermal condition.

Given the gratifying antioxidant performance of the TF@GL hydrogel, its protective role in apoptosis was tested. Live/dead staining showed that after treatment with 400 μм H_2_O_2_, red cells were more prevalent in both the control and GM groups, while in the TF@GL + NIR group, green cells were more prominent (Fig. 4D and F). Additionally, the Annexin V/PI flow cytometry analysis of apoptosis rate in control, GM, GL, TF@GL, TF@GL + NIR respectively was 63.27 ± 2.69%, 59.23 ± 2.11%, 48.08 ± 2.11%, 36.36 ± 2.56%, 22.27 ± 2.28%, further indicating that the TF@GL + NIR group had the best cellular antioxidant capacity (Fig. 4E and G). Moreover, excessive oxidation can trigger endogenous apoptosis, with mitochondria being the main conduit of the intrinsic apoptotic pathway [46]. Bone marrow stromal cells (BMSCs) in the control group exposed to H_2_O_2_ showed a decrease in mitochondrial membrane potential (MMP), with JC-1 aggregates dispersing from the mitochondria and turning into JC-1 monomers, emitting strong green fluorescence (Fig. 4I). This indicated the presence of JC-1 monomers and mitochondrial depolarization. However, cells treated with the TF@GL + NIR group clearly maintained the integrity of MMP, as evidenced by significant red fluorescence and weak green fluorescence, along with further statistical analysis (Fig. S13B). The JC-1 flow cytometry results were consistent with the JC-1 staining. The cell population in the control group concentrated in Q3 (JC-1 FITC^+^PE-A^−^), indicating MMP depolarization. In the TF@GL + NIR group, the cell population shifted toward Q2 (JC-1 FITC^+^PE-A^+^), indicating an increase in membrane potential and recovery of polarization (Fig. 4H and J).

In order to further investigate the underlying mechanism, the levels of key molecules of apoptosis (Bax, Bcl-2, and Caspase-3) and pyroptosis (NLRP3, Caspase-1 and N-GSDMD) were assessed. After receiving endogenous lethal stimuli, the pro-apoptotic protein Bax dimerizes and accumulates on the mitochondrial outer membrane to affect mitochondrial membrane permeability, leading to the release of cytochrome *c* (Cyt-c) and mitochondrial reactive oxygen species (mtROS) [47]. Cyt-c leads to an increase in Caspase-3, mediating apoptosis [48]. WB and PCR results of the control group confirmed that the apoptosis pathway was activated (Fig. 4K and L, S14A). However, in the TF@GL + NIR group, apoptosis was alleviated by the formation of a heterodimer between the anti-apoptotic protein Bcl-2 and Bax [49]. On the other hand, mtROS leads to an elevation of NLRP3, which results in an elevation of Caspase-1 and subsequently an elevation of GSDMD-NT [50]. Pyroptosis is triggered by GSDMD-NT-mediated pore formation in the cell membrane, causing membrane rupture and fragmentation [51]. As shown in Fig. 4M and N and S14B, the pyroptosis pathway was reduced by the treatments with GL, TF@GL, and TF@GL + NIR. In summary, these results indicate that H_2_Se delivery system can lower ROS levels, preserve mitochondrial integrity, inhibit cell apoptosis and pyroptosis (Fig. 4O).

### 3.5 Anti-inflammatory effects of TF@GL hydrogel and transcriptomic analysis revealing macrophage polarization pathways

**Fig. 5:**
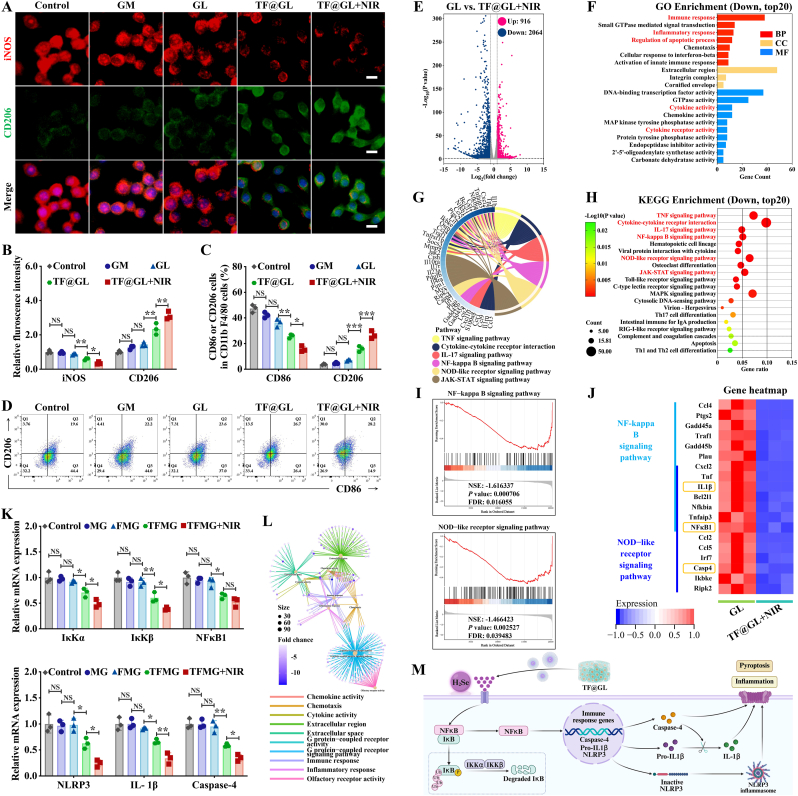
TF@GL hydrogel regulated macrophage inflammation and polarization. (A, B) IF staining of iNOS and CD206 of RAW264.7 cells (after LPS stimulation) cultured with different hydrogels on day 2, with quantitative fluorescence intensity (n = 3). Scale bars: 10 μm. (C, D) After gating on CD11b^+^F4/80^+^ cells, representative flow cytometry plots and quantitative data show the proportions of M1 cells (CD86^+^CD206^−^) and M2 cells (CD86^−^CD206^+^) polarized macrophages (n = 3). (E) Volcano plot of transcriptomic analysis of differentially expressed genes. (F) GO analysis of all genes in macrophages cultured on TF@GL + NIR versus GL. (G) Radial plot of gene enrichment in the immune-related KEGG pathways. (H) KEGG enrichment pathways of TF@GL + NIR versus GL. (I) GSEA of KEGG pathways. (J) Heatmap of differentially expressed genes of NF-κB and NLR signaling pathways. (K) Relative mRNA expression of NF-κB- and NLR-related genes (n = 3). (L) Gene-concept network plot of GO terms. (M) Schematic diagram of TF@GL hydrogel inhibiting NF-κB and NLR signaling pathway. Data are presented as mean ± SD; **∗***P* < 0.05, **∗∗***P* < 0.01, **∗∗∗***P* < 0.001, **∗∗∗∗***P* < 0.0001.

Live bacterial infection induces the polarization of macrophages toward the M1 phenotype, and ROS generated by diabetic pathological state also contributes to exacerbating local inflammation [52]. To address this concern, we utilize‌d immunofluorescence (IF) staining, flow cytometry, and transcriptomic analysis to investigate the anti-inflammatory effects of TF@GL hydrogel. The M1 phenotype polarization model was established by exposing RAW264.7 macrophages to LPS (100 ng mL^−1^) for 24 h. These cells were co-cultured with hydrogels and then analyzed by IF staining of iNOS (M1 marker), and CD206 (M2 marker). As illustrated in Fig. 5A, the control group exhibited round macrophages with prominent iNOS fluorescence, characteristic of typical M1 macrophages. In contrast, the TF@GL + NIR group displayed macrophages with long spindle-shaped morphologies, akin to typical M2 macrophages. Additionally, the statistical analysis of fluorescence intensity indicated that the trends in the groups without H_2_Se donor (control, GM, GL) were similar. Compared to the other groups, the groups with H_2_Se donors (TF@GL and TF@GL + NIR) had significantly decreased iNOS and increased CD206 fluorescence intensity, indicating that macrophages polarized from the M1 phenotype to the M2 phenotype (Fig. 5C). This indicated that the delivery of H_2_Se played a regulatory role in the anti-inflammatory polarization of macrophages, and the enhanced release of H_2_Se through MPTT further deepened this effect. Subsequently, CD86/CD206 flow cytometry accurately represented the proportions of M1 and M2 macrophages. Specifically, further statistical analysis revealed that RAW264.7 cells in the control group showed M1 (CD86^+^CD206^−^) and M2 (CD86^−^CD206^+^) cell percentages of 47.33 ± 1.76% and 3.82 ± 0.27% (Fig. 5B and D). However, treatment with TF@GL (M1 25.8 ± 0.92%, M2 15.83 ± 1.30%) and TF@GL + NIR (M1 15.71 ± 1.29%, M2 26.67 ± 1.76%) significantly reduced the proportion of M1 macrophages while increasing the number of M2 macrophages. M2 macrophages secreted anti-inflammatory cytokines and pro-repair mediators, effectively suppressing excessive inflammation and promoting angiogenesis, matrix remodeling, and tissue regeneration, thereby creating a microenvironment conducive to repair of the damaged tissue [53]. It demonstrated that the delivery of H_2_Se influenced the morphology and polarization of macrophages to potentially modulate immune function. As previously reported, MPTT can partially modulate immune responses in LPS-activated macrophages, as evidenced by moderate downregulation of TNF-α and IL-1β, along with a concomitant increase in IL-4 and IL-10 (Fig. S15). However, when combined with H_2_Se release from TF@GL + NIR, the anti-inflammatory response was significantly enhanced, indicating a synergistic interaction between MPTT and gasotransmitter delivery.

To explore the mechanism of H_2_Se gas in macrophage polarization, we performed RNA-seq transcriptomic analysis on LPS-stimulated macrophages with the TF@GL + NIR and GL groups. The pearson correlation and principal component analysis confirmed that the samples met the required criteria, thereby validating the reliability of the RNA-seq findings (Fig. S16A). In addition, the volcano plot illustrated 916 up-regulated and 2064 down-regulated genes in the TF@GL + NIR versus GL group, indicating significant differences in gene expression (Fig. 5E). As depicted in Fig. 5F and S17A, the Gene Ontology (GO) enrichment analysis showed that the top 20 enriched down-regulated terms among the differentially expressed genes. The genes were divided into three typical classes: biological process (BP), molecular function (MF), and cellular component (CC). Among them, inflammation- and pyroptosis-related pathways have received attention. The GSEA (gene set enrichment analysis) method was used to analyze the gene expression data for GO enrichment, revealing that the TF@GL + NIR group reduced the inflammatory response and immune response (Fig. S18A). Interestingly, in the presence of H_2_Se, the GSEA graphs for the microtubule and positive regulation of cell population proliferation signaling pathways displayed upward curves. Microtubules are essential tools for mitosis and are key determinants in regulating cell proliferation [54]. In the bone healing microenvironment, the increase in microtubules facilitates cell proliferation, which greatly aids subsequent bone integration.

Fig. 5H and S17B showed the top 20 down-regulated enriched pathways based on KEGG (Kyoto Encyclopedia of Genes and Genomes) analysis, presented in both Bubble chart and Clustering chart. These results indicated that inflammatory and pyroptotic pathways, particularly the TNF, cytokine-cytokine receptor interaction, IL-17, NF-κB (NF-kappa B), NLR (NOD-like receptor), and JAK-STAT signaling pathways, played a crucial role in the gene expression changes. The radial plot highlighted the interactions and correlations of these pathways (Fig. 5G). Predictably, inhibition of these pathways may help to reduce osteogenic damage, thereby establishing an immune microenvironment conducive to bone regeneration. The GSEA graphs showed that these pathways displayed downregulation curves, which increased the credibility of the anti-inflammatory effects of TF@GL + NIR (Fig. 5I–S18B). NF-κB and NLR signaling pathways played significant roles in inflammation and pyroptosis. Then, a normalized heatmap analysis was performed on the highest-ranked genes involved in the NF-κB and NLR signaling pathways. Significantly, three genes (IL-1β, NFκB1, and Caspase-4) in the heatmap garnered attention and helped clarify the underlying inflammatory mechanisms (Fig. 5J).

Thus, qRT-PCR assays were conducted to assess the expression of genes associated with NF-κB and NLR signaling pathway. It was found that the H_2_Se derived from the TF@GL hydrogel exhibited prominent anti-inflammatory and anti-apoptotic effects (Fig. 5K). Specifically, it led to the downregulation of critical genes, including IκKα, IκKβ, NF-κB1, IL-1β, NLRP3, and Caspase-4. NF-κB is a key regulator of inflammatory mediators and promotes the transcription of pro-inflammatory cytokines. By inhibiting NF-κB activation through the action of H_2_Se, the TF@GL hydrogel helped diminish the expression of these inflammatory markers, thereby restoring balance within the immune response. IκKα and IκKβ are actually kinases of NF-κB in the cytoplasm, promoting their translocation to the nucleus and thus activate target genes. IL-1β is a potent pro-inflammatory cytokine. Caspase-4 and NLPR3 is associated with programming cell death. Caspase-4 also leads to the IL-1β mediated maturation and secretion. Objectively, oxidative stress, infection, and the implantation of fillers exacerbate the inflammatory response in diabetic osteomyelitis. The gene-concept network plot visually highlighted the prominent roles of the immune response and inflammatory response pathways within this context (Fig. 5L). In this situation, the machine diagram underscored the therapeutic potential of the TF@GL hydrogel in managing inflammation and promoting cellular health by influencing important signaling pathways (Fig. 5M).

### 3.6 In vitro osteogenesis and angiogenesis of TF@GL hydrogel

**Fig. 6:**
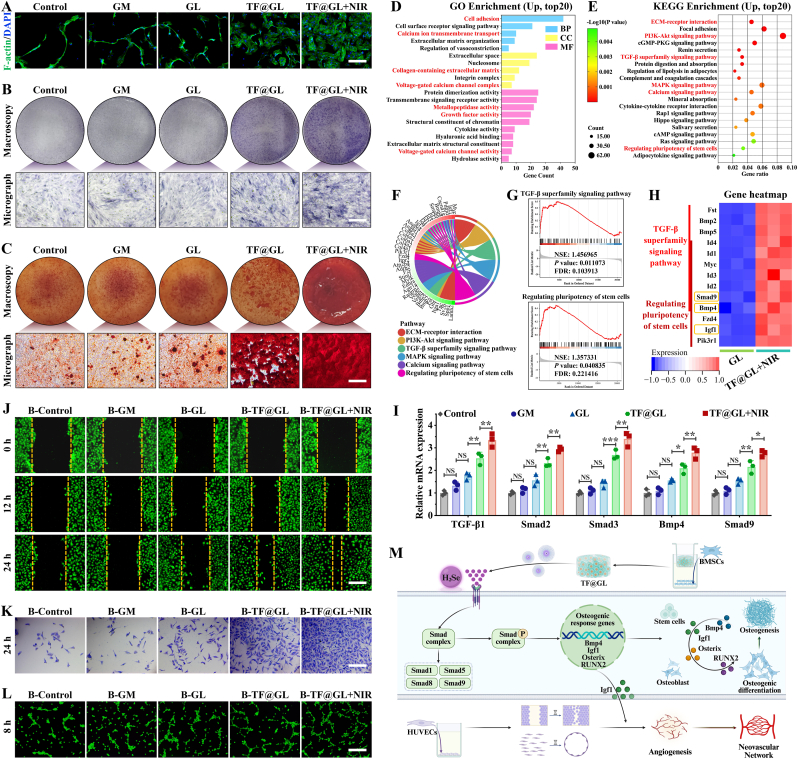
Bioinformatic analysis of BMSCs, and osteogenesis-angiogenesis on TF@GL hydrogel. (A) Nucleus/cytoskeleton staining of BMSCs adhered to the surface of hydrogels initially at 20% confluency for 2 days. Scale bars: 50 μm. (B) ALP staining of BMSCs on day 7. Scale bars: 200 μm. (C) Alizarin Red S staining of BMSCs on day 21. Scale bars: 200 μm. (D) GO analysis of all genes in BMSCs cultured on TF@GL + NIR versus GL. (E) KEGG enrichment pathways. (F) Radial plot of gene enrichment in the osteogenic-related KEGG pathways. (G) GSEA of KEGG pathways. (H) Heatmap of differentially expressed genes of TGF-β and Regulating pluripotency of stem cells (RPSC) signaling pathways. (I) Relative mRNA expression of TGF-β/BMP and RPSC-related genes (n = 3). (J) Scratch wound healing tests on the migration of HUVECs cultured with hydrogels from 0 to 24 h. Dotted lines indicate scratch edges. Scale bars: 200 μm. (K) 24-h Transwell migration of HUVECs. Scale bars: 200 μm. (L) 8-h Matrigel tube formation of HUVECs. Scale bars: 200 μm. (M) Schematic diagram of TF@GL hydrogel promoting TGF-β/BMP and RPSC signaling pathways. Data are presented as mean ± SD; **∗***P* < 0.05, **∗∗***P* < 0.01, **∗∗∗***P* < 0.001.

Osteogenic differentiation plays a crucial role in the repair of bone defects, especially in diabetic and infected bone defects, where impaired healing and tissue regeneration are common [55]. Therefore, the differentiation of BMSCs into osteoblasts is essential for successful bone regeneration. We first performed FITC staining of the BMSCs cytoskeleton (initially at 20% confluency for 2 days). The BMSCs in the TF@GL and TF@GL + NIR groups exhibited a linearly extended polygonal shape, while those in the other three groups displayed a spindle shape (Fig. 6A). Interestingly, the TF@GL and TF@GL + NIR groups had more cells. Next, we adjusted the cell concentration to test cytocompatibility (initially at 80% confluency for 3 days), and the TF@GL + NIR group showed the highest cell count, which was 1.53 times that of the control group (Fig. S19). Live/dead staining indicated that there were no significant red dead cells after the TF@GL hydrogel and MPTT (Fig. S20). The increased presence and morphological changes of BMSCs suggested that H_2_Se could promote in vitro cell proliferation, which is a prerequisite for osteogenesis. To evaluate whether TF@GL + NIR enhances early osteogenic differentiation, we performed alkaline phosphatase (ALP) staining on BMSCs of osteogenic induction for 7 days. Blue ALP staining indicated better differentiation in the TF@GL and TF@GL + NIR groups (Fig. 6B). Considering that calcium nodule deposition in the extracellular matrix is closely related to osteogenic differentiation, Alizarin red S (ARS) staining was conducted. Fig. 6C showed that the TF@GL + NIR group induced the highest degree of matrix mineralization after 21 days of osteogenic induction. The quantitative analysis of ALP and ARS staining accurately reflected the effective osteogenic activity of TF@GL hydrogel, indicating its potential for subsequent in vivo tissue repair (Fig. S21). Considering that MPTT inherently possesses a certain osteoinductive potential. To further dissect whether the enhancement in osteogenesis is attributable solely to H_2_Se release or influenced by the direct MPTT on BMSCs, we assessed the secretion of key ECM proteins, including Col-1, OPN, and OCN (Fig. S22). NIR irradiation alone moderately upregulated Col-1 and OPN compared to the control group, while OCN (late mineralization marker) showed no significant change, confirming that MPTT exerted the modest osteogenic effect. However, the TF@GL + NIR group exhibited significantly elevated levels of all three markers, indicating that H_2_Se synergized with MPTT to enhance osteogenic induction capacity. This result revealed that H_2_Se not only accelerated early matrix deposition but also drove the terminal mineralization process, consistent with the chronological sequence of bone matrix maturation.

Using transcriptomic sequencing, we identified the underlying mechanisms through which H_2_Se gas influence the differentiation of BMSCs. The principal component analysis, FPKM box plot, pearson correlation and volcano plot confirmed that the samples met the required criteria, thereby validating the reliability of the RNA-seq results (Fig. S23). As shown in Bubble and Clustering charts, the GO enrichment analysis indicated that the top 20 enriched upregulated terms were particularly related to cell adhesion, cell proliferation, and calcium ion pathways associated with osteogenesis (Fig. 6D–S24A). GSEA of GO pathways revealed upregulation of growth factor activity and collagen-containing extracellular matrix (Fig. S24B). The former drove cell proliferation, differentiation, and matrix mineralization, while the latter provided the structural scaffold for bone tissue and mediated cell-matrix signaling. Furthermore, the KEGG pathway enrichment analysis identified key signaling pathways that were impacted by TF@GL treatment, including ECM-receptor interaction, PI3K-Akt, TGF-β superfamily, MAPK, calcium, and Regulation of pluripotency in stem cells (RPSC) (Fig. 6E–S24C). The radial plot of the top 10 enriched genes for these pathways showed the overlap among the pathways, highlighting their close correlation (Fig. 6F). After performing GSEA on these six pathways, the activation of signaling pathways such as TGF-β superfamily and RPSC was further confirmed (Fig. 6G–S24D).

Subsequently, a heatmap analysis was conducted focusing on the top 10 differentially expressed genes within these two pathways, visually demonstrating how TF@GL hydrogel supports bone formation by upregulating the expression of important genes. Three key genes (Smad9, Bmp4, Igf1) were found to act as connectors between these two pathways, suggesting a cross-talk mechanism that enhances osteogenesis (Fig. 6H). Notably, Smad9 belongs to the BMP pathway-specific R-Smad family and primarily responds to BMP ligands (e.g, BMP4), rather than acting as a direct effector in the canonical TGF-β/Smad2/3 pathway. It was reasonably hypothesized that the TF@GL hydrogel initially activated the TGF-β signaling pathway, evidenced by the upregulation of TGF-β1 and Smad2/3, along with an increase in p-Smad2/3. This activation subsequently induced the expression of Bmp4 through the Smad pathway. The upregulated Bmp4 then activated BMP receptors, leading to the phosphorylation of Smad9 (p-Smad9). The phosphorylated Smad9 translocated to the nucleus, where it cooperatively interacted with TGF-β signaling to drive the transcription of key osteogenic master genes including Osterix and RUNX2. To validate this cascading model, the expression of osteogenesis-related genes, including TGF-β1, Smad2, Smad3, Bmp4, Smad9, Igf1, Osterix, and RUNX2, was measured using qRT-PCR (Fig. 6I–S25). All these genes were significantly upregulated in the TF@GL + NIR group, followed by the TF@GL group. Western blot analysis further confirmed that both TF@GL and TF@GL + NIR treatments markedly increased the protein levels of p-Smad2/3 (markers of TGF-β pathway activation) and p-Smad9 (a marker of BMP pathway activation) (Fig. S26), indicating effective activation of both the TGF-β and BMP signaling pathways. Although TGF-β could exert pro-inflammatory effects in chronic inflammation, its might impair bone remodeling. Therefore, H_2_Se did not simultaneously activate conflicting the conflicting actions of TGF-β. Instead, it coordinated the regenerative process through a sequential manner that first resolved inflammation and then promoted osteogenic differentiation through TGF-β. Additionally, the upregulation of Igf1 likely promoted the growth of stem cells, providing an ample cellular reservoir for bone regeneration. Meanwhile, the pronounced elevation of Osterix and RUNX2, which played a role in regulating the formation and mineralization of bone tissue during the process of osteogenic differentiation. These findings demonstrated that the TF@GL hydrogel coordinated microenvironmental remodeling and initiation of the osteogenic program through the TGF-β/BMP cascade pathway.

Integrating osteogenesis-angiogenesis can enhance the efficiency of bone regeneration. Additionally, Igf1 is a positive factor for cell migration and angiogenesis. Encouraged by the promotion of H_2_Se gas on BMSCs to secrete Igf1, we investigated the effects of conditioned media collected from the BMSCs culture system (B-Control, B-GM, B-GL, B-TF@GL, B-TF@GL + NIR) on HUVECs. As shown in Fig. 6J, compared to the B-Control, the B-TF@GL and B-TF@GL + NIR media accelerated the wound closure of HUVECs. After 24 h, the wound area in the B-TF@GL + NIR group was largely covered by migrating HUVECs, with a wound closure rate of 81.95 ± 2.55%, which is 4.22 times that of the B-Control (Fig. S27A). The wound closure rates for B-GM and B-GL were 22.07 ± 1.72% and 30.46 ± 0.84%, respectively. Similarly, crystal violet staining images revealed that the greatest number of HUVECs migrated through the Transwell membrane in the B-TF@GL + NIR group (Fig. 6K). Additionally, tube formation assays revealed the establishment of interconnected tubular networks in the B-TF@GL and B-TF@GL + NIR groups (Fig. 6L). Notably, the B-TF@GL + NIR group exhibited the highest density and branching in the tubular network, followed by the B-TF@GL group. In contrast, the B-Control, B-GM, and B-GL groups displayed dendritic protrusions. The nodes and segments in the B-TF@GL + NIR group were 3.76 times and 3.29 times that of the B-Control, respectively (Fig. S27B). Overall, the mechanistic diagram indicated that H_2_Se promoted osteogenic differentiation by binding to receptors on the cell membrane and activating key signaling pathways (TGF-β/BMP and RPSC) (Fig. 6M). Endothelial cells in this microenvironment received a positive influence from Igf1, leading to angiogenesis. These results supported the potential of TF@GL hydrogel to enhance bone healing and vascular regeneration, which was crucial for effective tissue repair.

### 3.7 Photothermal TF@GL hydrogel promoted bone repair in vivo

**Fig. 7:**
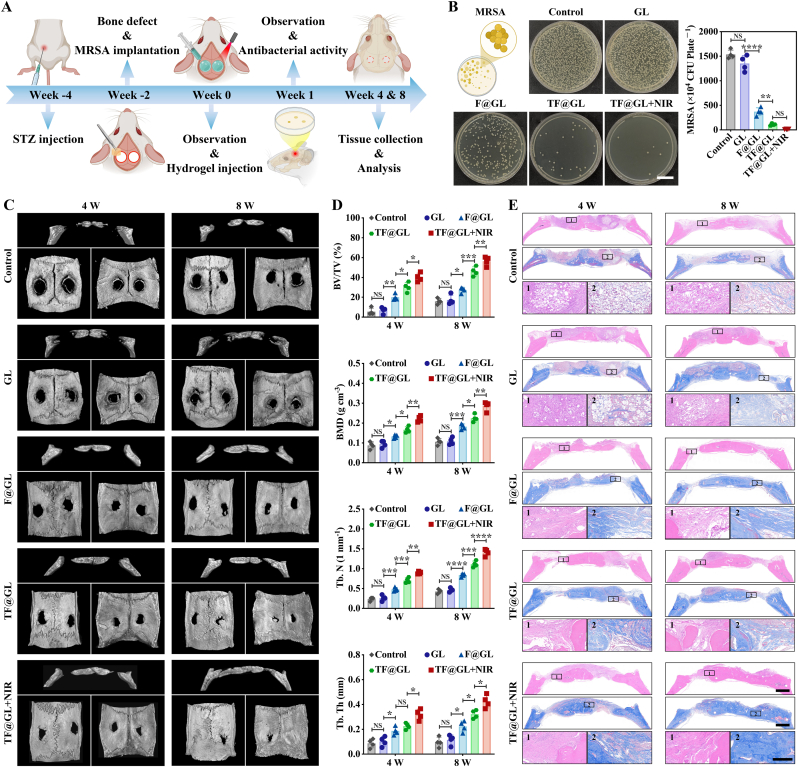
TF@GL hydrogel promoted treatment of osteomyelitis in vivo. (A) Scheme of osteomyelitis model and treatment process. (B) MRSA colonies of infected cranium region at week 1 post-treatment and bacterial quantitation (n = 4). Scale bars: 2 cm. (C) Micro-CT images of crania at week 4 and 8 post-treatment. (D) Quantitative results of BV/TV, BMD, Tb.N, Tb. Th (n = 4). (E) H&E staining and Masson staining of cranial defect at week 4 and 8 post-treatment. Scale bars: 2 mm in low-magnification and 200 μm in high-magnification. Data are presented as mean ± SD; **∗***P* < 0.05, **∗∗***P* < 0.01, **∗∗∗***P* < 0.001, **∗∗∗∗***P* < 0.0001.

Encouraged by the remarkable antibacterial and osteogenic properties of TF@GL hydrogel in vitro, the antibacterial and osteogenicity functions were further evaluated in infected diabetic bone defects (Fig. 7A). The successful modelling of type 2 diabetic rats was confirmed by continuous monitoring of blood glucose concentration (Fig. S28A). The blood glucose levels of rats consistently remained within the diabetic range throughout the experimental period, including at week 8 post-treatment (Fig. S28B). The successful modeling of osteomyelitis was confirmed by Micro-CT imaging and the bacterial plating (Fig. S29). The TF@GL hydrogel was implanted into the defect sites, followed by NIR irradiation to elevate the local temperature to 42.58 ± 1.16 °C, a range that avoids additional photothermal damage (Fig. S30). After hydrogel injection therapy, antibacterial tests were first conducted, where bacteria were collected from the defect site and surrounding tissues. The detection rate of MRSA was consistent with in vitro results, showing that the TF@GL + NIR group had significantly lower levels of MRSA compared to the other groups (Fig. 7B). Giemsa staining also revealed very few free bacteria within the tissues in the TF@GL and TF@GL + NIR groups (Fig. S31). This superior antibacterial effect was sustained over time. At both 4 and 8 weeks post-treatment, MRSA burdens in the TF@GL + NIR group remained nearly undetectable, with no evidence of infection recurrence (Fig. S32). These findings clearly indicated that TF@GL hydrogel, serving as a delivery platform for H_2_Se gas, acted as a reliable weapon against bacterial invasion due to its ability to combine MPTT with NIR irradiation to accelerate the release of H_2_Se gas.

Micro-CT (macro aspects) and histological analyses (micro aspects) were conducted to characterize the bone formation process at week 4 and 8 post-treatment. At week 4, irregular bone destruction was observed at the defect sites in the control and GL groups, characterized by lytic areas and patchy radiolucent zones with unclear boundaries (Fig. 7C). In the F@GL group, there was only minimal new bone formation. In contrast, significant new bone was observed in the TF@GL and TF@GL + NIR groups, with well-defined margins, indicating that the infection had been controlled. At week 8, the control and GL groups continued to exhibit bone destruction and necrosis consistent with osteomyelitis. More concerning, small, round areas of bone destruction appeared adjacent to the defects, suggesting persistent infection. The non-healing of cranial defects corresponded to the chronic phase of clinical osteomyelitis. In contrast, the TF@GL + NIR group showed ongoing new bone growth, nearing closure. Furthermore, quantitative analysis revealed significant increases in bone volume/tissue volume (BV/TV) and bone mineral density (BMD), indicating substantial increases in both the volume and density of new bone (Fig. 7D). This suggested that the TF@GL + NIR group significantly induced new bone formation, followed by TF@GL and F@GL. Additionally, increases in trabecular number (Tb.N) and trabecular thickness (Tb.Th) indicated a notable improvement in the trabecular structural geometry, providing a scaffold for the growth of new bone tissue, guiding orderly cell arrangement, and facilitating mineralization, thus accelerating the repair of bone defects. This may relate to the three-dimensional structure mimicked by the microgel-based hydrogel. The hydrogel formed from microgels provided a spatial environment for cell growth and facilitated the interaction of H_2_Se gas with the cells.

To strengthen validation, we included additional groups: vancomycin (clinical antibiotic), TDN + NIR (free H_2_Se donor), and tf@gl + NIR (no microgel-based TF@GL). At week 1, vancomycin showed strong antibacterial activity, but bacterial load rebounded by week 4 (Fig. S33A). TDN + NIR also reduced bacteria initially but failed to prevent recurrence, underscoring the need for a delivery system. In contrast, both tf@gl + NIR and TF@GL + NIR achieved sustained bacterial clearance, with the lowest MRSA burden. Micro-CT at week 4 showed limited repair in vancomycin group, while TDN + NIR revealed bone destruction due to infection relapse (Fig. S33B). The tf@gl + NIR group exhibited moderate bone formation, but only TF@GL + NIR displayed robust regeneration with well-defined margins. Quantitative analysis confirmed that TF@GL + NIR significantly outperformed all groups in BV/TV, BMD, Tb.N, and Tb.Th, demonstrating that the microgel structure is essential for effective bone repair.

H&E and Masson staining were performed on the cranial defect. As shown in Fig. 7E, the HE staining of the control group at week 4 revealed disorganized collagen fibers and extensive foam-like necrotic cells, indicating a poor state of the diabetic infection microenvironment. The Masson staining results for the F@GL and TF@GL groups showed primarily fibrous soft tissue filled with blue-stained new collagen, suggesting delayed collagen maturation. In contrast, the TF@GL + NIR group exhibited well-aligned collagen fibers at the edges of the defect, along with bone-like matrix formation, and a significant amount of red-stained mature collagen at the center of the defect. At week 8, the control group still showed disorganized fibrous tissue and a large number of inflammatory cells. However, most areas of the defect in the TF@GL + NIR group were filled with mature bone-like structures, validating that this antibacterial and osteogenic hydrogel enhanced the healing effects in the infected defect areas.

The timely degradation of the hydrogel is critical for in vivo tissue regeneration. The TF@GL and TF@GL + NIR groups exhibited no significant differences, and both groups completely degraded within 4 weeks, indicating that the degradation of the hydrogel does not interfere with the bone regeneration process (Fig. S34). Moreover, the locally released H_2_Se attained a concentration of 1.94 ± 0.25 μg mL^−1^ in the bone defect, within the therapeutic window and below cytotoxic threshold, ensuring effective delivery (Table S5). The biosafety of TF@GL hydrogel was further evaluated at week 8 post-treatment. H&E staining demonstrated no significant pathological changes in major organs (Fig. S35). Except for white blood cell (WBC) count, serum biochemical and hematological analyses revealed no significant differences between the TF@GL group and the control group, with key parameters falling within normal ranges (Fig. S36). Notably, WBC count was elevated in the control group, consistent with chronic osteomyelitis. In contrast, WBC levels remained within the normal range in the TF@GL group, indicating effective clearance of infection. These findings further confirm the biosafety of the TF@GL hydrogel in vivo. To evaluate the in vivo fate of trace elements, content of Fe and Se in major organs were measured (Fig. S37). The groups containing nanoparticles (F@GL, TF@GL, and TF@GL + NIR) showed increased Fe content in the liver, spleen, and kidneys, suggesting Fe was metabolized via those organs. The groups containing the H_2_Se donor (TF@GL and TF@GL + NIR) showed slight increases in Se content in the liver and kidneys, indicating in vivo clearance pathways for Se.

### 3.8 In vivo anti-inflammatory and osteogenesis of TF@GL hydrogel

**Fig. 8:**
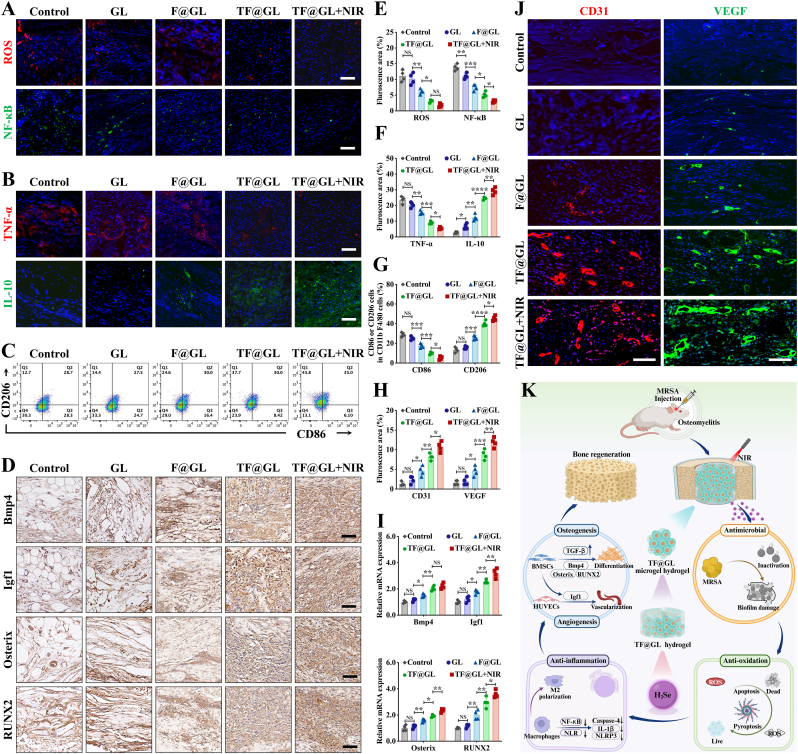
TF@GL hydrogel enhanced anti-inflammation and osteogenesis in the cranial defect of diabetic osteomyelitis rats. (A) IF staining of nucleus, ROS (red) and NF-κB (green) in cranial defect at week 4 post-treatment. Scale bars: 50 μm. (B) IF staining of nucleus, TNF-α (red) and IL-10 (green) in cranial defect at week 4 post-treatment. Scale bars: 50 μm. (C) Representative flow cytometry plots showing macrophage polarization around cranial defect at week 4 post-treatment. (D) IHC staining of Bmp4, Igf1, Osterix and RUNX2 in cranial defect at week 4 post-treatment. Scale bars: 50 μm. (E) Quantitative fluorescence area of MMP9 and ROS based on IF images (n = 4). (F) Quantitative fluorescence area of TNF-α and IL-10 based on IF images (n = 4). (G) Quantitative results of M1 (CD86^+^CD206^−^) and M2 (CD86^−^CD206^+^) cells after gating on CD11b^+^F4/80^+^ cells (n = 4). (H) Quantitative fluorescence area of CD31 and Ang-1 based on IF images (n = 4). (I) Relative mRNA expression of Bmp4, Igf1, Osterix, RUNX2 genes (n = 4). (J) IF staining of nucleus, CD31 (red) and VEGF (green) in cranial defect at week 4 post-treatment. Scale bars: 100 μm. (K) Schematic diagram of in vivo osseointegration mechanism. Data are presented as mean ± SD; **∗***P* < 0.05, **∗∗***P* < 0.01, **∗∗∗***P* < 0.001, **∗∗∗∗***P* < 0.0001.

It was established that the first week following implantation represented a crucial window for inflammatory cell infiltration and macrophage polarization at the defect site. Timely resolution of excessive pro-inflammatory responses during this period is essential for facilitating subsequent tissue repair. ELISA analysis of rat serum revealed dynamic changes in key cytokines (Fig. S38). At day 3 post-treatment, the control group exhibited significantly elevated levels of pro-inflammatory cytokines IL-6 and TNF-α, which are classical markers associated with M1 macrophage polarization and indicate a robust acute inflammatory response. In contrast, the TF@GL + NIR group markedly suppressed the release of IL-6 and TNF-α. Notably, this group also showed significantly higher levels of anti-inflammatory cytokines IL-4 and IL-10, which are factors closely linked to M2 macrophage polarization, as early as day 3, with these elevated levels sustained through day 7. IF and immunohistochemical (IHC) analysis was conducted to further investigate the inflammation response and osteogenesis of the hydrogels in infected diabetic bone defects. It is well-known that excessive oxidation associated with diabetes can lead to damage of bone tissue due to persistent inflammation. Thus, reducing oxidative stress and controlling inflammation are essential for effective bone repair. ROS IF staining showed that the TF@GL and TF@GL + NIR groups were effective in clearing peroxides, while NF-κB IF staining demonstrated that these two groups reduced NF-κB expression, confirming the cellular results (Fig. 8A and E). Besides, to further assess the role of H_2_Se gas in regulating macrophage M2 polarization in the diabetic and infected microenvironment, IF staining was performed for the inflammatory factor TNF-α and the anti-inflammatory factor IL-10. At week 4, red TNF-α expression in the control group was higher than that in the other groups, while green IL-10 expression was the lowest, which suggested that M1 macrophages were dominant in the progression of chronic osteomyelitis. (Fig. 8B and F). Quantification of fluorescence intensity revealed that the density of TNF-α positive cells in the TF@GL + NIR (5.48 ± 0.33%) and TF@GL (9.00 ± 0.45%) groups was lower than that in the F@GL (15.50 ± 0.60%), GL (20.39 ± 0.84%), and control (23.55 ± 1.12%) groups. This might be due to the released H_2_Se promoting the polarization of macrophages toward the M2 phenotype, thereby alleviating the inflammatory response in the defected bone tissue. The fluorescence intensity of IL-10 positive cells in the TF@GL + NIR group (29.11 ± 1.09%) was higher than in the TF@GL group (24.48 ± 0.40%), which may be attributed to the MPTT triggering the release of more H_2_Se, enhancing the local anti-inflammatory response in the bonetissue. To accurately quantify the proportion of M2 macrophages, we collected tissue from the cranial defect and surrounding regions for further flow cytometry analysis. The proportion of M2 macrophages (CD86^−^CD206^+^) in the TF@GL + NIR and TF@GL groups was significantly higher than in the other three groups, while the proportion of M1 macrophages (CD86^+^CD206^−^) in the TF@GL + NIR and TF@GL groups was significantly lower than in the other three groups (Fig. 8C and G), which was consistent with the IF staining results. It indicated that H_2_Se delivery had a significant anti-inflammatory effect when facing the dual challenges of diabetes and infection, thereby creating a better environment for bone cells to regenerate, accelerating healing [56].

In vitro results indicated that TF@GL hydrogel promoted osteogenic differentiation through the TGF-β/BMP and RPSC pathways, leading us to investigate whether these molecules were expressed in vivo. As expected, IHC images revealed a higher number of positive cells for Bmp4, Igf1, Osterix, and RUNX2 in the TF@GL + NIR group, along with denser new bone tissue (Fig. 8D). This was attributed to the sustained release of H_2_Se, which underpinned the hydrogel to promote osteogenic mineralization. Unlike typical in vitro monoculture conditions, bacterial toxins in a hyperglycemic environment could induce cellular senescence or even death. However, H_2_Se exhibited an anti-apoptosis effect on surrounding cells, thus maintaining tissue homeostasis in the bone regeneration area. Corresponding trends in gene expression for Bmp4, Igf1, Osterix, and RUNX2 further corroborated that the TF@GL + NIR group promoted bone formation (Fig. 8I). Bone growth was known to be accompanied by angiogenesis, which supplied essential materials like oxygen and nutrients for cell growth and bone matrix formation. With vessels marked by CD31 and VEGF, the TF@GL + NIR group exhibited more pronounced vascular cross-sections and extensive angiogenesis, suggesting an enhancement in vascular formation to accelerate bone regeneration (Fig. 8H and J). It validated the close relationship between bone growth and angiogenesis. Additionally, qRT-PCR analysis confirmed the highest expression of CD31 and VEGF, supporting angiogenic capability of H_2_Se gas at the gene level (Fig. S39). These findings underscored the positive role of the TF@GL hydrogel combined with MPTT in alleviating oxidative stress and inflammation while stimulating angiogenesis and osteogenesis, thereby improving the local microenvironment for bone regeneration in the diabetic infection.

## 4 Conclusion

In summary, we have developed a TF@GL microgel-based hydrogel system designed to address the critical challenges associated with diabetic osteomyelitis, leveraging the synergistic effects of H_2_Se gas delivery and NIR-triggered MPTT. It provides a multifunctional approach that combines antibacterial, antioxidative, anti-inflammatory, and osteogenic properties. The TF@GL hydrogel overcomes the limitations of traditional antibiotic therapies by not only exhibiting strong antibacterial property against resistant strains but also effectively disrupting biofilms, offering a comprehensive strategy for infection control. Additionally, the efficient delivery of H_2_Se scavenges reactive oxygen species (ROS) to reduce cell death, while inhibits inflammatory pathways (NF-κB and NLR), thereby modulating a regenerative microenvironment. Moreover, H_2_Se shows the potential for osteogenic differentiation and angiogenesis through the activation of key signaling pathway (TGF-β/BMP). In an infection model of diabetic rats, the injectable TF@GL hydrogel showed excellent performance in promoting bone repair. This hydrogel effectively combats MRSA, promotes new bone formation, and enhances angiogenesis while alleviating the detrimental impacts of inflammation and oxidative stress in vivo, offering new insights into the use of gaseotransmitters. This comprehensive strategy paves the way for effective management of complicated infections and improved treatment outcomes for bone defects in diabetes.

## CRediT authorship contribution statement

**Junwei Su:** Writing – original draft, Methodology, Formal analysis, Data curation, Conceptualization. **Xinyue Liang:** Writing – original draft, Methodology, Formal analysis, Data curation, Conceptualization. **Junwei Yang:** Writing – original draft, Methodology, Formal analysis, Data curation, Conceptualization. **Xianzhen Dong:** Investigation, Formal analysis. **Yu Chen:** Investigation, Formal analysis. **Xinyi Tan:** Investigation. **Zhiqiang Li:** Investigation. **Yuchen Song:** Investigation. **Hao Zhang:** Writing – review & editing, Supervision, Resources, Funding acquisition. **Honglian Dai:** Writing – review & editing, Supervision, Resources, Funding acquisition. **Aixi Yu:** Writing – review & editing, Supervision, Resources, Project administration, Funding acquisition.

## Ethics approval and consent to participate

All the animal experiments were implemented according to the guidelines for laboratory animals established by the Wuhan University Center for Animal Center Experiment/A3-Lab. All rat experiments were approved by the Institutional Animal Care and Utilization Committee (IACUC) of the Animal Experiment Center of Wuhan University (Approval number: WP20230642).

## Declaration of competing interests

The authors declare that they have no known competing financial interests or personal relationships that could have appeared to influence the work reported in this paper.
