# Supplementary material for: Mild photothermal-responsive hydrogel with H2Se delivery for anti-infection and microenvironment remodeling to bone regeneration in diabetic osteomyelitis
Source: Bioact Mater. 2026 May 2;64:159–79. doi: 10.1016/j.bioactmat.2026.04.041 (PMC13147789; doi:10.1016/j.bioactmat.2026.04.041)
Supplement: Multimedia component 1 [file mmc1.docx]

**Supporting Information**

Mild Photothermal-Responsive Hydrogel with H_2_Se Delivery for Anti-infection and Microenvironment Remodeling to Bone Regeneration in Diabetic Osteomyelitis

**Table S1.** Elemental analysis of of Gel and Gel-LA.

| **Materials** | **N (%)** | **H (%)** | **C (%)** |
| --- | --- | --- | --- |
| **Gel** | 16.51 | 7.42 | 44.235 |
| **Gel-LA** | 15.205 | 7.785 | 42.73 |

Based on the nitrogen and hydrogen content in the repeating units of gelatin (Gel), lipoic acid (LA), and the grafting rates of LA onto Gel were calculated. The chemical formula of Gel is C_102_H_151_O_39_N_31_, with a repeating unit molecular weight of 2,433 g mol^−1^, nitrogen content of 17.838%, hydrogen content of 6.206%, and carbon content of 50.308%. The chemical formula of LA is C_8_H_14_O_2_S_2_, with a molecular weight of 206.32 g mol^−1^; after grafting, its molecular weight becomes 189.32 g mol^−1^, nitrogen content is 0%, hydrogen content is 6.867%, and carbon content is 50.708%.

Based on the changes in nitrogen and hydrogen content in Gel-LA, it was calculated that approximately 1.1 molecules of LA were grafted onto each repeating unit of Gel.

**Table S2.** The rat-specific forward and reverse primer sequences for RT-PCR performance.

| **Gene** | **Forward primer (5' to 3')** | **Reverse primer (5' to 3')** |
| --- | --- | --- |
| **NLRP3** | AGTGGATAGGTTTGCTGGGATA | CTGGGTGTAGCGTCTGTTGAG |
| **Caspase-1** | TCATGGTCTCCAGGAGGGAAT | TTGGGCACTTCAATGTGTTCA |
| **GSDMD** | CTGGGAGATCATGCAACGTG | ACCTCCTCCTTTGTCTGCAG |
| **Bax** | GGATACAGACTCCCCCCGAG | AACATGTCAGCTGCCACACG |
| **Bcl-2** | CAGAGGGCTACGAGTGG | CAGAGCGATGTTGTCCAC |
| **Caspase-3** | GCAAAAGGAGCAGTTTTGTGTGTG | GGCAGGCCTGAATGATGAAGAG |
| **TGF-β1** | GCAACAACGCAATCTATGAC | CCTGTATTCCGTCTCCTT |
| **Smad2** | TGCCGCCTCTGGATGACTA | GAAATTTGTGTT |
| **Smad3** | GCCTGCTGTCCAATGTTAACC | CGCACACCCCTCCCAAT |
| **Bmp4** | AGAAATGGTGCCTGGACACCTCAT | TGGTCCCGGTTGTACAGTCCTAAT |
| **Smad9** | GAAAACACCAGGAGGCACATTGG | GCTCTGGACAAAGATGCTGCTG |
| **Igf1** | GCACTCTGCTTGCTCACCTT | AAAAAGCCCCTTGGTCCACAC |
| **Osterix** | CAGCCTGCAGCAAGTTTGG | TTTTCCCAGGGCTGTTGAGT |
| **RUNX2** | ACTTCCTGTGCTCGGTGCT | GACGGTTATGGTCAAGGTGA |
| **CD31** | CACCGTGATACTGAACAGCAA | GTCACAATCCCACCTTCTGTC |
| **VEGF** | TCGGGCCTCCGAAACCATGA | CCGCCTCGGCTTGTCACATCT |
| **GAPDH** | GGTGGACCTCATGGCCTACA | CTCTCTTGCTCTCAGTATCCTTGCT |

**Table S3.** The mouse-specific forward and reverse primer sequences for RT-PCR performance.

| **Gene** | **Forward primer (5' to 3')** | **Reverse primer (5' to 3')** |
| --- | --- | --- |
| **IκKα** | TGCACTTGGCAATCATCCAC | TTCCTCGAAAGTCTCGGAGCT |
| **IκKβ** | GGCACCTTGGATGACCTAGA | CCATATCCTGGCTGTCACCT |
| **NFκB1** | GGCGGCACGTTTTACTCTTT | CCGTCTCCAGGAGGTTAATGC |
| **NLRP3** | CGAGACCTCTGGGAAAAAGCT | GCATACCATAGAGGAATGTGATGTACA |
| **IL-1β** | TGGCAACTGTTCCTG | GGAAGCAGCCCTTCATCTTT |
| **Caspase-4** | CATCACTAGACTCATTTCCTGCTT | CTGGAATTTCAGGAATAGAATGTG |
| **GAPDH** | CCTCGTCCCGTAGACAAAATG | TGAGGTCAATGAAGGGGTCGT |

**Table S4.** The human-specific forward and reverse primer sequences for RT-PCR performance.

| **Gene** | **Forward primer (5' to 3')** | **Reverse primer (5' to 3')** |
| --- | --- | --- |
| **CD31** | GTGCTCTATGCAAGCCTCCA | TTCGAGGTGGTGCTGATGTC |
| **VEGF** | CTCCACCATGCCAAGTGGTC | GCAGTAGCTGCGCTGATAGA |
| **GAPDH** | GGAAGCTTGTCATCAATGGAAATC | TGATGACCCTTTTGGCTCCC |

**Table S5.** H_2_Se concentration in bone defect of the TF@GL+NIR group.

| **Sample** | **Total Se Mass (μg)** | **ΔSe (μg)** | **Equiv. H_2_Se concentration(μg mL^−1^)** |
| --- | --- | --- | --- |
| **Control 1** | 0.087906042 |  |  |
| **Control 2** | 0.082949056 |  |  |
| **Control 3** | 0.070935497 |  |  |
| **Control 4** | 0.073413635 |  |  |
| **TF@GL+NIR 1** | 0.130791516 | 0.051990459 | 2.263076349 |
| **TF@GL+NIR 2** | 0.124247746 | 0.045446689 | 1.97823466 |
| **TF@GL+NIR 3** | 0.12190744 | 0.043106383 | 1.876364193 |
| **TF@GL+NIR 4** | 0.116895256 | 0.038094199 | 1.658190431 |

To estimate the local H_2_Se concentration, the selenium content measured in bone tissues from inductively coupled plasma mass spectrometry (ICP-MS). Based on the net increase in selenium (ΔSe), the defect volume (23.56 mm^3^), and the molecular weights of H_2_Se (80.98 g mol^−1^) and Se (78.96 g mol^−1^), the local H_2_Se concentration at the bone defect was calculated to be 1.94 ± 0.25 μg mL^−1^.


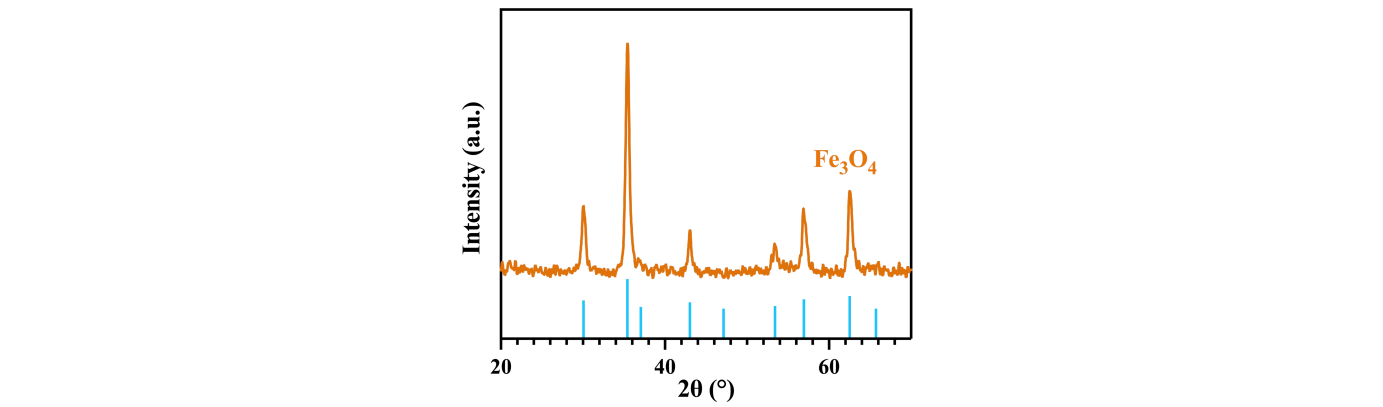


**Figure S1.** The X-ray diffraction (XRD) pattern of Fe_3_O_4_ NPs.

The six diffraction peaks corresponded to six crystal planes, indicating that the Fe_3_O_4_ NPs had a cubic crystal structure (Figure S1).


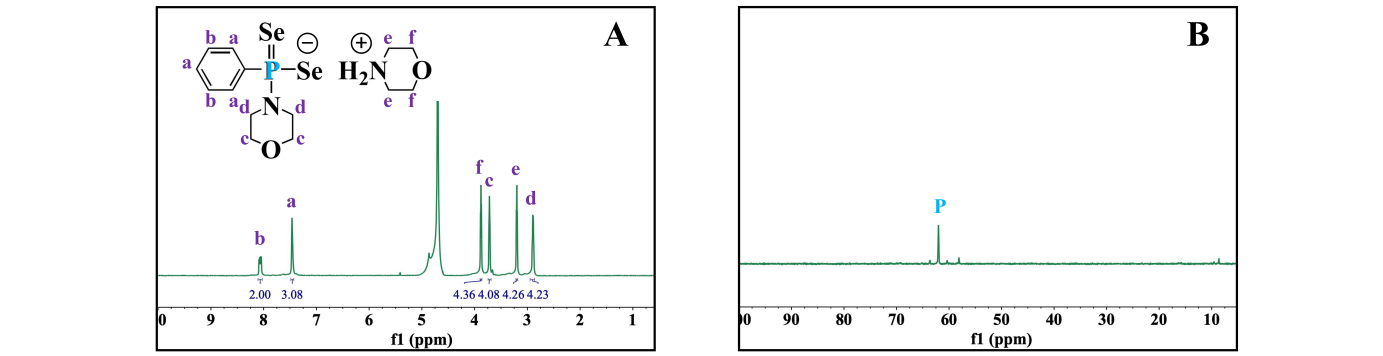


**Figure S2.** (A) ^1^H and (B) ^31^P NMR spectra of TDN1042.

The ^1^H NMR spectrum showed peaks at 8.05 ppm (b) and 7.45 ppm (a), corresponding to the protons on the meta and ortho carbon atoms of the benzene ring, respectively. The peaks at 3.87 ppm (f) and 3.70 ppm (c) were attributed to the protons on the methylene groups adjacent to oxygen in the six-membered ring, while the peaks at 3.23 ppm (e) and 2.87 ppm (d) corresponded to the protons on the methylene groups adjacent to nitrogen in the six-membered ring. By integrating the areas of these peaks, it was found that the number of hydrogen atoms matched theoretical predictions, indicating that TDN1042 had been successfully synthesized. Further structural validation was carried out using ^31^P NMR spectrum, where a strong peak at 62.12 ppm, along with weaker peaks on either side, corresponded to the vibrational peaks produced by the phosphorus atom located in the center of the TDN1042 molecule. The information presented in both spectra provided evidence that the H_2_Se donor TDN1042 had been successfully synthesized (Figure S2).


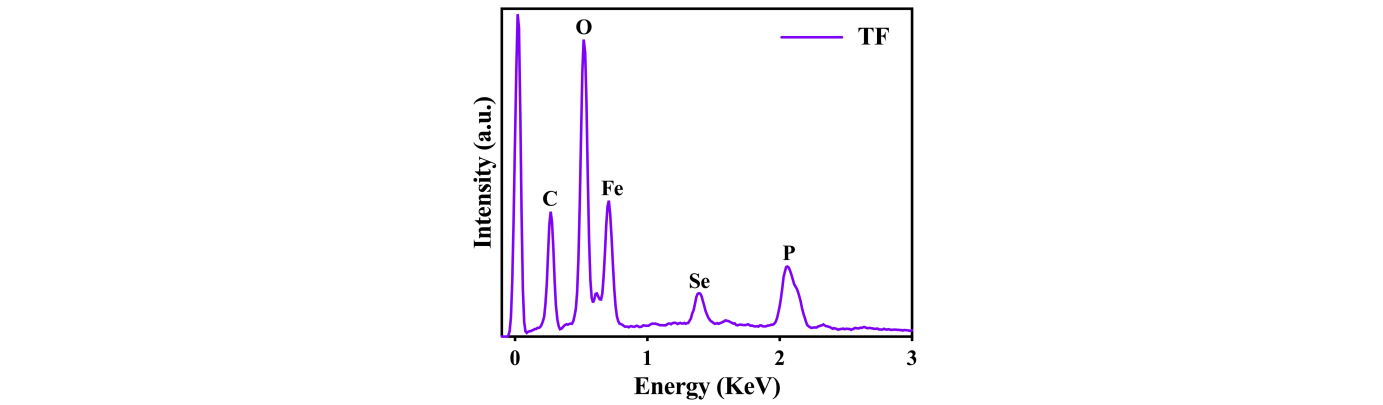


**Figure S3.** Energy dispersive spectrum (EDS) of TF NPs.

The EDS confirmed the presence of C, O, P, Fe and Se in TF NPs, indicating the successful encapsulation of H_2_Se donor (Figure S3).


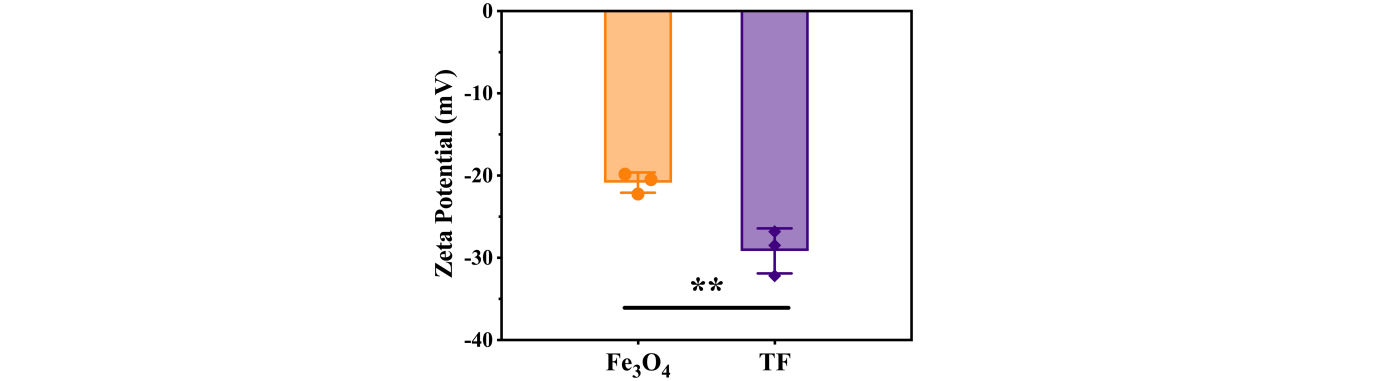


**Figure S4.** Zeta-potential of Fe_3_O_4_ and TF NPs (n=3). Data are presented as mean ± SD; *******P* < 0.01.

The surface potential of Fe_3_O_4_ NPs was -20.85 mV; after loading with the H_2_Se donor TDN1042, the surface potential of TF NPs was -29.16 mV (Figure S4). The increased negative charge enhanced the stability of the nanoparticles in aqueous solution, and loading TDN1042 improved the dispersion of the nanoparticles.


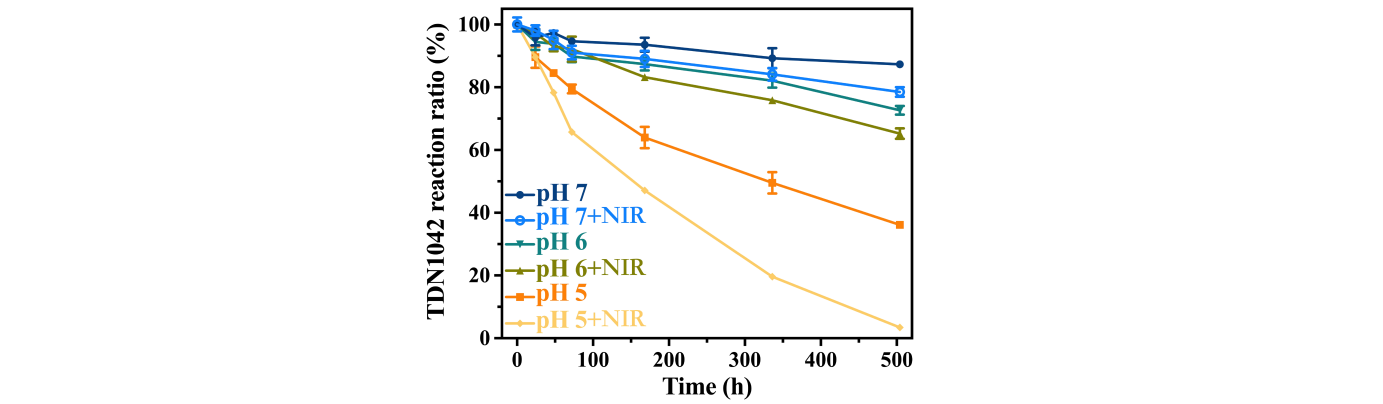


**Figure 5.** Hydrolysis curve of TDN1042 at different pH values, with or without NIR (n=3). Data are presented as mean ± SD.

To determine the effects of pH and temperature on the hydrolysis of the H_2_Se donor TDN1042, the hydrolysis rate of TDN1042 (10 mм) was measured with and without NIR exposure in citric acid buffer (50 mм) at pH 5.0, pH 6.0, and pH 7.0. The hydrolysis of TDN1042 was accelerated under mild photothermal conditions. Additionally, the hydrolysis rate of TDN1042 increased at more acidic pH levels (Figure S5).

**
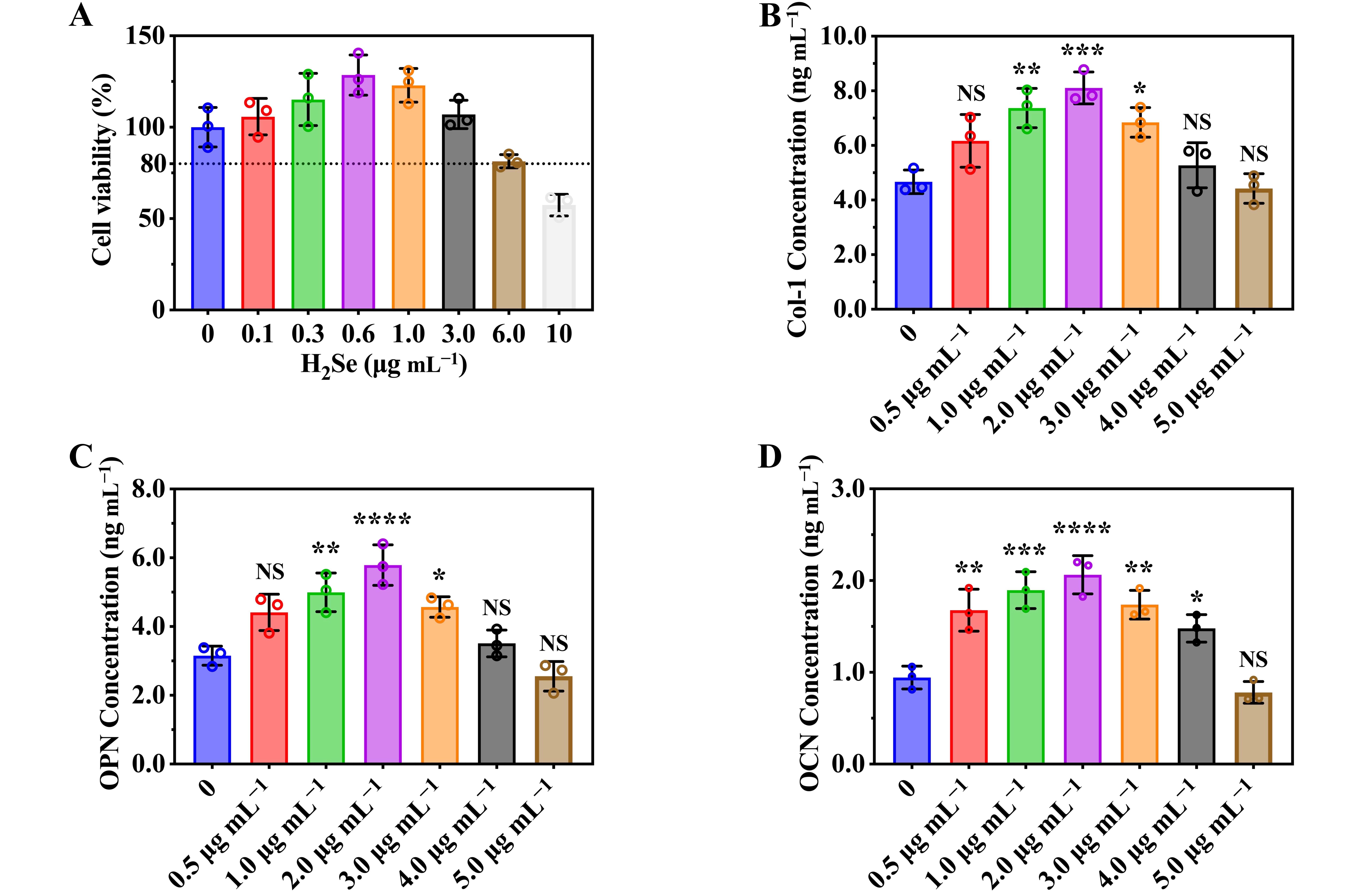
**

**Figure 6.** (A) Cell proliferation of BMSCs cultured with different concentration of H_2_Se for 24 hours (n=3). (B) ELISA analysis of Type I collagen (Col-1), osteopontin (OPN) and Osteocalcin (OCN) from BMSCs treated with different concentration of H_2_Se for 7 days (n=3). Data are presented as mean ± SD; ******P* < 0.05, *******P* < 0.01, ********P* < 0.001, *********P* < 0.0001.

To evaluate the dose-response of H_2_Se, BMSCs were treated with a concentration gradient (0–10 μg mL^−1^) of H_2_Se (Figure S6A). Cell viability remained above 90% in the 0–3.0 µg mL^−1^ range, indicating good biocompatibility at those concentrations. At 6.0 µg mL^−1^ cell viability dropped to about 80%, and at 10 µg mL^−1^ it fell to below 60%, indicating marked cytotoxicity. Therefore, the safety window for H_2_Se was estimated to be approximately 0–3.0 µg mL^−1^, within which significant cell damage was avoided.

Subsequently, expression levels of key osteogenic markers were assessed. (Figure S6B) Col-1 reached its highest levels at 1.0–3.0 µg mL^−1^ and was significantly higher than in the control, but its secretion declined when the concentration increased to 5.0 µg mL^−1^. OPN, and OCN showed similar trends, peaking at 1.0–3.0 µg mL^−1^ and weakening as concentration rose. When H_2_Se exceeded 5.0 µg mL^−1^, all osteogenic markers were suppressed to varying degrees, suggesting that excessive H_2_Se interfered with osteogenic differentiation because of cytotoxic effects. Collectively, H_2_Se exhibited both biocompatibility and osteogenic activity at the 1.0–3.0 µg mL^−1^, identifying this range as the optimal concentration window.


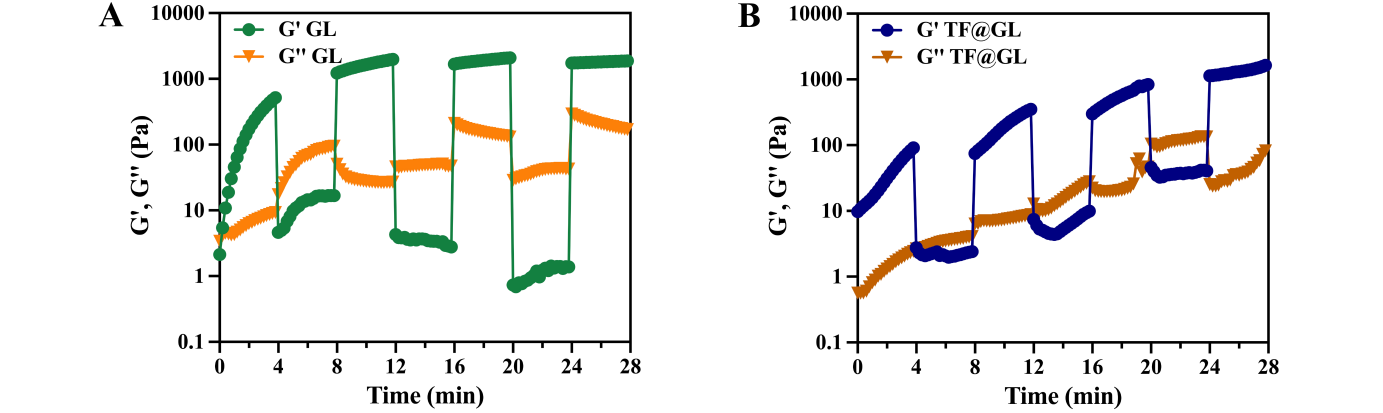


**Figure S7.** Rheology of Self-Healing Properties of (A) GL and (B) TF@GL hydrogel.

Based on macro-photographs of self-healing, rheological testing was conducted to evaluate the self-healing properties of GL and TF@GL hydrogels (Figure S7A, S7B). The G' of TF@GL gradually increased after each cyclic strain, while the G' of GL stabilized after the second cycle. This occurred because the first cycle (shortly after the onset of light exposure) involved extensive fracture and reorganization of disulfide bonds. In subsequent cycles, the crosslinked network of GL reached a dynamic equilibrium state. However, in TF@GL, the presence of nanoparticles may have physically restricted the contact between disulfide bonds, leading to lower initial crosslinking efficiency. Under photothermal effects, the localized temperature increase accelerated the motion of molecular chains, promoting the rate of disulfide bond fracture and reorganization, resulting in a continuous increase in G'. In addition, the G' of TF@GL was smaller than that of GL (contrary to results from conventional strain scanning and frequency scanning). This discrepancy arose from different testing conditions: in cyclic strain testing, mechanical stretching damaged the interfacial interactions between nanoparticles and the matrix, repeatedly disrupting the network structure. The weak interfacial region between the particles and the matrix could have become a stress concentration point, leading to energy loss and making it challenging to quickly achieve dynamic equilibrium within the testing duration.


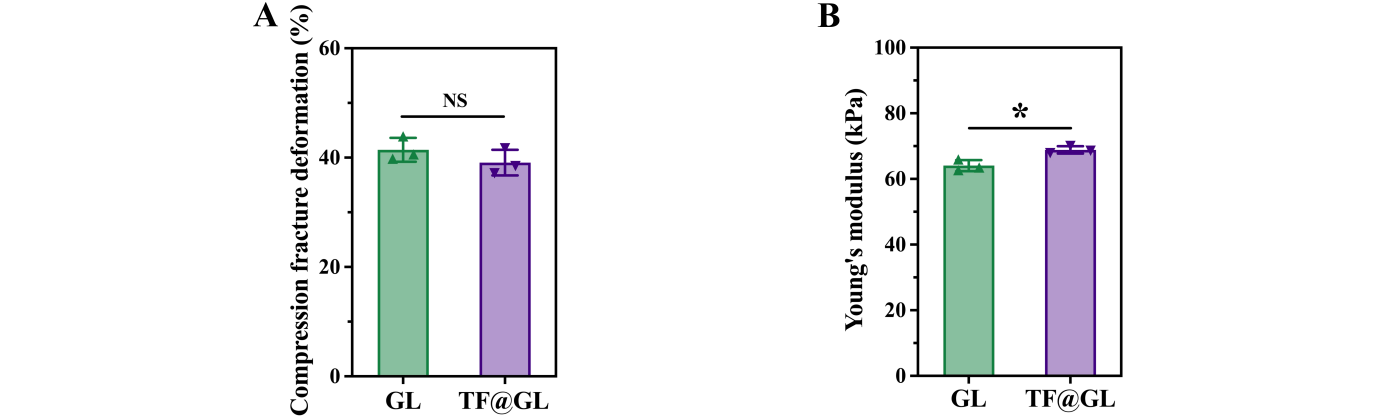


**Figure S8.** (A) Ultimate compressive strain, (B) the Young's modulus of GL and TF@GL hydrogel (n=3). Data are presented as mean ± SD; ******P* < 0.05.


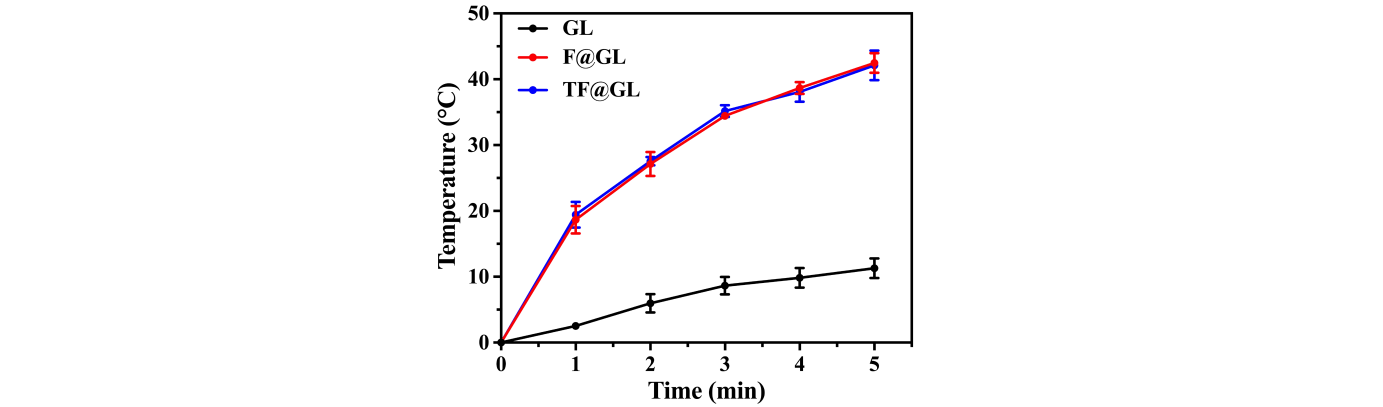


**Figure S9.** Temperature elevation curves of GL, F@GL, TF@GL hydrogel under NIR irradiation (808 nm, 1.2 W cm^−2^) (n=3). Data are presented as mean ± SD.


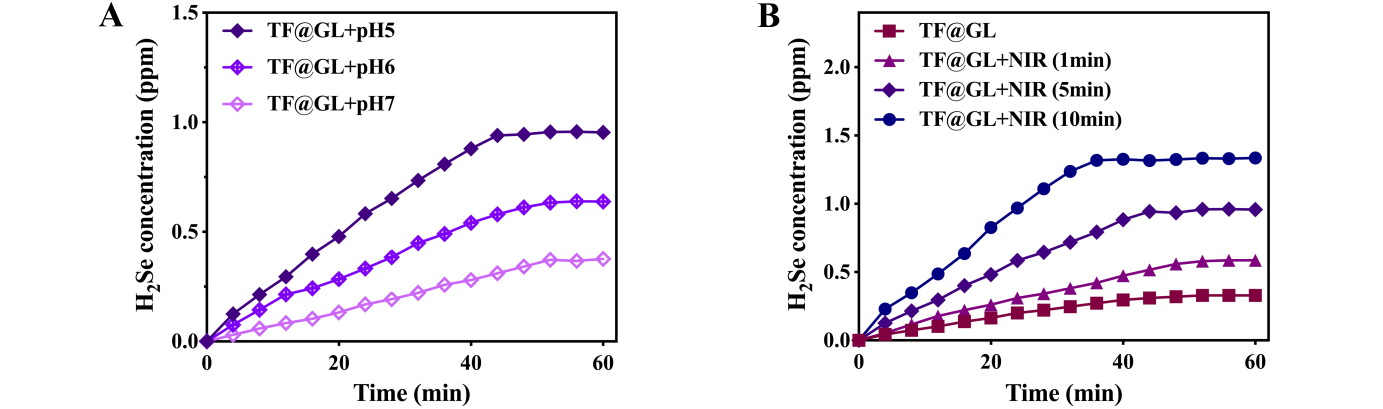


**Figure S10.** (A) Quantitative analysis of H_2_Se concentration from TF@GL hydrogel at different pH, with NIR (808 nm, 1.2 W cm^−2^, 5 minutes). (B) Quantitative analysis of H_2_Se concentration from TF@GL hydrogel at pH 5.0, with different durations of NIR (808 nm, 1.2 W cm^−2^).


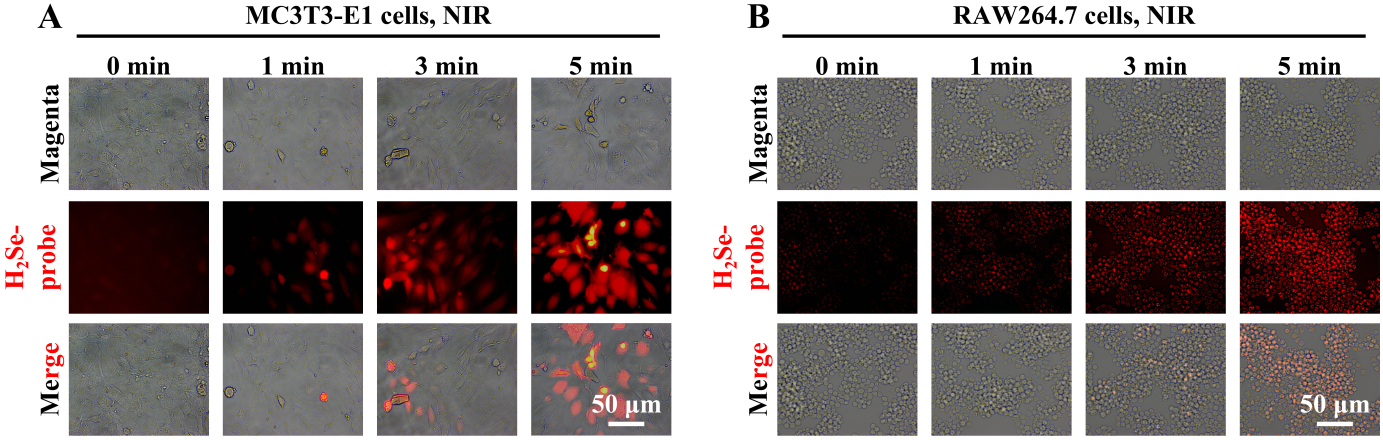


**Figure S11.** (A) In MC3T3-E1 and (B) RAW264.7 cells, thermocatalysis promoted the release of H_2_Se gas from the TF@GL hydrogel. Scale bars: 50 µm.

The concentration of H_2_Se in the cellular environment was investigated by varying the duration (0, 1, 3 and 5 minutes) of MPTT treatment (NIR, 808 nm, 1.2 W cm^−2^) to demonstrate whether the TF NPs have the capacity to thermally promote the release of H_2_Se. As shown in Figure S11A and S11B, without MPTT treatment, the red fluorescence intensity was very low, indicating the minimal release of H_2_Se. However, with increasing NIR treatment duration, the fluorescence intensity of the H_2_Se probe gradually increased, indicating the role of thermal synergistic control in the release of H_2_Se gas.


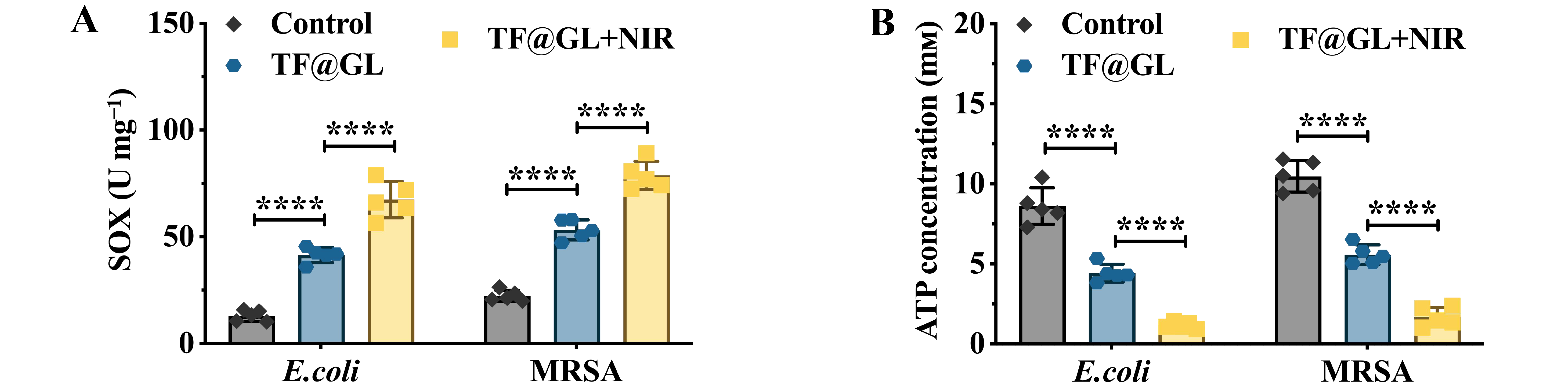


**Figure S12.** (A) Enzymatic activity of SOX (sulfur oxidoreductase), (B) ATP concentration of *E. coli* and MRSA bacterial colonies treated with the TF@GL hydrogel, with or without NIR (n=5). Data are presented as mean ± SD; *********P* < 0.0001.

Excessively activated SOX enzymes lead to the accumulation of toxic intermediates and disrupt the energy metabolism balance of bacteria. Specifically, the increased activity of SOX enzymes disrupts the dynamic balance of "substrate uptake, intermediate metabolism, and product release" during sulfur oxidation, resulting in the intracellular accumulation of intermediates that cannot be processed promptly by downstream enzymes. Elemental sulfur, as the primary product of SOX-catalyzed sulfide oxidation, can form insoluble particles within cells if its production rate greatly exceeds the metabolic capacity of downstream enzymes (e.g. sulfite oxidase). This accumulation can disrupt the fluidity and permeability of the cell membrane and hinder substance transport pathways.

Furthermore, the enhanced activity of SOX enzymes substantially increases the electrons released during the sulfur oxidation process. A large influx of electrons into the electron transport chain can result in electron accumulation because downstream receptors (e.g. denitrase or Cyt-c oxidase) cannot consume them promptly. This prevents the energy released from electron transport from effectively driving the establishment of a proton gradient, making it unavailable for ATP synthesis and dissipating solely as thermal energy. This vicious cycle of dramatically increased energy consumption and sharply decreased synthesis ultimately leads to stagnation in basic life activities, such as substance transport and macromolecular synthesis, due to energy deficiency, resulting in cell death.

To investigate the metabolic impact of H_2_Se on bacteria, we measured the activity of SOX and ATP content. Compared to the control group, the SOX activity of *E. coli* and MRSA significantly increased in the TF@GL and TF@GL+NIR groups (Figure S12A), while the ATP concentration in the TF@GL and TF@GL+NIR groups significantly decreased (Figure S12B). It revealed that H_2_Se upregulating SOX activity to disrupt bacterial energy metabolism, leading to metabolic collapse and bacterial death.


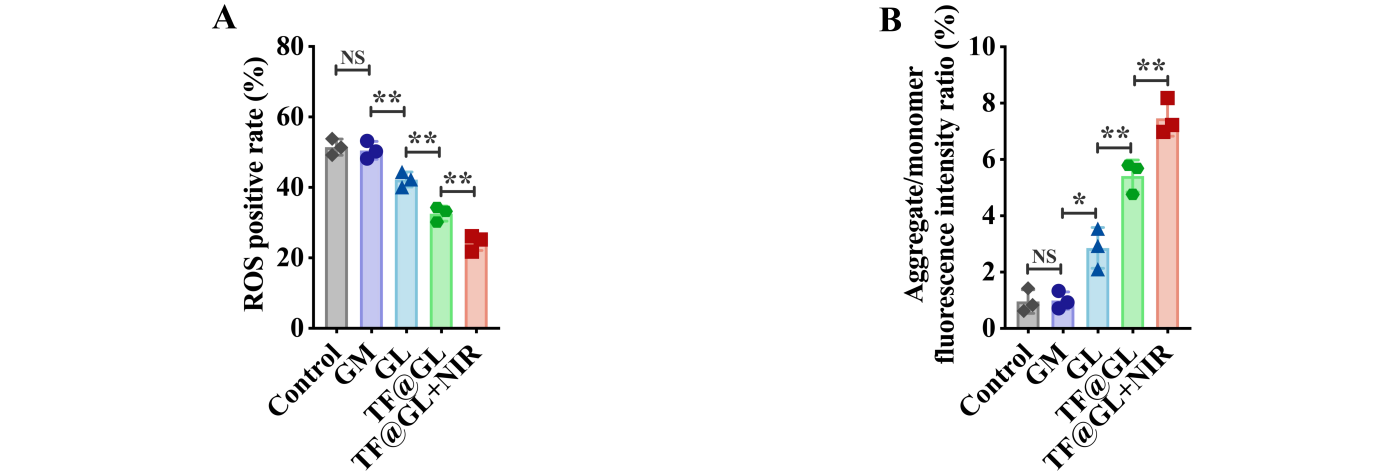


**Figure S13.** (A) Quantitation of ROS positive (DCFH^+^) cells (n=3). (B) Quantitation of aggregate/monomer flurescence intensity ratio of JC-1 staining (n=3). Data are presented as mean ± SD; ******P* < 0.05, *******P* < 0.01.


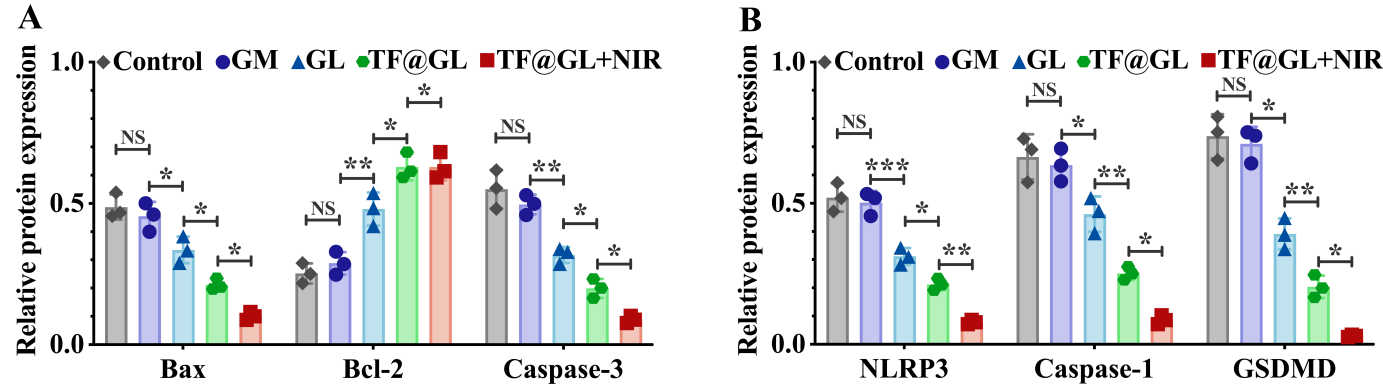


**Figure S14.** (A) Quantitative analyses of western blot of Bax, Bcl-2 and Caspase-3 (n=3). (B) Quantitative analyses of western blot of NLRP3, Caspase-1 and N-GSDMD (n=3). Data are presented as mean ± SD; ******P* < 0.05, *******P* < 0.01, ********P* < 0.001.


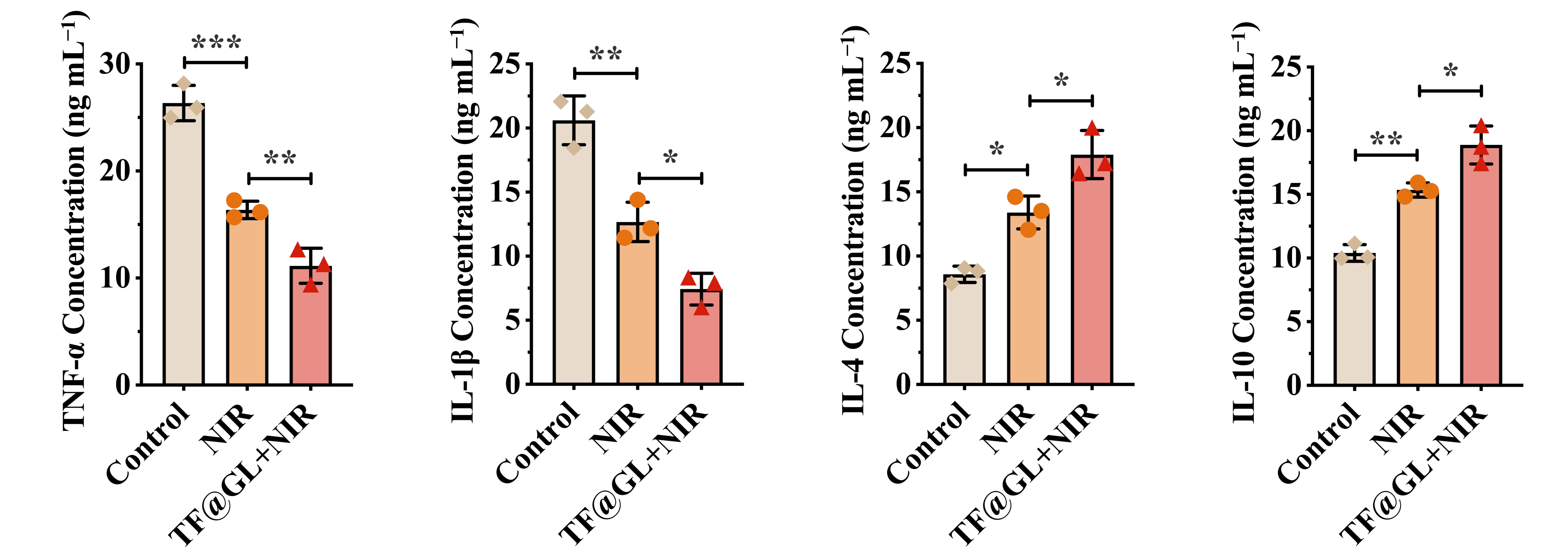


**Figure S15.** ELISA analysis of TNF-α, IL-1β, IL-4, IL-10 from RAW264.7 (after LPS stimulation) treated with control, NIR and TF@GL+NIR group for 48 hours (n=3). Data are presented as mean ± SD; ******P* < 0.05, *******P* < 0.01, ********P* < 0.001.

To dissect the immunomodulatory contributions of MPTT and H_2_Se release, we performed ELISA analysis of key cytokines in LPS-preactivated RAW264.7 macrophages. As shown in Figure S15, LPS stimulation induced robust secretion of pro-inflammatory cytokines TNF-α and IL-1β in the control group. Compared to the control group, cells exposed to NIR irradiation showed significant suppression of TNF-α and IL-1β secretion, along with elevated levels of the anti-inflammatory cytokines IL-4 and IL-10, demonstrating that mild photothermal stimulation exerts a measurable anti-inflammatory effect. Notably, the TF@GL+NIR group exhibited a stronger immunomodulatory response than the NIR group, with further reduction in TNF-α and IL-1β and markedly enhanced secretion of IL-4 and IL-10. These results indicate that on-demand H_2_Se release synergistically enhances the anti-inflammatory effects of mild photothermal therapy, promoting macrophage polarization toward a reparative M2 phenotype.


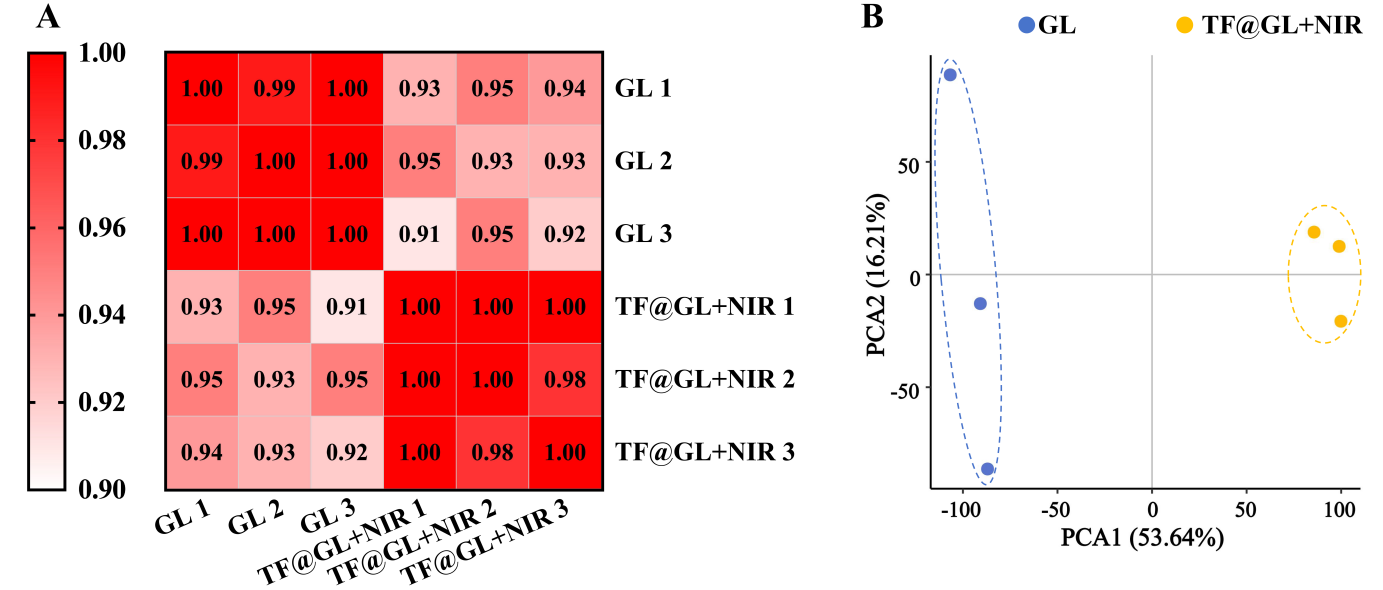


**Figure S16.** (A) Based on transcriptome sequencing of macrophages, the heatmap showed the pearson correlation between TF@GL+NIR and GL groups. (B) The principal component analysis (PCA) of TF@GL+NIR and GL groups.

The pearson correlation indicated that there was little variance within the samples of each group, while there were significant differences between the two groups (Figure S16A). The results of (PCA) showed that the two groups of samples were separately clustered, demonstrating that the samples met the necessary criteria (Figure S16B).


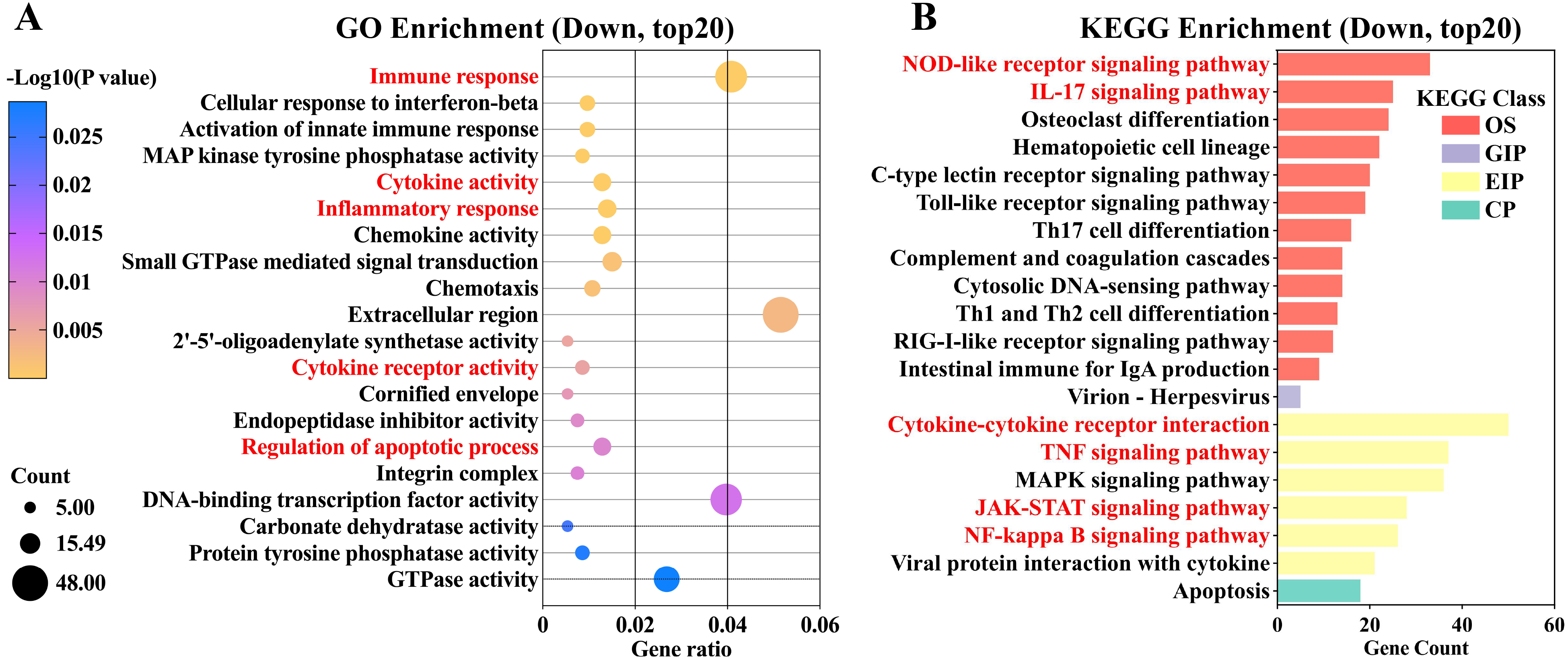


**Figure S17.** Transcriptome sequencing of macrophages. (A) Bubble chart of GO enrichment pathways were analyzed for TF@GL+NIR compared to GL, presenting the top 20 down-regulated pathways. (B) Clustering chart of KEGG enrichment pathways were analyzed for TF@GL+NIR compared to GL. The top 20 down-regulated pathways were classed as OS (organismal systems), GIP (genetic information processing), EIP (environmental information processing), CP (cellular processes).


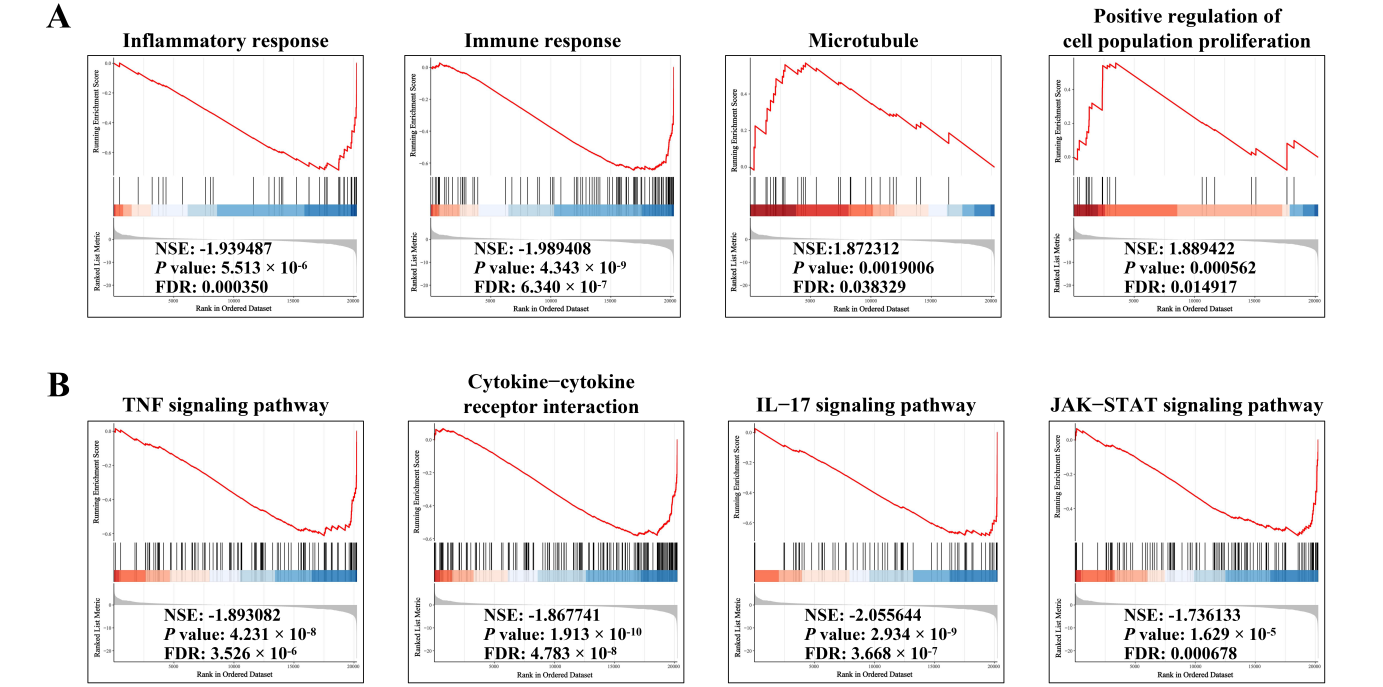


**Figure S18.** (A) GSEA of GO pathways. (B) GSEA of KEGG pathways.

The GSEA (gene set enrichment analysis) method was used to analyze the gene expression data for GO and KEGG enrichment. As shown in Figure S18A, the inflammatory response and immune response signaling pathways displayed downward curves, while the microtubule and positive regulation of cell population proliferation signaling pathways displayed upward curves in the GSEA graphs. As depicted in Figure S18B, the inflammatory response and immune response TNF, cytokine-cytokine receptor interaction, IL-17, and JAK-STAT signaling pathways displayed downward curves in the GSEA graphs. These results revealed that the TF@GL+NIR group reduced inflammatory and pyroptosis process. Interestingly, the TF@GL+NIR group has a certain promoting effect on cell proliferation.


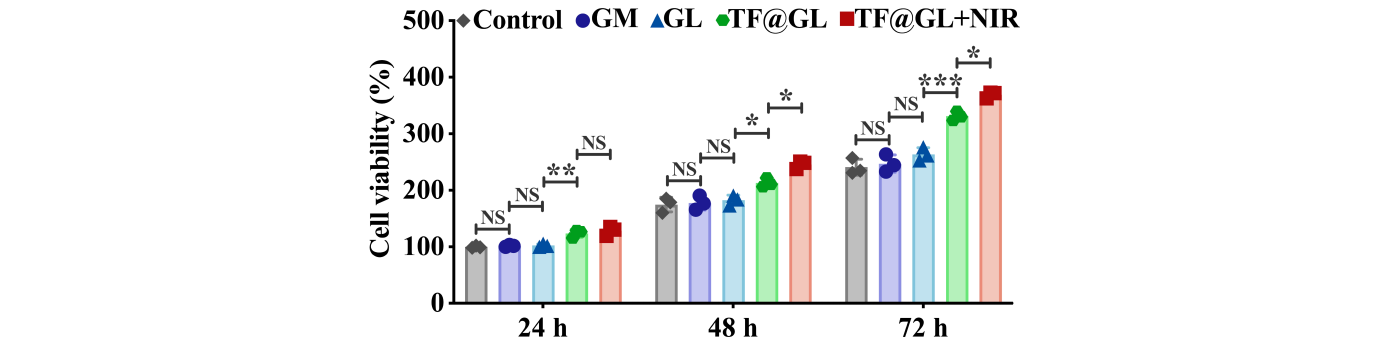


**Figure S19.** Cell proliferation of BMSCs cultured with GM, GL, TF@GL, and TF@GL+NIR hydrogels initially at 80% confluency for 24, 48 and 72 hours (n=3). Data are presented as mean ± SD; ******P* < 0.05, *******P* < 0.01, ********P* < 0.001.


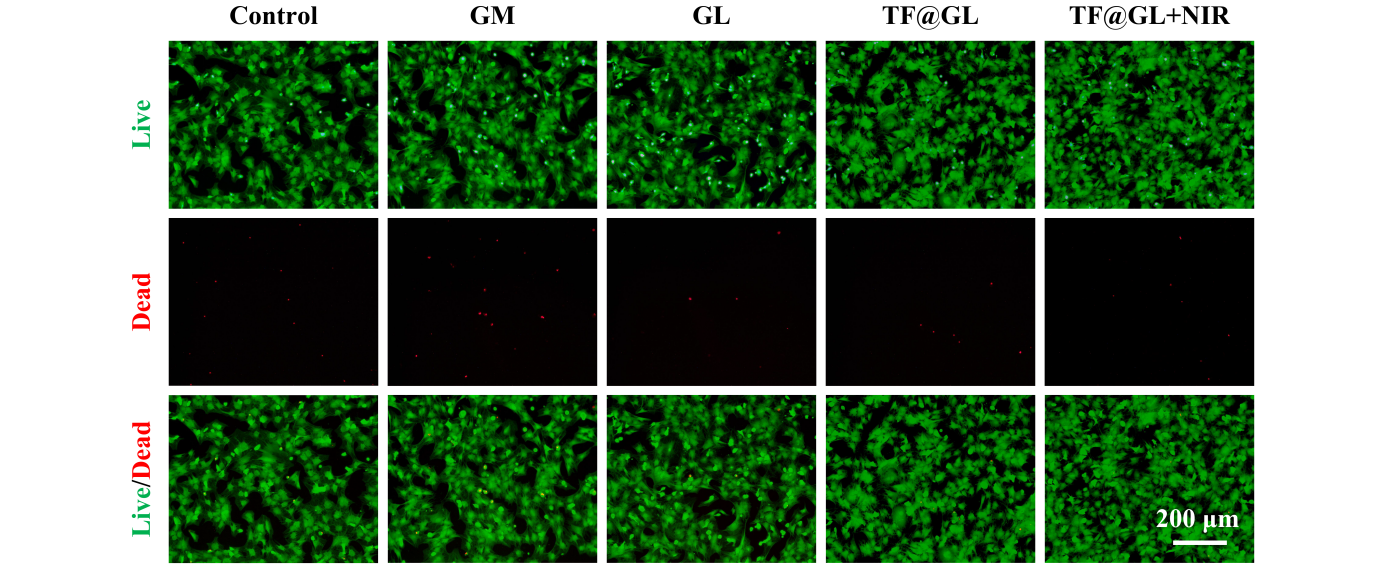


**Figure S20.** Live/dead staining of BMSCs cultured with different hydrogels initially at 80% confluency for 24 hours. Scale bars: 200 µm.


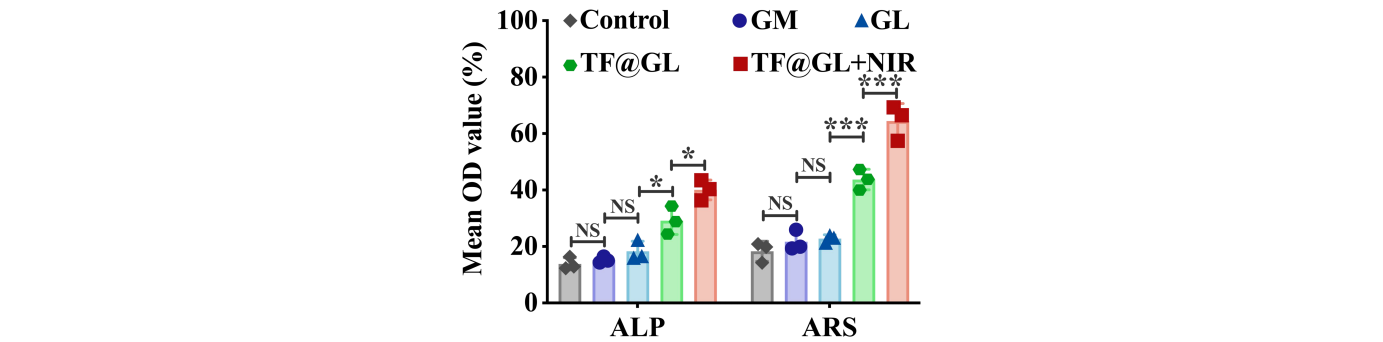


**Figure S21.** Quantification of ALP staining and Alizarin Red S staining (n=3). Data are presented as mean ± SD; ******P* < 0.05, *******P* < 0.01.

**
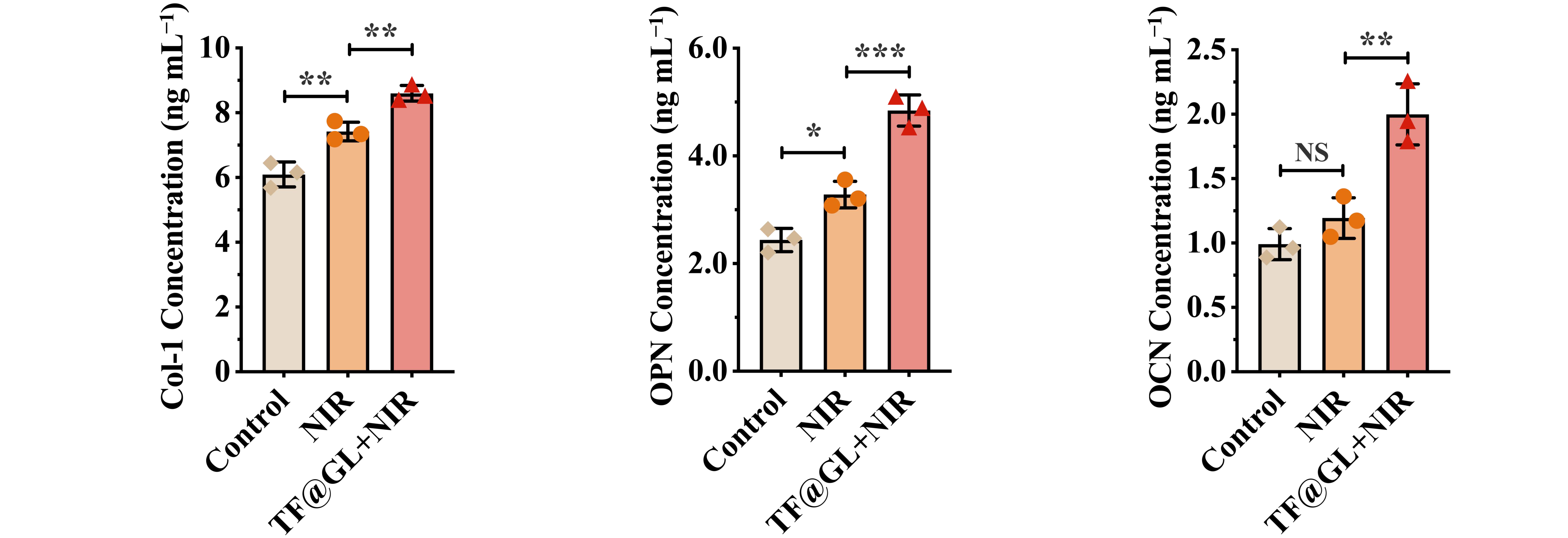
**

**Figure S22.** ELISA analysis of Col-1, OPN, OCN from BMSCs treated with control, NIR and TF@GL+NIR groups for 7 days (n=3). Data are presented as mean ± SD; ******P* < 0.05, *******P* < 0.01, ********P* < 0.001.

Given that MPTT inherently possessed a certain osteoinductive potential, we sought to clarify whether the observed enhancement in osteogenesis stemmed solely from H_2_Se release or was also influenced by the direct effects of MPTT on BMSCs. The secretion of key ECM proteins, including Col-1 (early marker), OPN (early marker), and OCN (late mineralization marker), was quantified (Figure S22). NIR irradiation alone moderately upregulated Col-1 and OPN compared to the control group, while OCN levels remained significantly unchanged, confirming that MPTT exerted only a modest, early-stage osteogenic effect. In contrast, the TF@GL+NIR group exhibited significantly elevated levels of all three markers, with secretion markedly higher than in the NIR group. These results demonstrated that H_2_Se not only amplified the initial matrix deposition initiated by MPTT but also played a crucial role in driving terminal mineralization, a step that MPTT alone could not achieve within a short timeframe. This highlighted underscoring the essential and synergistic role of H_2_Se in completing the osteogenic cascade.


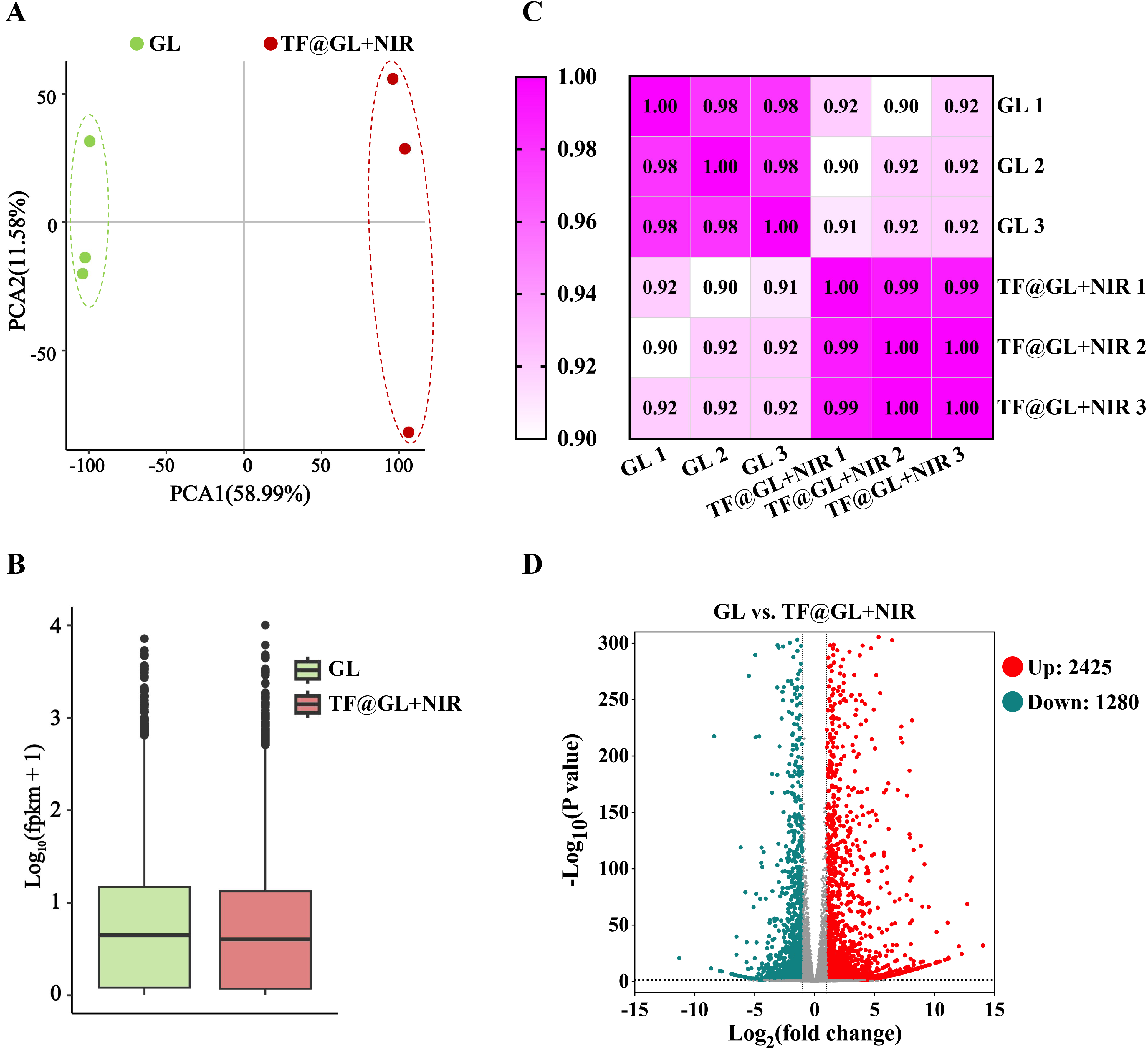


**Figure S23.** Bioinformatic analysis of BMSCs gene expression of TF@GL+NIR and GL groups. (A) The principal component analysis (PCA). (B) The FPKM box plot. (C) The heatmap of Pearson correlation. (D) The volcano plot of transcriptomic analysis of differentially expressed genes between TF@GL+NIR and GL groups.

The results of principal component analysis (PCA) showed that the two groups of samples were separately clustered (Figure S23A). The FPKM (Fragments Per Kilobase of exon model per Million mapped fragments) box plot demonstrated that the gene distribution of samples in the two groups is relatively consistent, with little fluctuation, in accordance with the principle of deriving from the same species of cells (Figure S23B). The pearson correlation revealed that the differences within the samples of each group were minimal, whereas there were distinct differences between the two groups (Figure S23C). In addition, the volcano plot illustrated 2425 up-regulated and 1280 down-regulated genes in the TF@GL+NIR versus GL group, indicating significant differences in gene expression (Figure S23D). These results demonstrated that the samples met the necessary criteria.


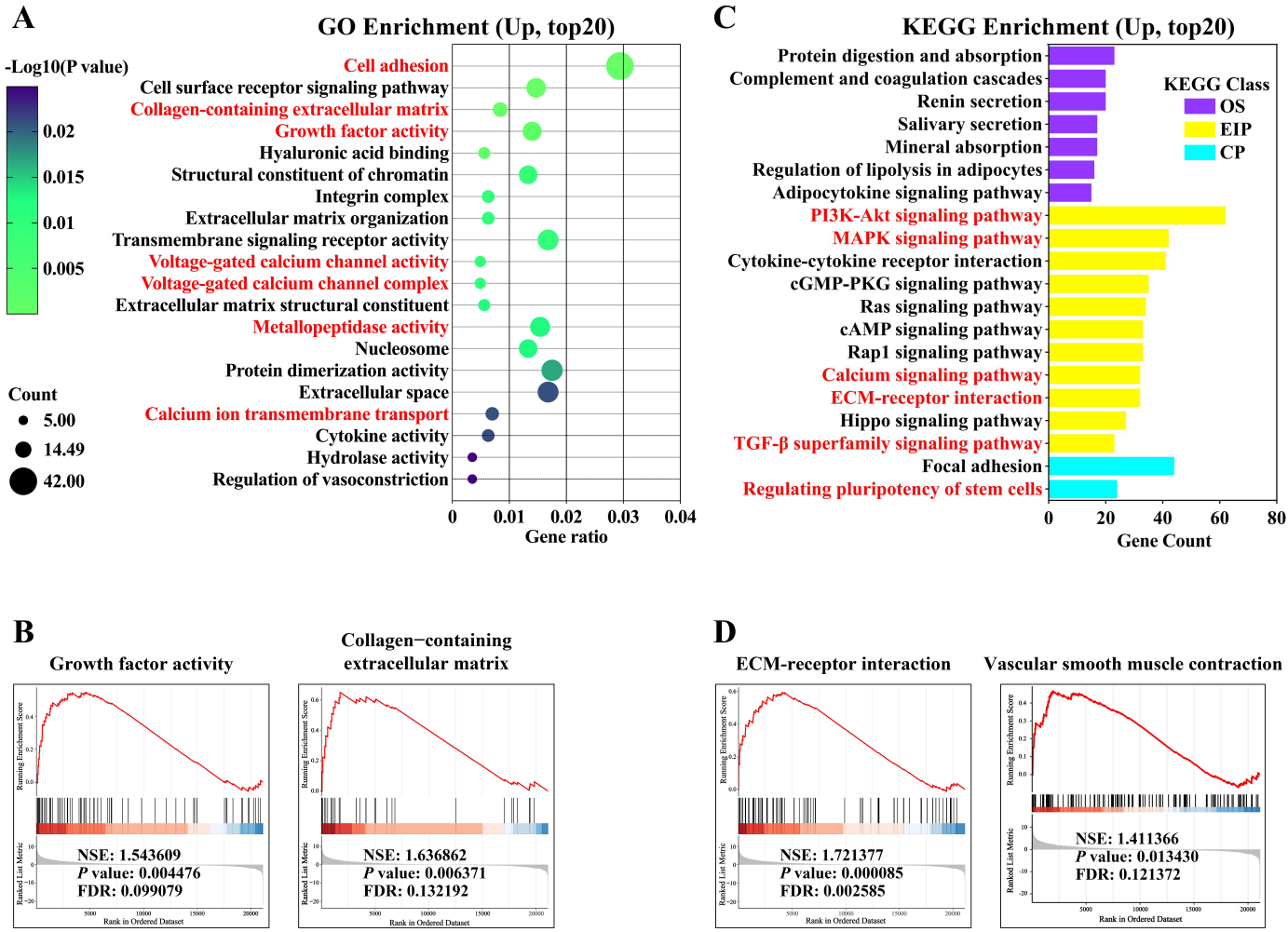


**Figure S24.** Bioinformatic analysis of BMSCs gene expression. (A) Bubble chart of GO enrichment pathways were analyzed between TF@GL+NIR and GL groups, presenting the top 20 up-regulated pathways. (B) GSEA of GO pathways. (C) Clustering chart of KEGG enrichment pathways were analyzed between TF@GL+NIR and GL groups. The top 20 up-regulated pathways were classed as OS (organismal systems), EIP (environmental information processing), CP (cellular processes). (D) GSEA of KEGG pathways.


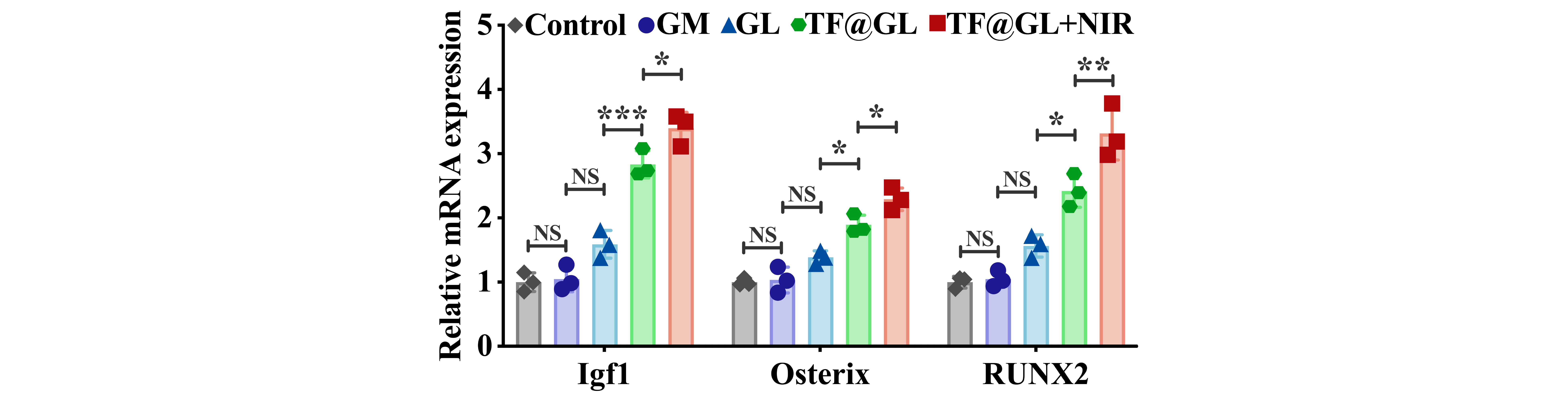


**Figure S25.** Relative mRNA expression of TGF-β/BMP and RPSC-related genes (n=3). Data are presented as mean ± SD; ******P* < 0.05, *******P* < 0.01, ********P* < 0.001.


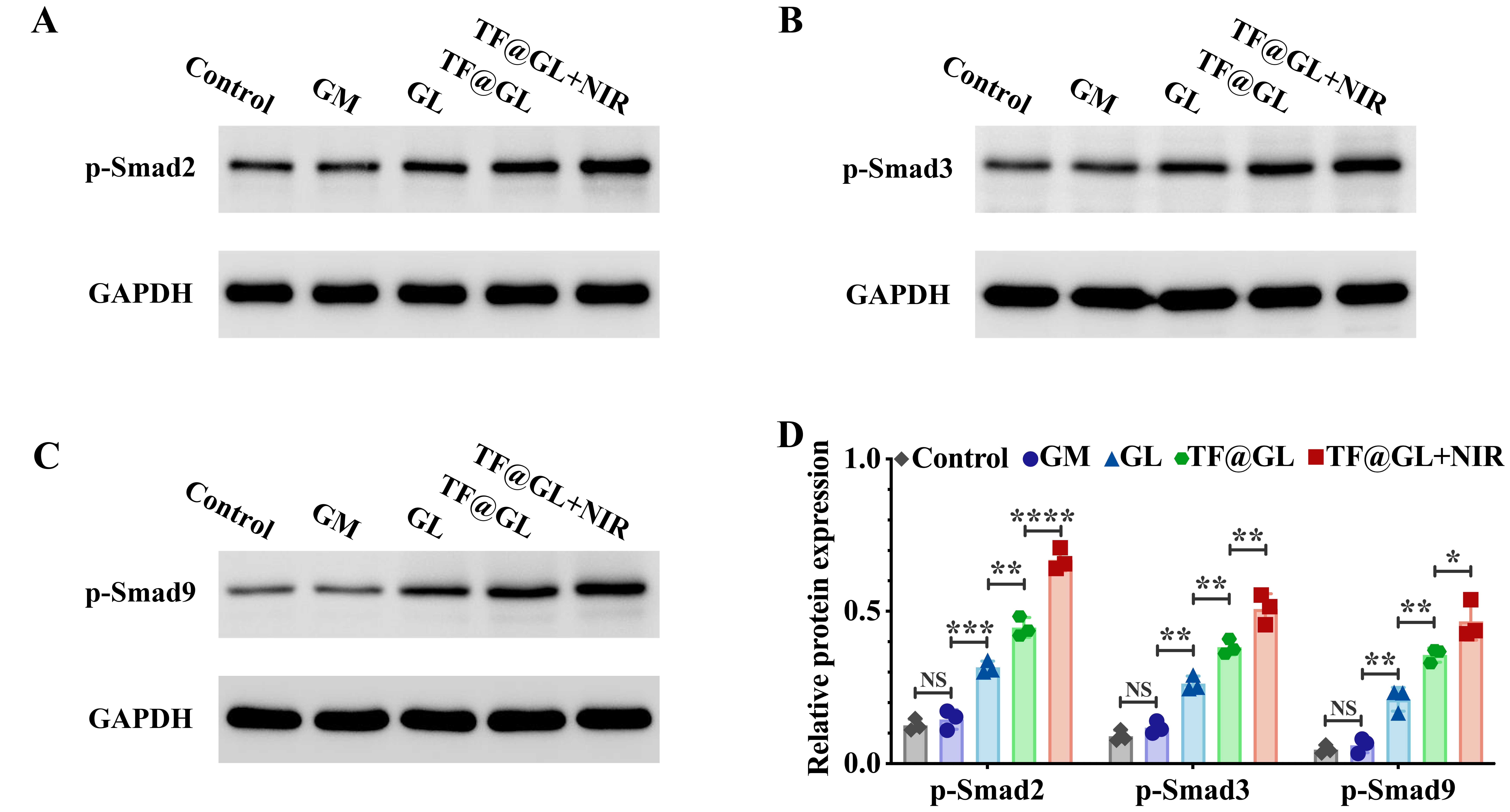


**Figure S26.** Western blot images of expression of TGF-β/BMP-related proteins of (A) p-Smad2, (B) p-Smad3 and (C) p-Smad9 in BMSCs treated with different hydrogels, and (D) relative protein expression (n=3). Data are presented as mean ± SD; ******P* < 0.05, *******P* < 0.01, ********P* < 0.001, *********P* < 0.0001.

Although TGF-β could exert pro-inflammatory effects in chronic inflammatory, the therapeutic action of H_2_Se released from the TF@GL hydrogel followed a temporally orchestrated sequence rather than simultaneous activation of conflicting pathways. In our experimental system, anti-inflammatory effects were evaluated at an early stage (at day 2) in LPS-primed macrophages, where H_2_Se effectively suppressed NF-κB and NLR signaling pathways and reduced pro-inflammatory cytokines, thereby establishing a reparative microenvironment. Subsequently, during the osteogenic phase (at day 7) in BMSCs, H_2_Se activated TGF-β signaling pathway to drove osteoblast differentiation and matrix mineralization. Because persistent inflammatory role of TGF-β could otherwise impair remodeling or promote bone resorption, the phased transition from inflammation resolution to osteogenic activation was critical for bone regeneration. Thus, H_2_Se could coordinate immune modulation and bone formation in a sequential manner.


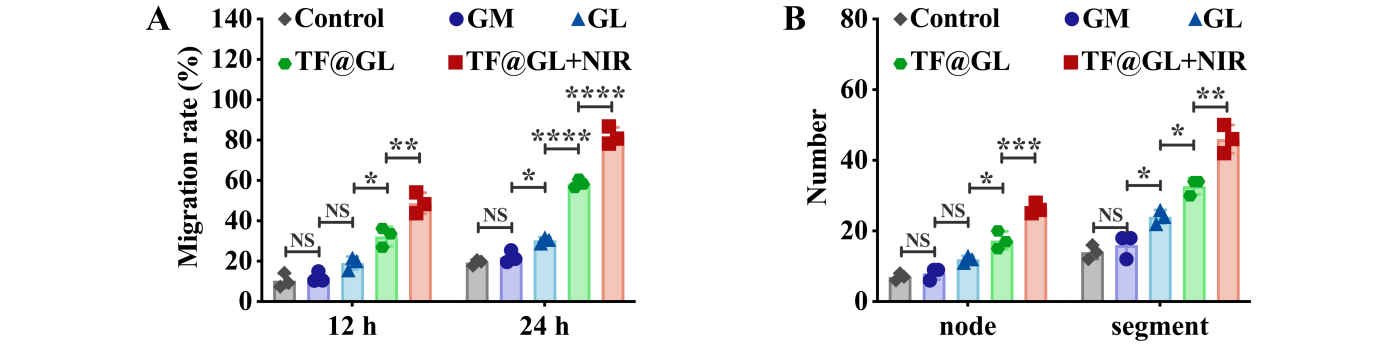


**Figure S27.** (A) Quantification of cell migration rate based on scratch wound healing tests (n=3). (B) Quantification of number of nodes and segments (n=3). Data are presented as mean ± SD; ******P* < 0.05, *******P* < 0.01, ********P* < 0.001, *********P* < 0.0001.


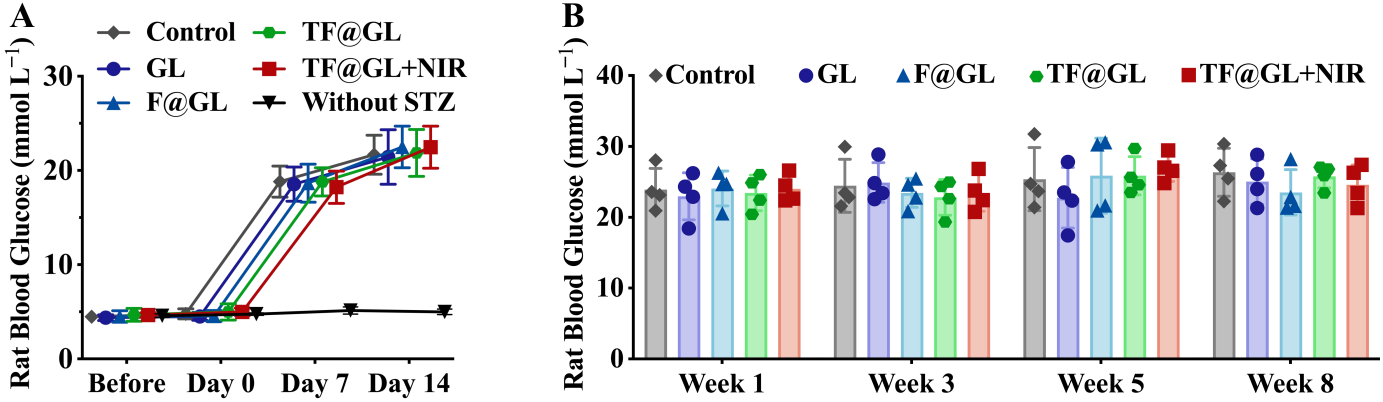


**Figure S28.** (A) Rat blood glucose of the control, GL, F@GL, TF@GL, TF@GL+NIR, and without STZ (streptozotocin) groups measured at several time points: before, after, on Day 7, and on Day 14 of STZ injection (n=4). (B) Rat blood glucose of the control, GL, F@GL, TF@GL, and TF@GL+NIR groups measured at week 1, 3, 5 and 8 post-treatment (n=4). Data are presented as mean ± SD.

SD rats were placed on a high-fat, high-sugar diet for 4 weeks to induce obesity and insulin resistance, conditions typical of type 2 diabetes. They then received a streptozotocin (STZ) injection, which selectively damaged the insulin-secreting β-cells in the pancreas, resulting in hyperglycemia. We utilized a glucometer to monitor blood glucose levels in this diabetic rat model, with measurements taken prior to injection, as well as on Days 7 and 14 post-STZ, to evaluate the progression of diabetes. Diabetes was confirmed based on persistent hyperglycemia, defined as blood glucose levels surpassing 16.8 mmol L^−1^ (300 mg dL^−1^) in two consecutive tests (Figure S28A). Strict exclusion criteria were implemented to ensure the reliability of our results; rats that displayed significant stress, abnormal glucose levels before the STZ injection, or any signs of illness that could affect metabolic evaluations were removed from the study. The diabetes induction success rate was approximately 88.18%, calculated based on the number of rats that sustained elevated blood glucose levels after applying the exclusion criteria throughout the study period.

At week 1, 3, 5, and 8 post-treatment, the blood glucose levels of rats in all groups consistently remained elevated (above 16.8 mmol L^−1^), confirming that all animals maintained a stable diabetic state during the full bone healing period (Figure S28B).


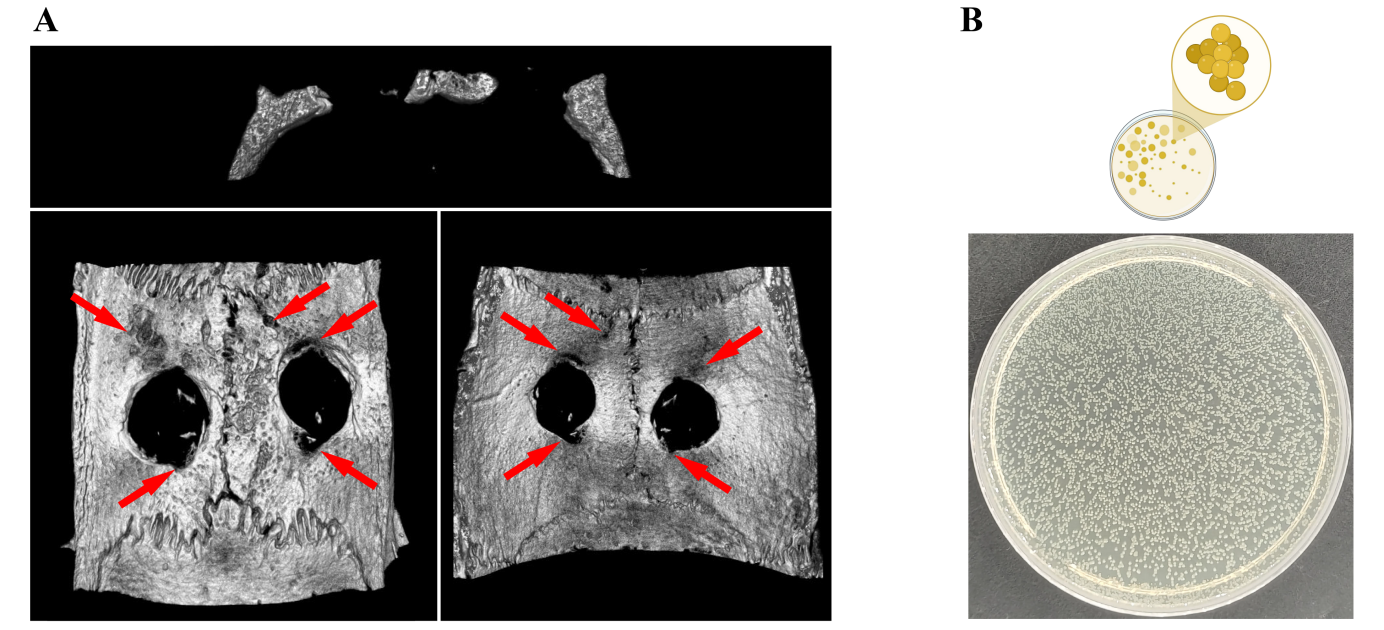


**Figure S29.** Modeling of Cranial Osteomyelitis. (A) Micro-CT image of the cranial defect at week 2 after the implantation of a gelatin sponge containing MRSA. The red arrows indicate areas of bone erosion and destruction. (B) MRSA collected from cranial tissue was cultured on an LB agar plate at week 2 post-implantation.

MRSA was chosen for modeling osteomyelitis, as it is the most common pathogen found in clinical practice. The modeling of cranial osteomyelitis in the rat model was successfully achieved through a combination of micro-CT imaging and tissue bacterial culture techniques. At 2 weeks post-implantation of a gelatin sponge infused with MRSA, micro-CT images revealed significant bone erosion and destruction, as indicated by the red arrows in the obtained images (Figure S29A). These findings corroborated the development of osteomyelitis in the cranial region. Additionally, MRSA was successfully isolated from the cranial tissue and cultured on an LB agar plate at the same time point, confirming the presence of the pathogen and supporting the effectiveness of the model for studying cranial osteomyelitis (Figure S29B). This model would be utilized for further research on the effects of TF@GL hydrogel in the treatment of cranial osteomyelitis.

Although the cranial osteomyelitis model provided an ideal platform for assessing the infection clearance and early immune modulation mediated by biomaterials, it was unable to fully replicate the complex pathological features of clinical diabetic osteomyelitis, such as the environment of diabetic foot osteomyelitis characterized by insufficient blood supply and mechanical stress. The abundant blood supply in the cranial region may have enhanced drug delivery efficiency and cellular recruitment, which may have influenced the therapeutic effects observed in the experiment. Therefore, future studies need to further validate the therapeutic effects of the TF@GL hydrogel in more clinically relevant animal models to comprehensively address the pathophysiological challenges of diabetic osteomyelitis.


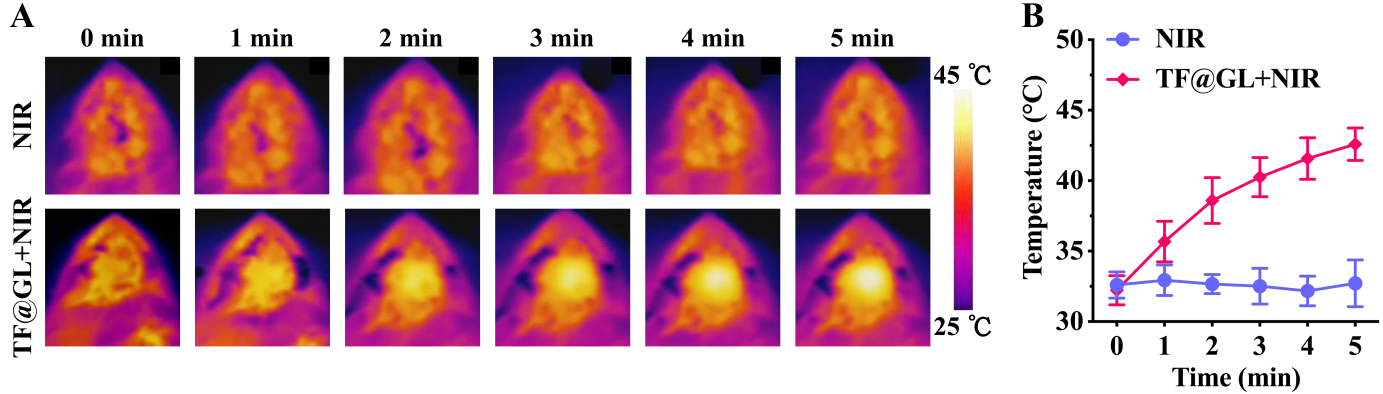


**Figure S30.** The thermal images of TF@GL hydrogel under NIR irradiation (808 nm, 1.2 W cm^−2^) in vivo. Data are presented as mean ± SD.

The thermal images showed that the TF@GL hydrogel heated effectively under NIR irradiation, confirming its photothermal responsiveness and providing a basis for on-demand activation of H_2_Se. This property was clinically significant, especially in scenarios requiring repeated NIR irradiation to maintain long-term therapeutic effects. For bone infections at different anatomical sites, corresponding NIR irradiation strategies could be employed.

In superficial sites such as the limbs or calvarium, TF@GL hydrogel was injected into the bone defect cavity following debridement and transcutaneous NIR irradiation was applied afterward. For deep-seated lesions such as those in the vertebrae or pelvis, minimally invasive techniques including percutaneous needle insertion were used to precisely position an optical fiber probe near the infected area to enable localized NIR irradiation. Moreover, the TF@GL hydrogel exhibited a certain retention at the defect sites, which supported repeated NIR activation and thereby sustained the controlled release of H_2_Se, maintaining both antibacterial and osteogenic effects.


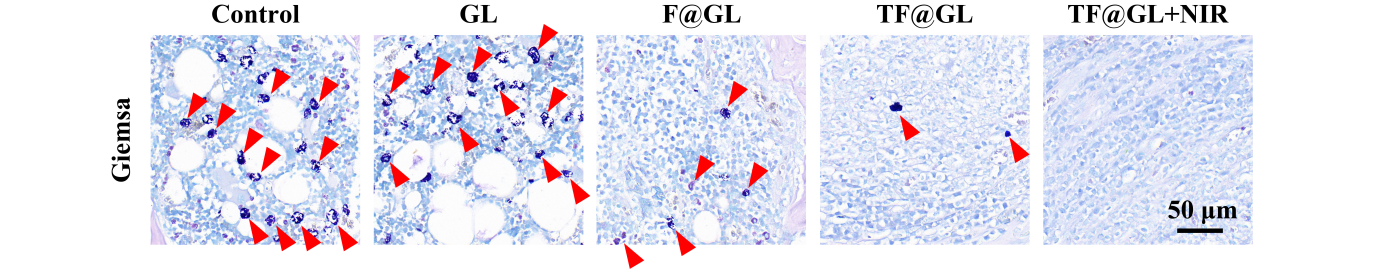


**Figure S31.** Giemsa staining of the control, GL, F@GL, TF@GL, and TF@GL+NIR groups in cranial defect at week 1 post-treatment. The red arrows indicate MRSA. Scale bars: 50 µm.


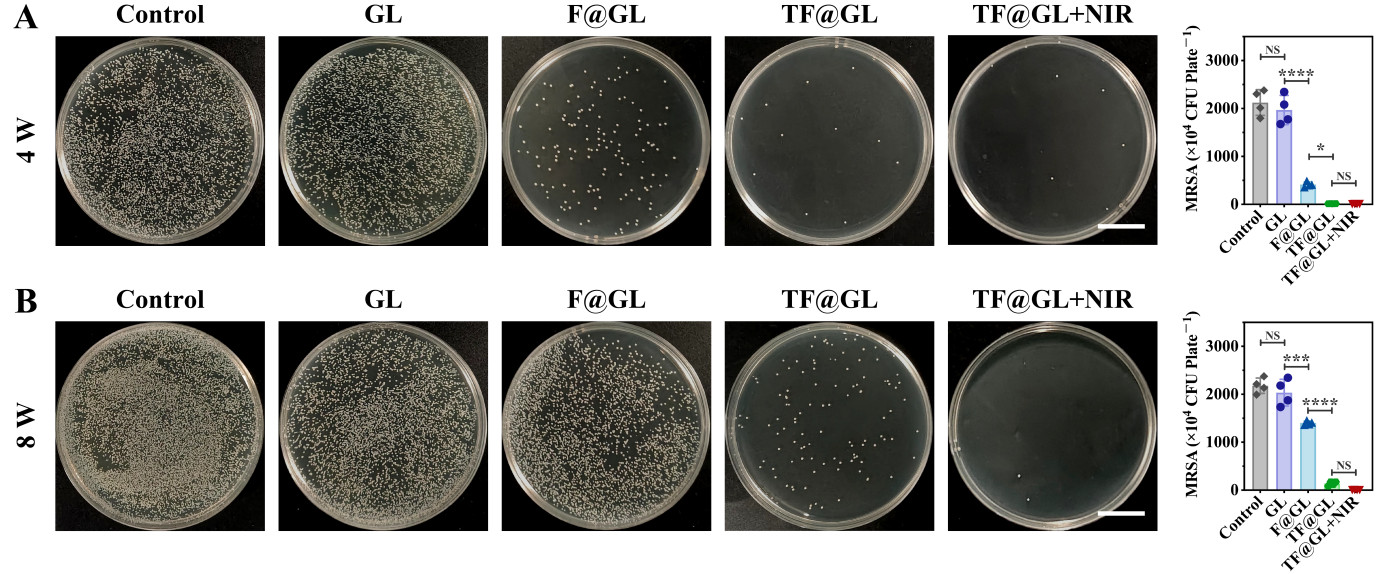


**Figure S32.** MRSA colonies and bacterial quantitation of infected cranium region at week 4 and 8 post-treatment in control, GL, T@GL, TF@GL, and TF@GL+NIR groups (n=4). Scale bars: 2 cm. Data are presented as mean ± SD; ******P* < 0.05, ********P* < 0.001, *********P* < 0.0001.

To evaluate the long-term antibacterial efficacy of each treatment group in the bone infection model, bacterial samples were collected from the cranial defect site and surrounding soft tissues at week 4 and 8 post-treatment, followed by quantitative culture and colony counting of MRSA. The results showed that the control and GL groups consistently displayed high MRSA burdens throughout the entire experimental period (Figure S32A, S32B). The F@GL group exhibited a marked increase in MRSA burden by week 8, with colony counts significantly higher than at week 4, suggesting insufficient durability of its antibacterial effect and clear evidence of infection relapse. Although the TF@GL group demonstrated robust antibacterial activity at week 4, a moderate increase in bacterial load was observed by week 8, indicating limited long-term stability against infection.

In contrast, at week 4, the MRSA colony counts in the TF@GL+NIR group were reduced to an extremely low level, significantly lower than those in the other groups. By week 8, the TF@GL+NIR group maintained excellent antibacterial performance, with MRSA colonies nearly undetectable in tissue samples, indicating that this treatment strategy not only achieved potent early-stage bacterial eradication but also effectively prevented long-term infection recurrence.


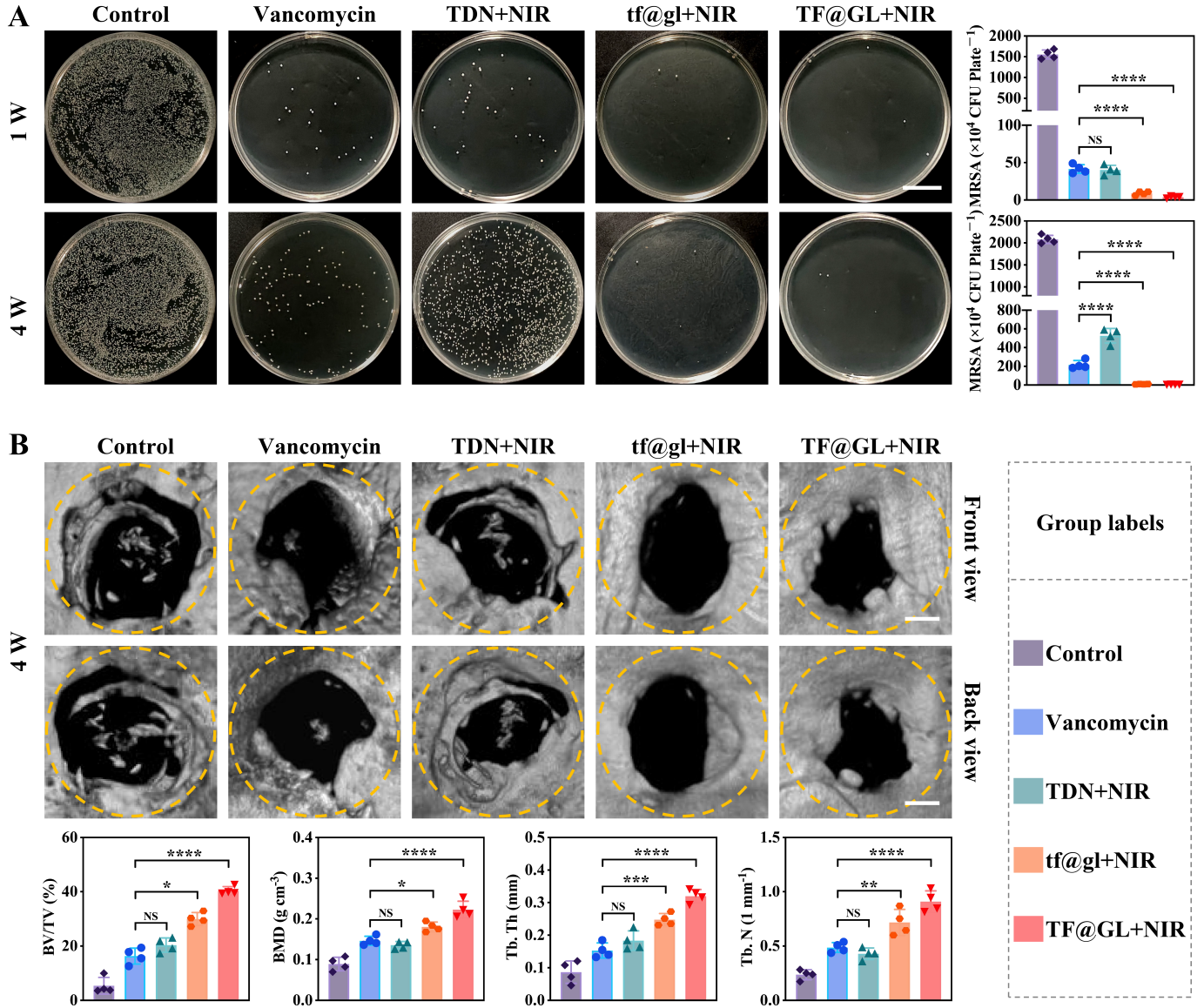


**Figure S33.** (A) MRSA colonies and bacterial quantitation of infected cranium region at week 1 and 4 post-treatment in control, Vancomycin, TDN+NIR, tf@gl+NIR, and TF@GL+NIR groups (n=4). Scale bars: 2 cm. (B) Micro-CT images of crania at week 4 post-treatment, and quantitative results of BV/TV, BMD, Tb.N, Tb. Th (n=4). Data are presented as mean ± SD; ******P* < 0.05, *******P* < 0.01, ********P* < 0.001, *********P* < 0.0001.

To further strengthen the experimental validation, we included three additional three groups for comparison: (1) Control: untreated infected group; (2) Vancomycin: vancomycin antibiotic treatment group, used for clinical efficacy comparison; (3) TDN+NIR: free TDN1042 (H_2_Se donor without a delivery system) combined with NIR irradiation, to evaluate the therapeutic effect of the H_2_Se donor alone; (4) tf@gl+NIR: TF@GL hydrogel lacking a microgel structure combined with NIR, to assess the contribution of the microgel's 3D network to therapeutic performance; (5) TF@GL+NIR: microgel-based TF@GL hydrogel combined with NIR irradiation, representing the optimized treatment formulation in this study.

The control group maintained a high bacterial burden at both time points, with persistent infection progression (Figure S33A). The vancomycin group exhibited potent antibacterial activity at week 1, but the bacterial load rebounded by week 4, indicating limited durability of its single dose, which is a clinical challenge. The TDN+NIR group also exhibited strong antibacterial activity, but infection clearly recurred by week 4, indicating that the delivery system is critical for controlled release or local retention of H_2_Se. Both the tf@gl+NIR and TF@GL+NIR groups demonstrated the strongest antibacterial effects, with significantly fewer bacterial colonies than the other three groups.

Micro-CT imaging and quantitative bone regeneration analysis were performed on cranial samples at week 4 post-treatment (Figure S33B). In the control group, irregular bone destruction was observed at the defect site, characterized by osteolytic areas and ill-defined radiolucent patches, consistent with active infection. The Vancomycin group showed limited new bone formation. The TDN+NIR group displayed irregular bone destruction alongside modest bone formation, likely due to initial infection control followed by recurrence. In contrast, the TF@GL+NIR group exhibited substantial new bone formation with well-defined margins, while the tf@gl+NIR group showed an intermediate effect. Bone regeneration parameters further supported these observations, including bone volume/tissue volume (BV/TV), bone mineral density (BMD), trabecular number (Tb.N), and trabecular thickness (Tb.Th). Although the Vancomycin group partially controlled infection, it resulted in limited bone regeneration. The TDN+NIR group showed no significant improvement over Vancomycin, suggesting that the free H_2_Se donor lacks sustained release capability and may only provide transient therapeutic effects insufficient for maintaining local action at the infection site. The tf@gl+NIR group displayed weaker bone repair capacity compared to the TF@GL+NIR group, further confirming that the microgel structure effectively supports bone regeneration within the local microenvironment. Overall, the TF@GL+NIR group achieved the best performance in both antibacterial activity and osteogenesis, highlighting the synergistic advantage of the integrated design combining H_2_Se donor and microgel-based structure.


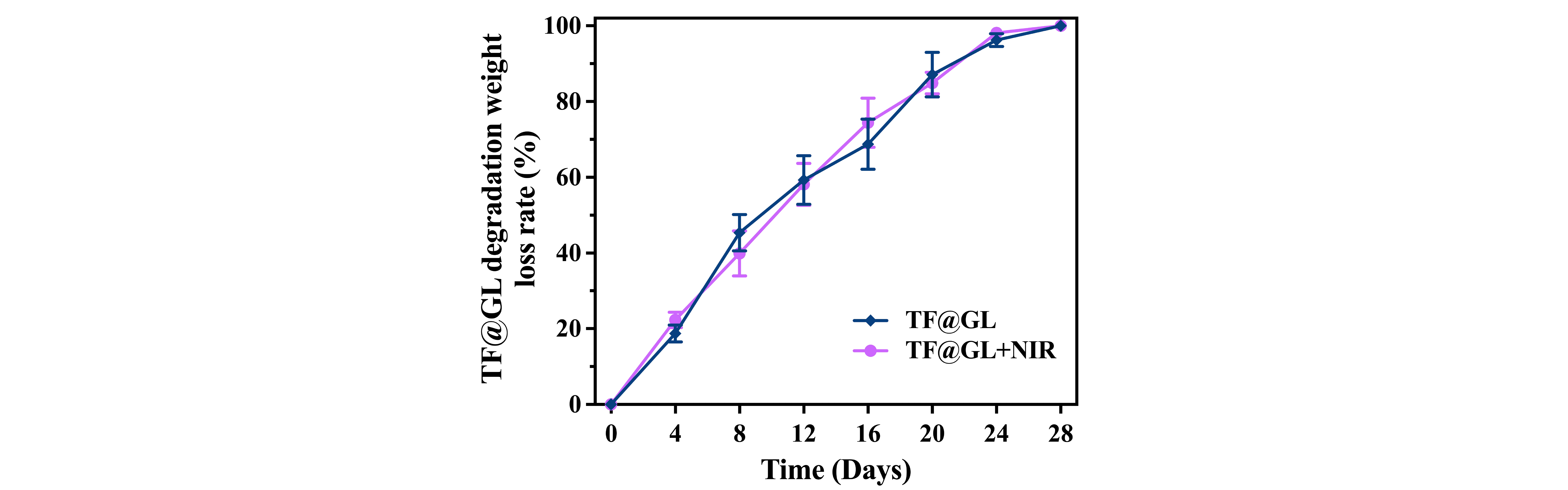


**Figure S34.** Degradation characteristics of TF@GL hydrogel in vivo (n=4). Data are presented as mean ± SD.

Degradation curves showed that the TF@GL and TF@GL+NIR groups degraded to 59.32 ± 6.43% and 58.16 ± 5.55% by day 12, and further degraded to 96.22 ± 1.70% and 98.14 ± 1.61% by day 24, approaching complete degradation (Figure S34). Both two groups completely degraded by day 28.


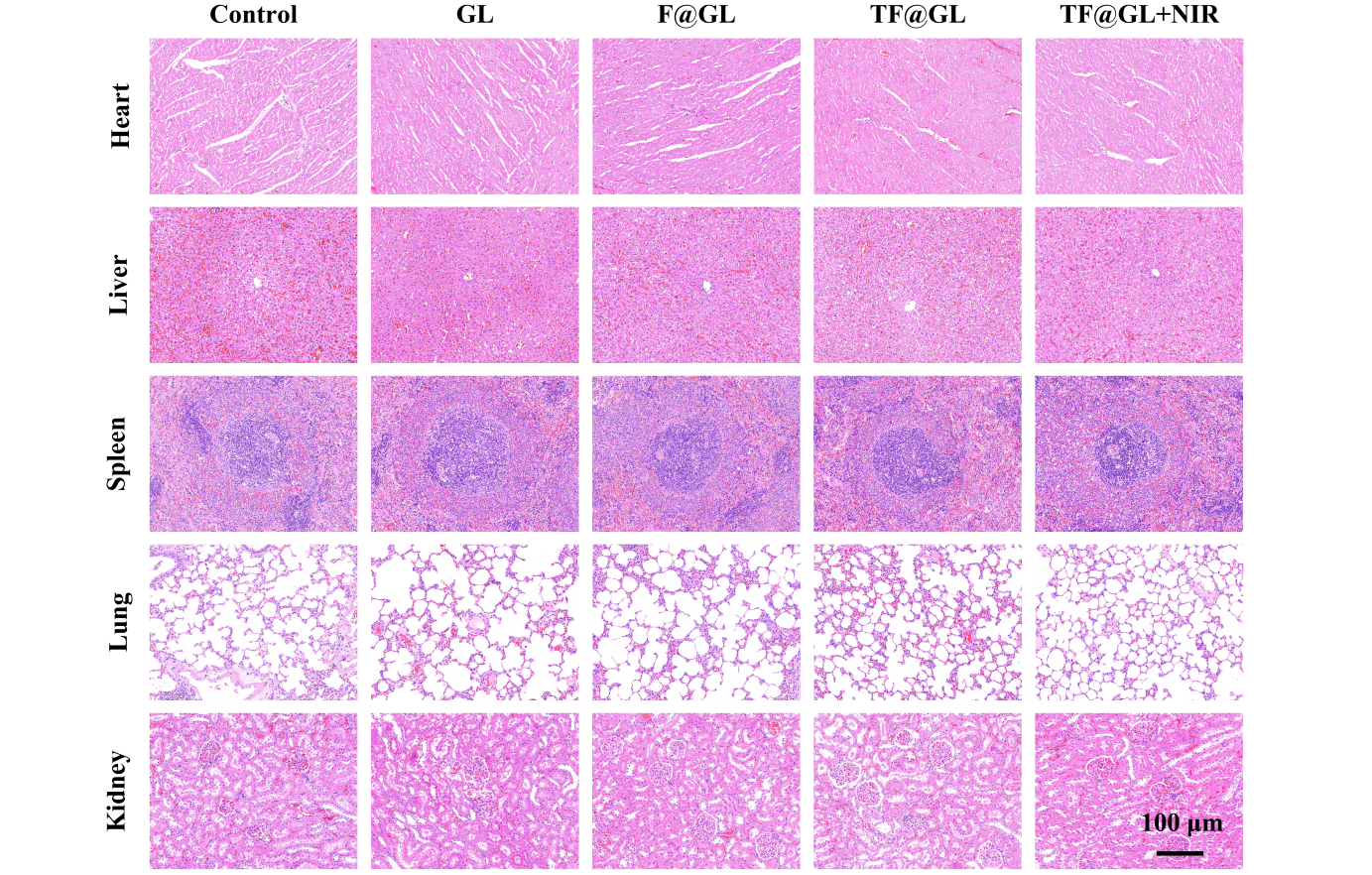


**Figure S35.** H&E staining for heart, liver, spleen, lung, and kidney tissues of the control, GL, F@GL, TF@GL, and TF@GL+NIR groups at week 8 post-treatment.

Histopathological examination of major organs, including heart, liver, spleen, lung, and kidney, by H&E staining showed no significant pathological abnormalities in the TF@GL group compared with the control group (Figure S35).


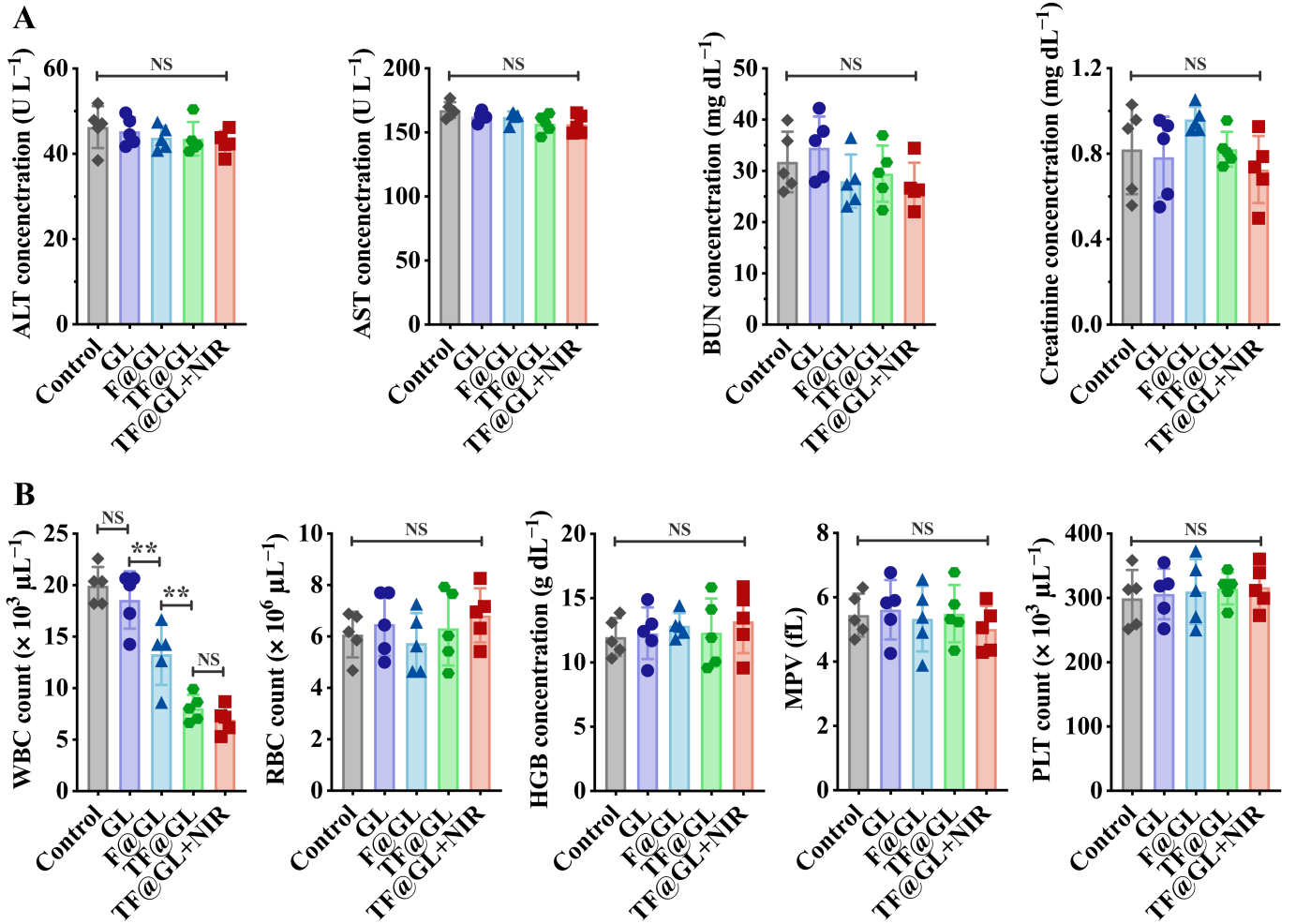


**Figure S36.** (A) Serum biochemical parameters, and (B) Hematological parameters measured at week 8 post-treatment (n = 5). Data are presented as mean ± SD; *******P* < 0.01.

Serum biochemical parameters, including alanine aminotransferase (ALT), aspartate aminotransferase (AST), blood urea nitrogen (BUN), and creatinine, all remained within normal reference ranges (Figure S36A). Hematological parameters, including red blood cell (RBC) count, hemoglobin (HGB), mean platelet volume (MPV), and platelet (PLT) count, were also within normal limits (Figure S36B). Notably, white blood cell (WBC) count was elevated above the normal range in the control group, consistent with chronic osteomyelitis, whereas it fell within the normal range in the TF@GL group, indicating effective resolution of infection.

**
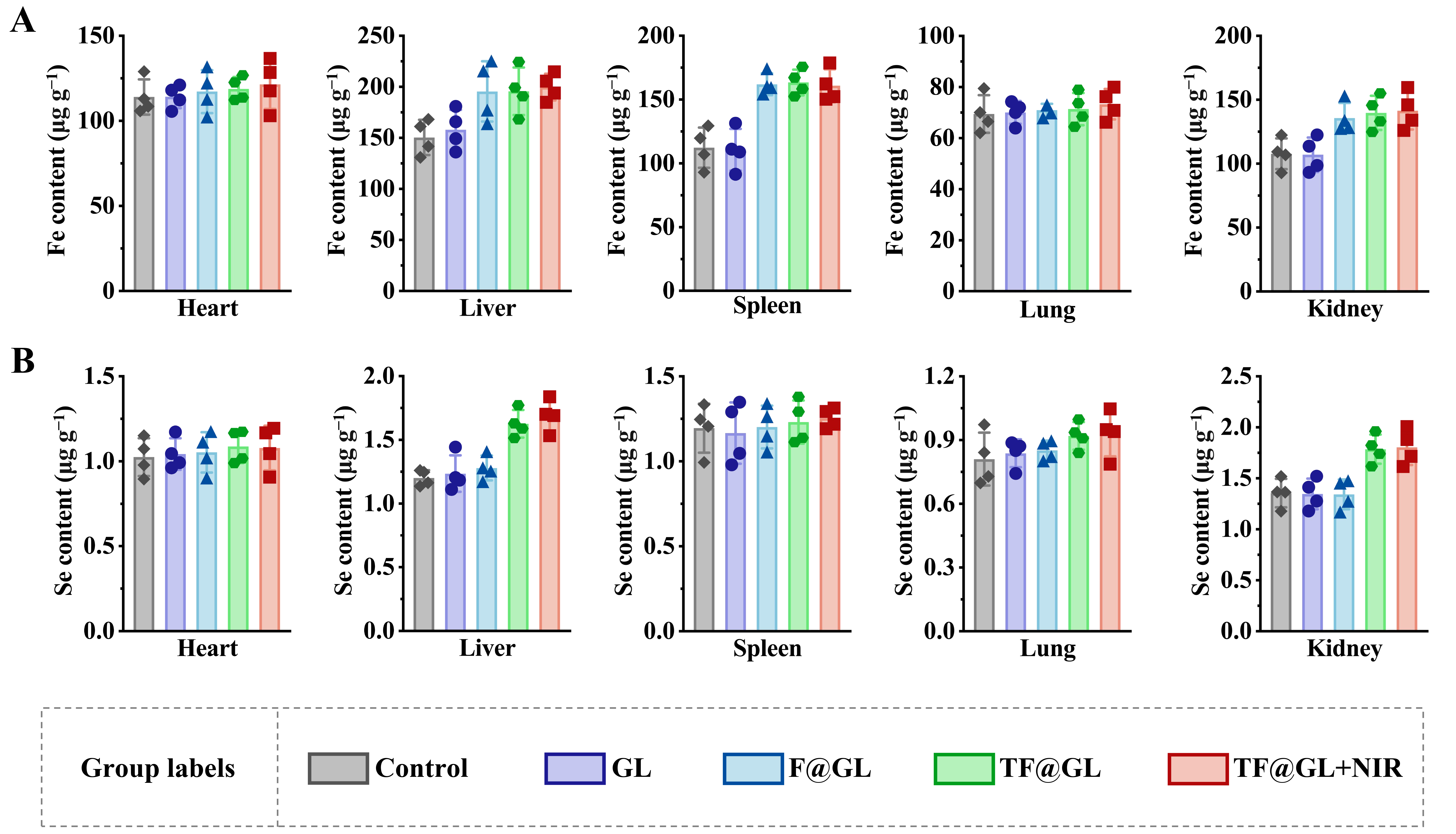
**

**Figure S37.** Detection of (A) Fe and (B) Se content in various organs of rats at week 8 post-treatment (n = 4). Data are presented as mean ± SD.

Fe and Se levels in major organs of rats were quantified by ICP-MS. Compared with the control group, the F@GL, TF@GL, and TF@GL+NIR groups showed moderate elevations in Fe content in the liver, spleen, and kidneys, while Fe levels in the heart and lungs did not differ appreciably among groups (Figure S37A). This indicated that Fe from the TF@GL hydrogel was mainly cleared through coordinated pathways of hepatic metabolism and storage, splenic recycling, and renal excretion.

Regarding Se, compared with the control group, the TF@GL and TF@GL+NIR groups showed slight increases in Se content in the liver and kidneys, while Se levels in the heart, spleen, and lungs did not differ appreciably among groups (Figure S37B). This suggested that Se from the TF@GL hydrogel was primarily metabolized via the liver and kidneys.


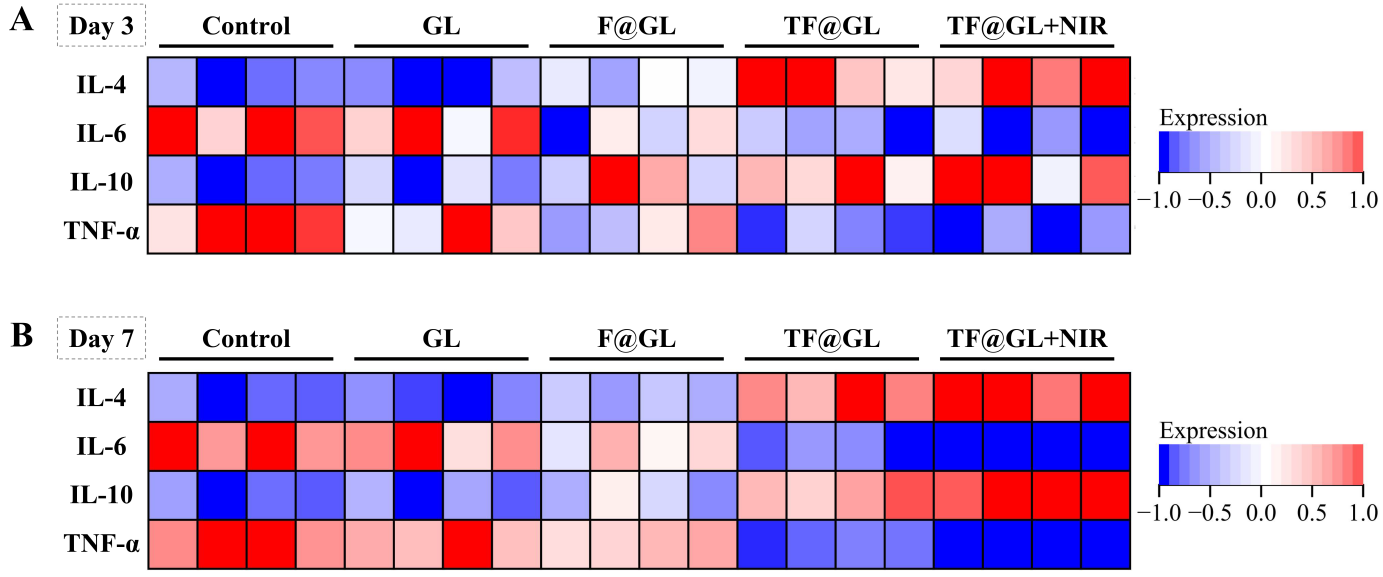


**Figure S38.** Heatmap of normalized ELISA results for inflammatory markers in rat serum at (A) day 3 and (B) 7 post-treatment (n = 4).


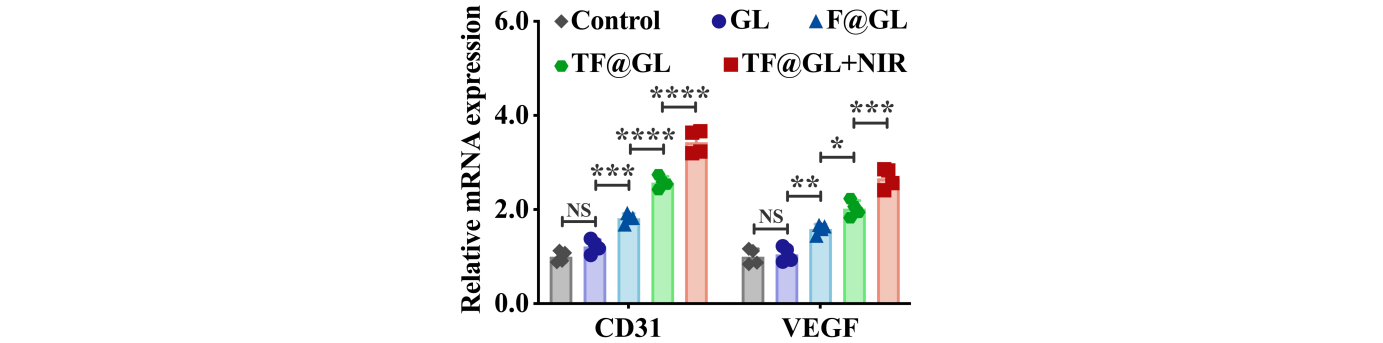


**Figure S39.** Tissues of the cranial defect at week 4 post-treatment. Relative mRNA expression of angiogenic genes CD31 and VEGF (n=4). Data are presented as mean ± SD; ******P* < 0.05, *******P* < 0.01, ********P* < 0.001, *********P* < 0.0001.
